# Association of Receiving Multiple, Concurrent Fracture-Associated Drugs With Hip Fracture Risk

**DOI:** 10.1001/jamanetworkopen.2019.15348

**Published:** 2019-11-13

**Authors:** Rebecca T. Emeny, Chiang-Hua Chang, Jonathan Skinner, A. James O’Malley, Jeremy Smith, Gouri Chakraborti, Clifford J. Rosen, Nancy E. Morden

**Affiliations:** 1 The Dartmouth Institute for Health Policy and Clinical Practice, Geisel School of Medicine, Dartmouth College, Lebanon, New Hampshire; 2 Division of Geriatric and Palliative Medicine, Internal Medicine, Institute of Healthcare Policy and Innovation, University of Michigan, Ann Arbor; 3 Center for Clinical and Translational Research, Maine Medical Center Research Institute, Scarborough; 4 now with Microsoft Artificial Intelligence and Research, Healthcare NeXT, Redmond, Washington

## Abstract

**Importance:**

Many prescription drugs increase fracture risk, which raises concern for patients receiving 2 or more such drugs concurrently. Logic suggests that risk will increase with each additional drug, but the risk of taking multiple fracture-associated drugs (FADs) is unknown.

**Objective:**

To estimate hip fracture risk associated with concurrent exposure to multiple FADs.

**Design, Setting, and Participants:**

This cohort study used a 20% random sample of Medicare fee-for-service administrative data for age-eligible Medicare beneficiaries from 2004 to 2014. Sex-stratified Cox regression models estimated hip fracture risk associated with current receipt of 1, 2, or 3 or more of 21 FADs and, separately, risk associated with each FAD and 2-way FAD combination vs no FADs. Models included sociodemographic characteristics, comorbidities, and use of non-FAD medications. Analyses began in November 2018 and were completed April 2019.

**Exposure:**

Receipt of prescription FADs.

**Main Outcomes and Measures:**

Hip fracture hospitalization.

**Results:**

A total of 11.3 million person-years were observed, reflecting 2 646 255 individuals (mean [SD] age, 77.2 [7.3] years, 1 615 613 [61.1%] women, 2 136 585 [80.7%] white, and 219 579 [8.3%] black). Overall, 2 827 284 person-years (25.1%) involved receipt of 1 FAD; 1 322 296 (11.7%), 2 FADs; and 954 506 (8.5%), 3 or more FADs. In fully adjusted, sex-stratified models, an increase in hip fracture risk among women was associated with the receipt of 1, 2, or 3 or more FADs (1 FAD: hazard ratio [HR], 2.04; 95% CI, 1.99-2.11; *P* < .001; 2 FADs: HR, 2.86; 95% CI, 2.77-2.95; *P* < .001; ≥3 FADs: HR, 4.50; 95% CI, 4.36-4.65; *P* < .001). Relative risks for men were slightly higher (1 FAD: HR, 2.23; 95% CI, 2.11-2.36; *P* < .001; 2 FADs: HR, 3.40; 95% CI, 3.20-3.61; *P* < .001; ≥3 FADs: HR, 5.18; 95% CI, 4.87-5.52; *P* < .001). Among women, 2 individual FADs were associated with HRs greater than 3.00; 80 pairs of FADs exceeded this threshold. Common, risky pairs among women included sedative hypnotics plus opioids (HR, 4.90; 95% CI, 3.98-6.02; *P* < .001), serotonin reuptake inhibitors plus benzodiazepines (HR, 4.50; 95% CI, 3.76-5.38; *P* < .001), and proton pump inhibitors plus opioids (HR, 4.00; 95% CI, 3.56-4.49; *P* < .001). Receipt of 1, 2, or 3 or more non-FADs was associated with a small, significant reduction in fracture risk compared with receipt of no non-FADs among women (1 non-FAD: HR, 0.93; 95% CI, 0.90-0.96; *P* < .001; 2 non-FADs: HR, 0.84; 95% CI, 0.81-0.87; *P* < .001; ≥3 non-FADs: HR, 0.74; 95% CI, 0.72-0.77; *P* < .001).

**Conclusions and Relevance:**

Among older adults, FADs are commonly used and commonly combined. In this cohort study, the addition of a second and third FAD was associated with a steep increase in fracture risk. Many risky pairs of FADs included potentially avoidable drugs (eg, sedatives and opioids). If confirmed, these findings suggest that fracture risk could be reduced through tighter adherence to long-established prescribing guidelines and recommendations.

## Key Points

**Question:**

Is the concurrent receipt of 2 fracture-associated drugs associated with increased risk of hip fracture?

**Findings:**

In this cohort study, 11 million person-years of Medicare data revealed that the receipt of multiple fracture-associated drugs was common among older US residents. Concurrent receipt of 2 or more such drugs was associated with a more than 2-fold increase in hip fracture risk, with some specific combinations appearing especially hazardous, including commonly prescribed drugs, such as opioids, antidepressants, and sedatives.

**Meaning:**

If confirmed, results of this cohort study suggest caution when combining fracture-associated medications, especially when use is discretionary, alternatives exist, or baseline fracture risk is high.

## Teaser

This cohort study estimates hip fracture risk associated with concurrent exposure to multiple fracture-associated drugs.

## Introduction

At least 21 prescription drug groups have been associated with increased fracture risk.^1,2,3,4,5,6,7,8,9,10,11,12^ The list includes some of the most commonly used products in the United States, such as opioids, proton pump inhibitors (PPIs), serotonin reuptake inhibitors (SSRIs), and sedatives.^13^ The wide use of many fracture-associated drugs (FADs) suggests that concurrent receipt of 2 or more may be prevalent. This in turn raises concern for fracture risk associated with such exposure. Logic suggests that risk will increase with the addition of each additional FAD. However, the fracture risk of such combined drug use has not been quantified. Understanding the prevalence of concurrent FAD exposure and its risk is especially important to older people, who have the highest baseline rate of fragility fracture and who are most likely to receive multiple FADs as diagnoses and treatments accumulate with age.

Research suggests individual FADs increase fracture risk by weakening bones, increasing falls, or both. Pathways of bone weakening may involve direct skeletal cell effects, disruption of vitamin D or calcium management, modulation of parathyroid hormone effects, and hypogonadism.^14,15,16,17,18,19,20,21^ The mechanisms by which medications increase falls include sedation, loss of balance, dizziness, visual disturbance, muscle weakening, and hypotension.^6,9,22,23,24^ A total of 8 drug groups have been associated with both bone weakening and falls (eTable 1 in the Supplement). The diversity of mechanisms makes the net effect of exposure to multiple FADs difficult to predict. Risk associated with exposure to 2 FADs acting through the same mechanism could prove additive if together the FADs intensely activate the pathway of harm. Conversely, 2 such drugs could prove redundant, resulting in no additional risk, if 1 drug alone saturates the pathway. Illuminating and quantifying this potential iatrogenic source of fracture risk could inform changes in prescribing practices aimed at reducing risk and the consequences associated with fractures.^25,26,27,28,29,30,31^

Ideally, the study of risks associated with FAD combinations would occur through randomized clinical trials (RCTs). However, RCTs addressing this question are not readily feasible because of the large number of potential drug combinations and the need for enormous sample sizes to detect rare outcomes. Ethical concerns would be raised as well by trials randomizing people not to receive drugs known to be effective for specific conditions. Additionally, RCTs often exclude multimorbid, older individuals and thus may not reveal the risk of such drug regimens in the populations most likely to receive them.^32,33,34^ Well-conducted, population-representative, observational studies thus represent a crucial opportunity to identify and estimate signals of harm from drug combinations. Such signals, if strong and reproducible, could inform basic science research exploring biological pathways of risk and perhaps effective harm reduction strategies; they could also help justify and prioritize carefully conducted, narrowly focused RCTs.^35,36,37,38^

We used Medicare administrative data to examine hip fracture risk associated with prescription receipt. We first modeled hip fracture risk associated with overall drug count, then with FAD count and count of other drugs (non-FADS) to examine these broad categories separately. We then modeled risk of hip fracture associated with 21 individual FADs and their 210 possible 2-way combinations vs no FADs.

## Methods

### Data and Cohort

We used Medicare Parts A (inpatient) and B (outpatient) administrative data from 2004 to 2014 and Medicare Part D (prescription) records from 2006 to 2014 for a 20% random sample of fee-for-service enrollees. For cohort inclusion, we identified age-eligible beneficiaries with a minimum of 24 months of Parts A and B coverage from 2004 to 2014 plus at least 12 additional months of A, B, and D coverage from 2006 to 2014 (eFigure 1 in the Supplement). Thus, age-eligible individuals were 67 years or older at the time of first observation. The 24 months before the observation period were required to ascertain past morbidities and past fractures. We excluded patients with a fragility fracture (ie, hip, wrist, humerus, and vertebra) apparent in the 24-month preobservation period. This exclusion conservatively created a relatively homogeneous, lower-risk cohort for exploration of FAD risks in the general, geriatric population. Additionally, patients with any of the following diagnoses or services during the study period were excluded: cancer (except nonmelanoma skin cancer), advanced renal disease, vertebral fracture, or hospice. Fracture and disease definitions appear in eTable 2 in the Supplement, and exclusion justification appears in the eAppendix in the Supplement. We followed the Strengthening the Reporting of Observational Studies in Epidemiology (STROBE) reporting guideline.^39^ The Dartmouth Protection of Human Subjects Committee approved this study and granted a waiver of written informed consent because risk was deemed minimal and research involved materials collected for nonresearch purposes.

### Prescription Receipt

From published studies and meta-analyses we identified 21 distinct FAD groups including, antipsychotics, opioids, thiazide diuretics, loop diuretics, glucocorticoids, PPIs, sedative hypnotics, and SSRIs.^1,2,3,4,5,6,7,8,9,10,11,12,40^ The full drug list is provided in eTable 3 in the Supplement. We identified prescription receipt from the Medicare Prescription Drug Event file. For analysis, the data were structured such that, for each drug group, each person-day was assigned as currently exposed or unexposed. Current exposure was assumed to begin on the date of prescription dispensing. We used days supply to set exposure duration. This same approach was applied to other systemic drugs (non-FADs) which were aggregated into 4 current exposure variables (ie, 0, 1, 2, and ≥3 non-FADs) for models. The eAppendix in the Supplement explains FAD and non-FAD ascertainment via National Drug Codes identified by Lexicomp Basic database (Wolters Kluwer) and First Databank (First Databank), respectively. Three types of fracture-protective drugs were also recorded as time varying by person-day and included in each model as currently exposed or not exposed, as follows: (1) estrogen (eg, systemic estrogen, selective estrogen receptor modulators), (2) osteoporosis medications (eg, bisphosphonates, calcitonin, parathyroid hormone, denosumab), and (3) β-blockers.^41,42,43,44,45,46,47,48^ Each intravenous zoledronic acid dose (retrieved from the Medicare Part B Carrier File) was assumed to confer 12 months of bisphosphonate exposure (eAppendix and eTable 3 in the Supplement).

### Outcome

We identified hip fracture diagnosis codes from inpatient claims.^12,49^ Outcome date was hip fracture admission date. We focused on hip fractures because inpatient-treated hip fractures are temporally discrete events with a precise event date.^50^ This permits accurate sequencing of exposure and outcome, assuring we don’t attribute fracture risk to drugs received after the fracture. This is especially important in the study of opioids, which are often used to treat fracture pain (eTable 2 in the Supplement).

### Covariates

We ascertained the following covariates for model inclusion. From the Medicare enrollment files, we obtained age (classified as aged 67-69 years; thereafter in 5-year groups), sex, race/ethnicity, original Medicare entitlement reason, Medicaid eligibility, and Part D Low-Income Subsidy status. We dichotomized the latter 2 variables for each calendar year, assigning the status if present 1 or more months. Long-term care (LTC) status was assigned if 50% or more of prescriptions in a year were dispensed by an LTC pharmacy (retrieved from Part D Pharmacy Characteristics file). From inpatient and outpatient claims, we identified 22 morbidities and assigned patients to each (dichotomous indicator variable) on the first observed diagnosis code at any point in the study. In selecting morbidities for models, we began with the Charlson Comorbidity Index list.^51^ From this list we dropped cohort exclusion conditions (ie, renal failure and cancer) as well as HIV/AIDS because of extremely low prevalence; we then added conditions associated with falls or fractures, eg, seizure disorder, osteoporosis, tobacco use, depression, rheumatologic disease, and alcohol use (eTable 2 in the Supplement). Morbidities and LTC were considered absorptive states, ie, once present, always present.

### Statistical Analysis

Our principal aim was to estimate hip fracture risk associated with current exposure to FAD combinations. Preliminary models estimated risk associated with overall number of drugs.^52,53^ Main models estimated risk associated with FAD count and non-FAD count and, separately, risk associated with individual FADs and 210 possible 2-way FAD combinations. All models were stratified by sex, an anticipated effect modifier.

For time-varying exposure analysis, each person-day was assigned 1 of 233 mutually exclusive FAD exposure categories: 0 FADs, 21 single FADs, 210 possible 2-way FAD combinations, or an aggregate of 3 or more FADs. For statistical reliability, 2-way exposures with less than 100 person-years (PYs) observation in each sex-specific cohort were grouped into a category called FAD pairs with fewer than 100 PYs. We calculated crude fractures per 1000 PYs and percentage of total PYs of observation for each exposure category by sex. Age group–specific crude fracture rates were also calculated.

We applied Cox regression models to counting-process data^54,55^ and estimated drug-associated fracture risk in the 3 following ways: (1) risk associated with current total drug count (FADs and non-FADs), (2) risk associated with current FAD count category and non-FAD count category (1, 2, and ≥3) vs no drugs, and (3) risk associated with 231 single and 2-way exposure indicators plus an indicator of 3 or more concurrent FADs vs no FADs. These models of specific single and 2-way FAD pairs included indicators of non-FAD exposure (ie, 0, 1, 2, or ≥3). All models included the covariates described earlier, ie, age, sociodemographic factors, comorbidities, and 3 indicators for fracture-protective drugs.

In these models, individuals may have contributed to a variety of exposure groups (including no exposure) as the number and type of drugs they received changed over time. The regression coefficient for each exposure indicator was the fracture risk associated with being in that receipt status vs receiving no FADs. Patients were censored at death; disenrollment from fee-for-service Parts A, B, or D; or first hip fracture.

To explore residual confounding, we repeated sex-specific, categorical exposure (ie, 0, 1, 2, or ≥3) models, stratified by age group. We reasoned that drug count may be a proxy for unrecorded disease and disease severity (potential confounders); both commonly increase with age. We expected fracture risk associated with FADs and non-FADs to increase across progressively older age groups if residual confounding were substantial.

Sensitivity analyses included models with no drug exposure indicators; these were intended to explore estimates associated with known risk factors, such as chronic conditions, when detailed drug exposure was not considered (eTable 6 in the Supplement). Separate sensitivity analyses repeated main models but included consideration of competing risk of death using both subdistribution and cause-specific hazards (eAppendix in the Supplement).

Analyses were initiated in November 2018 and completed April 2019 and conducted with SAS version 4 (SAS Institute). Statistical tests were 2-sided. After false discovery rate control using the Benjamini-Hochberg procedures,^56^ risk estimates with *P* < .05 were considered significant.

## Results

### Cohort Characteristics

Our cohort included 2 646 255 beneficiaries with 11 286 768 PYs of observation. Overall, 1 615 613 beneficiaries (61.1%) were women, 2 136 585 (80.7%) were white individuals, and 219 579 (8.3%) were black individuals. Mean (SD) age was 77.2 (7.3) years at study conclusion. Mean (SD) observation time was 4.3 (2.8) years. Over the 9-year study, 726 704 cohort members (27.5%) received Part D LIS, 611 006 (23.1%) were Medicaid eligible, and 211 128 (8.0%) received an LTC assignment (Table 1).

**Table 1.** Characteristics of Cohort, Composed of a 20% Random Sample of Fee-for-Service Medicare Beneficiaries Meeting Inclusion Criteria, 2006-2014

Characteristic	No. (%)		
	Overall	Women	Men
Total			
Beneficiaries	2 646 255 (100)	1 615 613 (61.05)	1 030 642 (38.95)
Observation years	11 286 768 (100)	7 126 266 (63.14)	4 160 502 (36.86)
Individual observation time, y			
Mean (SD)	4.26 (2.78)	4.41 (2.83)	4.04 (2.70)
Median (IQR)	3.28 (2.00-7.00)	4.00 (2.00-7.22)	3.00 (2.00-6.01)
Age, y			
Mean (SD)	77.20 (7.30)	77.85 (7.63)	76.18 (6.61)
Group			
67-69	582 479 (22.01)	331 951 (20.55)	250 528 (24.31)
70-74	781 389 (29.53)	445 446 (27.57)	335 943 (32.60)
75-79	540 033 (20.41)	322 991 (19.99)	217 042 (21.06)
80-84	383 473 (14.49)	246 872 (15.28)	136 601 (13.25)
≥85	396 811 (15.00)	287 663 (17.81)	109 148 (10.59)
Race/ethnicity			
White	2 136 585 (80.74)	1 298 172 (80.35)	838 413 (81.35)
Black or African American	219 579 (8.30)	147 924 (9.16)	71 655 (6.95)
Hispanic	166 843 (6.30)	99 552 (6.16)	67 291 (6.53)
Asian	86 389 (3.26)	50 085 (3.10)	36 304 (3.52)
Other^a^	36 859 (1.39)	19 880 (1.23)	16 979 (1.65)
Original reason for Medicare entitlement			
Disability	248 882 (9.41)	135 989 (8.42)	112 893 (10.95)
Age	2 397 694 (90.61)	1 479 811 (91.59)	917 883 (89.06)
Medicaid eligible^b^	611 006 (23.09)	412 682 (25.54)	198 324 (19.24)
Part D Low-Income Subsidy^b^	726 704 (27.46)	488 780 (30.25)	237 924 (23.09)
Long-term care^c^	211 128 (7.98)	150 721 (9.33)	60 407 (5.86)
Hierarchical Conditions Category score, mean (SD)	0.98 (0.94)	0.97 (0.91)	1.01 (0.99)
Chronic conditions^d^			
Osteoporosis or osteopenia	126 281 (4.77)	116 862 (7.23)	9419 (0.91)
Tobacco use or COPD	357 050 (13.49)	185 073 (11.46)	171 977 (16.69)
Dementia	185 842 (7.02)	127 446 (7.89)	58 396 (5.67)
Obesity	180 431 (6.82)	116 572 (7.22)	63 859 (6.20)
Depression	180 648 (6.83)	133 806 (8.28)	46 842 (4.54)
Serious mental illness	49 592 (1.87)	32 710 (2.02)	16 882 (1.64)
Alcohol use disorder	7841 (0.30)	1999 (0.12)	5842 (0.57)
Diabetes	1 103 209 (41.69)	650 019 (40.23)	453 190 (43.97)
Liver disease	58 335 (2.20)	32 361 (2.00)	25 974 (2.52)
Pancreatic disease	99 952 (3.78)	66 090 (4.09)	33 862 (3.29)
Irritable bowel syndrome	59 679 (2.26)	39 403 (2.44)	20 276 (1.97)
Rheumatologic disease	373 797 (14.13)	270 234 (16.73)	103 563 (10.05)
Spinal cord disease or injury	71 042 (2.68)	42 610 (2.64)	28 432 (2.76)
Serious neurologic disease	435 370 (16.45)	264 824 (16.39)	170 546 (16.55)
Parkinson or Huntington disease	75 637 (2.86)	40 571 (2.51)	35 066 (3.40)
Seizure disorder	137 839 (5.21)	84 025 (5.20)	53 814 (5.22)
Congestive heart failure	735 156 (27.78)	441 880 (27.35)	293 276 (28.46)
Coronary artery disease	596 707 (22.55)	326 111 (20.18)	270 596 (26.26)
Cerebrovascular disease	394 218 (14.90)	240 522 (14.89)	153 696 (14.91)
Peripheral vascular disease	1 007 024 (38.05)	608 263 (37.65)	398 761 (38.69)
Traumatic brain injury	70 450 (2.66)	43 785 (2.71)	26 665 (2.59)
Amputee	17 034 (0.64)	7769 (0.48)	9265 (0.90)
Mortality	256 687 (9.70)	157 845 (9.77)	98 942 (9.60)
Hip Fracture	59 805 (2.26)	47 337 (2.93)	12 368 (1.20)

Abbreviations: COPD, chronic obstructive pulmonary disease; IQR, interquartile range.

^a^ Includes Native Hawaiian or other Pacific Islander, American Indian or Alaskan Native, and no race/ethnicity given (ie, replied do not know or refused to all categories).

^b^ Assigned for the full year to beneficiaries qualifying 1 or more months in that year; a proxy measure of poverty.

^c^ Assigned to patients with 50% or more of prescription fills from a long-term care pharmacy type in a single calendar year.

^d^ Prevalence of conditions are cumulative over the 9-year study period. Health condition assignments are based on 1 or more service-associated diagnosis. Some diagnoses of alcohol use disorder are missing because of data redaction associated with addiction care for 2013 to 2014.

### Exposure and Outcomes

Overall, 2 827 284 PYs (25.1%) involved receipt of 1 FAD; 1 322 296 (11.7%), 2 FADs; and 954 505 (8.5%), 3 or more FADs. Among women, 1 827 305 PYs (25.6%) were exposed to 1 FAD, 912 855 (12.8%) to 2 FADs, and 705 500 (9.9%) to 3 or more FADs; exposure in men was slightly lower (1 FAD, 999 979 PYs [24.0%]; 2 FADs, 409 441 PYs [9.8%]; ≥3 FADs, 249 006 PYs [6.0%]) (Table 2). Over the 9-year period, 1 000 118 women (61.9%) and 533 604 men (51.8%) received 2 FADs on 1 or more days; 640 178 women (39.6%) and 294 181 men (28.5%) received 3 or more concurrent FADs on 1 or more days (eTable 4 in the Supplement). Women received more fracture-protective drugs than men. The most common FAD exposures (alone or in combination with other FADs) were opioids (463 419 PYs of exposure), thiazides (1 447 094 PYs of exposure), prescription PPIs (941 159 PYs of exposure), and SSRIs (763 953 PYs of exposure) (eTable 4 in the Supplement). After uncommon 2-way exposures were grouped as FAD pairs with fewer than 100 PYs, the model for women included 204 exposures (27 pairs grouped), and the model for men included 186 exposures (45 pairs grouped) (eTable 5 in the Supplement).

**Table 2.** Fracture Count, Distribution of FAD and Non-FAD Exposure Intensity, and Crude Hip Fracture Incidence Rate

Outcome	Overall (N = 2 646 255)	Women (n = 1 615 613)	Men (n = 1 030 642)
Total hip fractures, No.	59 703	47 386	12 317
Overall hip fracture rate, fractures per 1000 person-years	5.29	6.65	2.96
Total person-years exposed, No. (%)^a^			
FAD			
0	6 182 683 (54.78)	3 680 607 (51.65)	2 502 076 (60.14)
1	2 827 284 (25.05)	1 827 305 (25.64)	999 979 (24.04)
2	1 322 296 (11.72)	912 855 (12.81)	409 441 (9.84)
≥3	954 506 (8.46)	705 500 (9.90)	249 006 (5.98)
Non-FAD			
0	3 083 494 (27.32)	1 896 251 (26.61)	1 187 243 (28.54)
1	1 840 513 (16.31)	1 227 066 (17.22)	613 447 (14.74)
2	1 997 982 (17.70)	1 305 244 (18.32)	692 737 (16.65)
≥3	4 364 780 (38.67)	2 697 706 (37.85)	1 667 075 (40.07)
Crude hip fracture rate, fractures per 1000 person-years			
FAD			
0	2.57	3.34	1.45
1	5.30	6.47	3.15
2	9.00	10.38	5.95
≥3	17.72	19.55	12.51
Non-FAD			
0	3.50	4.44	2.00
1	5.01	6.16	2.73
2	5.48	6.80	3.01
≥3	6.58	8.36	3.71

Abbreviations: FAD, fracture-associated drug; non-FAD, all other prescription drugs that were not FADs.

^a^ Exposure categories are derived from person-day level data. For FADs, receipt of 21 single drugs (1 FAD) and 210 concurrent drug pairs (2 FADs) are classified for each person and for each day of observation. Person-days with 3 or more concurrent FADs were classified as “≥3 FAD.” The same approach was taken to identify non-FAD exposures (eAppendix in the Supplement).

We observed 59 704 hip fractures, 47 386 (79.4%) among women and 12 317 (20.6%) among men. Crude hip fracture rate was 6.65 per 1000 PYs among women and 2.96 per 1000 PYs among men (Table 2), replicating published rates.^50^

### Fracture Risk Associated With Drug Count

For women, crude fracture rate associated with 0 FADs was 3.34 per 1000 PYs; 1 FAD, 6.47 per 1000 PYs; 2 concurrent FADs, 10.38 per 1000 PYs; and 3 or more FADs, 19.55 per 1000 PYs. Among men, corresponding rates were 1.45 per 1000 PYs, 3.15 per 1000 PYs, 5.95 per 1000 PYs, and 12.51 per 1000 PYs, respectively. Crude fracture rates associated with non-FADs increased in parallel with drug count but less steeply (eg, for women receiving 0 vs ≥3 non-FADs, 4.44/1000 PYs vs 8.36/1000 PYs; for men receiving 0 vs ≥3 non-FADs, 2.00/1000 PYs vs 3.71/1000 PYs) (Table 2). Age-specific, crude fracture rates for FADs and non-FADs are presented in the eAppendix and eFigure 2 in the Supplement.

**Figure 1.**
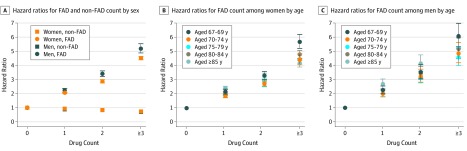
Hip Fracture Hazard Ratios Cox regression analysis results are presented as hazard ratios, with 95% CIs presented as whiskers. Fully adjusted models controlled for age, sociodemographic characteristics, receipt of fracture-protective drugs, and comorbidities. The unit of analysis was person-day. The models revealed risk associated with current receipt of 1 of 21 individual fracture-associated drug (FAD) groups, current receipt of 1 of 210 possible FAD pairs, or any concurrent receipt of 3 or more FADs (eTable 6 in the Supplement). Non-FAD indicates other systemic drugs.

Fully adjusted models exploring total overall drug count and hip fracture risk revealed a hazard ratio (HR) of 1.10 (95% CI, 1.09-1.10; *P* < .001) among women and an HR of 1.10 (95% CI, 1.09-1.11; *P* < .001) among men for each additional drug compared with none (eTable 6 in the Supplement). Fully adjusted models including FAD and non-FAD counts exhibited diverging risks for these broad drug categories. For women, compared with receiving 0 FADs, receiving 1 FAD was associated with an HR of 2.04 (95% CI, 1.99-2.11; *P* < .001); receiving 2 FADs was associated with an HR of 2.86 (95% CI, 2.77-2.95; *P* < .001); and receiving 3 FADs or more was associated with an HR of 4.50 (95% CI, 4.36-4.65; *P* < .001). Comparable non-FAD exposure was associated with an HR of 0.93 (95% CI, 0.90-0.96; *P* < .001), an HR of 0.84 (95% CI, 0.81-0.87; *P* < .001), and an HR of 0.74 (95% CI, 0.72-0.77; *P* < .001), respectively (Figure 1A; eTable 6 in the Supplement). Among men, compared with receiving 0 FADs, receiving 1 FAD was associated with an HR of 2.23 (95% CI, 2.11- 2.36; *P* < .001); receiving 2 FADs was associated with an HR of 3.40 (95% CI, 3.20-3.61; *P* < .001); and receiving 3 or more FADs was associated with an HR of 5.18 (95% CI, 4.87-5.52; *P* < .001). Comparable non-FAD exposure was associated with an HR of 0.90 (95% CI 0.84-0.96; *P* < .001), an HR of 0.84 (95% CI, 0.79-0.89; *P* < .001), and an HR of 0.71 (95% CI, 0.67-0.75; *P* < .001) respectively (Figure 1A; eTable 6 in the Supplement). Age-stratified analyses revealed no consistent association between increasing age group and fracture risk associated with FAD count (Figure 1B and Figure 1C; eTable 6 in the Supplement).

### Fracture Risk Associated With Specific Drug Pairs

In presenting results of models exploring over 200 distinct exposures, we provided all estimates in eTable 7 and eTable 8 in the Supplement but focused our report on exposures contributing relatively substantial population-level risk. We defined substantial population effect combinations as those meeting the 4 following criteria: (1) common (ie, top quintile of exposure PYs), (2) associated with a 2-fold or higher increase in sex-specific, crude, absolute risk, (3) associated with an HR of 3.00 or more, and (4) associated with 50 or more fractures in our cohort.

**Figure 2.**
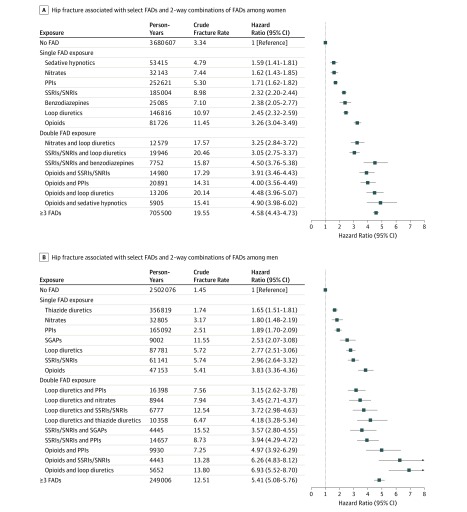
Hip Fracture Risk Associated With Select Fracture-Associated Drugs (FADs) and 2-Way Combinations of FADs Fully adjusted Cox regression analysis results are presented as hazard ratios and 95% CIs. Models were stratified by sex and adjusted for age, sociodemographic characteristics, receipt of fracture-protective drugs, comorbidities, and non-FAD drug receipt. Results displayed were selected for apparent population-level impact; specifically, combinations met all 4 of the following criteria: (1) common (ie, top quintile of person-year exposure); (2) associated with a 2-fold or greater increase in sex-specific, crude, absolute risk; (3) associated with a relative risk of 3.00 or more; and (4) associated with 50 or more fractures in our cohort. Only results that achieved a false-discovery rate *P* < .05 were considered significant. The unit of analysis was person-day. Full models provided in eTable 7 and eTable 8 in the Supplement. PPI indicates proton pump inhibitor; SSRI/SNRI, selective serotonin reuptake inhibitor and selective noradrenergic reuptake inhibitor; SGAP, second-generation antipsychotic.

Among women, 1 FAD group (ie, muscle relaxers) was associated with no increased fracture risk when received alone. Significant risks associated with individual FADs ranged from an HR of 1.54 (95% CI, 1.05-2.24; *P* = .03) for first-generation antipsychotics to an HR of 3.26 (95% CI, 3.04-3.49; *P* < .001) for opioids and an HR of 3.29 (95% CI, 2.89-3.76; *P* < .001) for anti-Parkinson drugs. Among women, only opioids and anti-Parkinson medications were associated with a relative risk above 3.00, while 80 combinations exceeded that threshold; 7 of these 80 met our population-level impact criteria. These included opioids plus sedative hypnotics (HR, 4.90; 95% CI, 3.98-6.02; *P* < .001), opioids plus loop diuretics (HR, 4.48; 95% CI, 3.96-5.07; *P* < .001), opioids plus PPIs (HR 4.00; 95% CI, 3.56-4.49; *P* < .001), SSRIs plus opioids (HR, 3.91; 95% CI, 3.46-4.43; *P* < .001), SSRIs plus benzodiazepines (HR, 4.50; 95% CI, 3.76-5.38; *P* < .001), SSRIs plus loop diuretics (HR 3.05; 95% CI, 2.75-3.37; *P* < .001), and nitrates plus loop diuretics (HR, 3.25; 95% CI, 2.84-3.72; *P* < .001) (Figure 2A; eTable 7 in the Supplement).

Among men, significant risks of single FADs ranged from an HR of 1.51 (95% CI, 1.17-1.95; *P* = .002) for sedative hypnotics to an HR of 3.83 (95% CI, 3.36-4.36; *P* < .001) for opioids and an HR of 4.23 (95% CI 3.57-5.01; *P* < .001) for anti-Parkinson drugs. A total of 9 pairs met our population-level impact criteria; 5 paralleled women’s high-risk pairs (opioids plus loop diuretics, opioids plus PPIs, SSRIs plus opioids, SSRIs plus loop diuretics, and nitrates plus loop diuretics). Among men, the riskiest drug pairs were opioids plus loop diuretics (HR, 6.93; 95% CI, 5.52-8.70; *P* < .001) and opioids plus SSRIs (HR, 6.26; 95% CI, 4.83-8.12; *P* < .001) (Figure 2B; eTable 8 in the Supplement).

For a small number of FAD pairs, receipt of 2 drugs appeared less risky than receipt of a single drug. For example, among women, loop diuretics alone were associated an HR of 2.45 (95% CI, 2.32-2.59; *P* < .001), but loop diuretics plus centrally acting antihypertensives were associated with an HR of 1.82 (95% CI, 1.33-2.49; *P* < .001) (eTable 7 in the Supplement).

## Discussion

Among older Medicare beneficiaries, receipt of FAD combinations is common. Our findings indicated that fracture risk associated with some combinations exceeded individual drug risks. In our study, 20 of 21 examined drug groups were associated with, on average, an approximate 2-fold higher relative risk of hip fracture. Risk increased steeply with the addition of a second FAD (approximately 3-fold higher relative risk) and further with a third (more than 4-fold higher relative risk). Many of the most common, risky pairs included drugs that are relatively contraindicated in our older cohort (eg, sedative hypnotics and benzodiazepines)^57^ or potentially discretionary in some patients (eg, PPIs and opioids). If confirmed, our findings suggest that more cautious prescribing could reduce fracture risk with few or no negative consequences. The clinical implications of risk associated with essential medications (eg, loop diuretics and nitrates) will depend on anticipated net effect (ie, risk plus benefit). That opioids were included in many high-risk pairs reflected their frequent use and associated risk; this finding might reinforce ongoing scrutiny of opioid-prescribing practices.

Our study built on past efforts to estimate risk associated with complex prescription drug combinations. Our results did not support a common approach of defining problematic drug regimens (ie, polypharmacy) based on numeric cutoffs. We found substantial risk associated with just 2 or 3 FADs, counts well below a popular polypharmacy definition of five or more.^58^ Equally important, we found receipt of 3 or more non-FADs associated with a slightly reduced risk of fracture, indicating that more may be better in some cases. These results suggested that a nuanced approach is needed to assess medication count, combinations, and risk. One such nuanced approach is exemplified by the anticholinergic burden framework that explicitly addresses the additive nature of adverse effects resulting from concurrent use of multiple anticholinergic drugs.^59^ Our study similarly considered combinations of drugs with an overlapping adverse effect risk. A total FAD burden conceptual framework may be valuable. Verified estimates could inform risk reduction efforts and risk calculators, such as the Fracture Risk Assessment Tool, which currently considers just 1 prescription group (ie, glucocorticoids).^60,61^

While many studies demonstrate that both type and number of medications are associated with fall and fracture risk, our work expanded understanding by examining drugs associated with fracture through a fall-inducing mechanism and those associated with fracture because of bone weakening.^62,63,64,65,66,67^ We examined these drugs in detail through identification of time-varying exposure to specific FADs and specific FAD pairs, controlling for non-FAD drug receipt. We cannot tell which mechanisms contributed to observed excess risks. We can imagine that combining an opioid with a sedative hypnotic could result in extreme sedation or loss of balance, but the mechanism may be more complex. Like many FADs, opioids have been shown to increase falls and weaken bones.^68,69,70^ Disease-disease interactions or disease-drug interactions could contribute to observed effects. How a drug might mitigate the fracture-promoting effect of another is equally elusive. This has been suggested by others, but such research is nascent.^42,71^ Understanding the mechanisms of harm and protection could inform effective risk reduction strategies.

We found FAD-associated relative risk of hip fracture higher among men than women. This excess relative risk occurred in the context of lower absolute risk for men, but the finding may have important implications because men experience higher mortality after hip fracture than women.^72,73,74^ Sex differences in FAD risks may be an important priority for future research.

### Limitations

Our work has limitations. First, our observational study could suffer from residual confounding if FAD use is related to unobserved behavioral risk factors, unrecorded diseases, or disease severity beyond that controlled for (eg, biological risk factors such as balance and body mass). However, age-group stratified models revealed no increasing risk associated with increasing age group as might be expected given that unobservable confounding is likely more of a problem in older populations; this reduces but does not eliminate residual confounding concerns. Some diseases are inseparable from treatments; for example, Parkinson disease and treatments are both associated with fracture. Our models revealed how the addition of other, potentially discretionary FADs to Parkinson drugs may further increase risks. The estimates of known risk factors, such as age and comorbidities, were abrogated by increasing granularity of drug exposure, as demonstrated in eTable 6 in the Supplement.

Second, our exposure measure was based on prescription fills. The extent to which patients fill but do not take medications, take medications over periods distinct from days supply, or obtain medications through unobserved channels will bias our results. Third, we observed current exposure and classified it dichotomously within drug groups; we did not consider dose, specific drugs within a drug group, cumulative exposure, or recency of FAD initiation. We expect neglecting these factors that influence fracture risk would shift our results toward the null. Future work considering these details will provide deeper understanding of risks because, for example, initiation of drug use has been associated with greatest risk.^10,75^ Fourth, we studied hip fractures; how FAD combinations affect other fragility fractures should be explored. Fifth, Part D coverage for benzodiazepines was limited until 2013; benzodiazepine exposure in early years is likely underestimated.^76^ Sixth, we observed effect estimates for single exposures that aligned with other large-scale studies; thus, our observational design and analytic approach replicated published results.^3,23,75,77,78,79,80,81,82,83,84^ Nonetheless, some drugs we classified as FADs have heterogeneous associations in the literature; some studies find them fracture-associated, others fracture-protective or neutral. We included them to be thorough and to add to the knowledge base. Furthermore, our results may not be generalizable to other populations.

## Conclusions

Our cohort study is hypothesis generating and suggests future research directions. Follow-up studies should explore factors influencing use (and combination use) of potentially avoidable drugs associated with substantial fracture risk. To support clinical decision-making, studies should explore FAD risk in the context of anticipated benefits of essential drugs. Our findings suggested a need to update and refine our conceptual approach to identifying problematic drug regimens, moving past count and appropriateness in favor of nuanced considerations of overlapping risks and benefits. Large data sets and emerging advanced analytic approaches, such as machine learning, may facilitate these efforts as that science and its application to health care data mature.^85^ The exploration of mechanisms through which FAD pairs confer excess risk or risk-mitigating effects could inform efforts aimed at minimizing harm. Such investigations may require basic science approaches considering drug effects at the cellular level and using rodent models involving dual drug exposure and bone-strength measurements. Studies such as ours could inform, justify, and prioritize carefully designed, narrowly targeted human RCTs that quantify comparative risk of treatment alternatives.

## Notes

Supplement. **eAppendix. **Receipt of Multiple, Concurrent Fracture-Associated Drugs and Hip Fracture Risk **eFigure 1.** Cohort Creation Diagram **eFigure 2.** Total and Age-Group Specific Crude Fracture Rates Among Women and Men Exposed to FADs and Non-FADs **eTable 1.** Select Literature Summary, Mechanisms Through Which Drugs Confer Fracture Risk **eTable 2.** *International Classification of Diseases, Ninth Revision* (*ICD*-*9*) Diagnosis Codes Used to Identify Fractures and Morbidities **eTable 3.** Specific Drugs Included in Fracture-Associated and Fracture-Protective Drug Groups **eTable 4.** Descriptive Distribution of Drug Use Over Study Period by Sex **eTable 5. **Number and Category of Fracture-Associated Drug Group Exposures Included in Drug-Specific Models **eTable 6. **Cox Regression Model Results, Associations Between Number of Current Fracture-Associated Drugs and Other Drugs with Hip Fracture **eTable 7. **Cox Proportional Hazard Model Output: Hip Fracture Risk Associated With Fracture-Associated Drugs Among Women **eTable 8. **Cox Proportional Hazard Model Output: Hip Fracture Risk Associated With Fracture-Associated Drugs Among Men
