# Supplementary material for: Association of Receiving Multiple, Concurrent Fracture-Associated Drugs With Hip Fracture Risk
Source: JAMA Netw Open. 2019 Nov 13;2(11):e1915348. doi: 10.1001/jamanetworkopen.2019.15348 (PMC6902800; doi:10.1001/jamanetworkopen.2019.15348)
Supplement: Supplement. — eAppendix. Receipt of Multiple, Concurrent Fracture-Associated Drugs and Hip Fracture Risk eFigure 1. Cohort Creation Diagram eFigure 2. Total and Age-Group Specific Crude Fracture Rates Among Women and Men Exposed to FADs and Non-FADs eTable 1. Select Literature Summary, Mechanisms Through Which Drugs Confer Fracture Risk eTable 2. International Classification of Diseases, Ninth Revision (ICD-9) Diagnosis Codes Used to Identify Fractures and Morbidities eTable 3. Specific Drugs Included in Fracture-Associated and Fracture-Protective Drug Groups eTable 4. Descriptive Distribution of Drug Use Over Study Period by Sex eTable 5. Number and Category of Fracture-Associated Drug Group Exposures Included in Drug-Specific Models eTable 6. Cox Regression Model Results, Associations Between Number of Current Fracture-Associated Drugs and Other Drugs with Hip Fracture eTable 7. Cox Proportional Hazard Model Output: Hip Fracture Risk Associated With Fracture-Associated Drugs Among Women eTable 8. Cox Proportional Hazard Model Output: Hip Fracture Risk Associated With Fracture-Associated Drugs Among Men [file jamanetwopen-2-e1915348-s001.pdf]

Supplementary Online Content

Emeny RT, Chang C-H, Skinner J, et al. Association of receiving multiple, concurrent fracture-associated drugs with hip fracture risk. *JAMA Netw Open*. 2019;2(11):e1915348. doi:10.1001/jamanetworkopen.2019.15348.

eAppendix. Receipt of Multiple, Concurrent Fracture-Associated Drugs and Hip Fracture Risk

eFigure 1. Cohort Creation Diagram

eFigure 2. Total and Age-Group Specific Crude Fracture Rates Among Women and Men Exposed to FADs and Non-FADs

eTable 1. Select Literature Summary, Mechanisms Through Which Drugs Confer Fracture Risk

eTable 2. *International Classification of Diseases, Ninth Revision* (*ICD-9*) Diagnosis Codes Used to Identify Fractures and Morbidities

eTable 3. Specific Drugs Included in Fracture-Associated and Fracture-Protective Drug Groups

eTable 4. Descriptive Distribution of Drug Use Over Study Period by Sex

eTable 5. Number and Category of Fracture-Associated Drug Group Exposures Included in Drug-Specific Models

eTable 6. Cox Regression Model Results, Associations Between Number of Current Fracture-Associated Drugs and Other Drugs with Hip Fracture

eTable 7. Cox Proportional Hazard Model Output: Hip Fracture Risk Associated With Fracture-Associated Drugs Among Women

eTable 8. Cox Proportional Hazard Model Output: Hip Fracture Risk Associated With Fracture-Associated Drugs Among Men

This supplementary material has been provided by the authors to give readers additional information about their work.

© 2019 Emeny RT et al. *JAMA Network Open.*

eAppendix. Receipt of Multiple, Concurrent Fracture-Associated Drugs and Hip Fracture Risk

Cohort Creation:

Because of the two-year Parts A and B coverage requirement, the minimum age was 67 years at the start of Part D drug receipt observation.

Patients with cancer (except non-melanoma of the skin) were conservatively excluded due to risk of pathologic fracture that may be unrelated to drug exposure and imperfectly identified as a cancer-related fracture in claims. Patients with advanced renal disease were also excluded due to risk of fracture related to renal failure and the possibility of receiving drugs during dialysis that would not appear in our claims data. We excluded hospice patients because some medications are included in the hospice per diem payments and thus may not be apparent in our claims data.

We excluded beneficiaries with vertebral fractures at any time during the look-back or observation because the temporal relationship between occurrence and diagnosis of vertebral fractures can vary tremendously. Vertebral fractures are often found incidentally on images obtained for reasons unrelated to fracture. All patients with a past fragility fracture were conservatively excluded from this study because we aimed to study *first* fragility fractures rather than a beneficiary's second or third fragility fracture. Prior fracture is an important fracture risk factor and undoubtedly an effect modifier; this small, high-risk, population must be studied separately by methods appropriate for smaller populations with an event as the key cohort inclusions criterion.[1] Additionally, fragility fracture survivors have a distinctly high fracture risk and their prescriptions may be managed distinctly as a result; their vulnerability to additive adverse drug effects may be different as well. We will study them separately.

Patients censored due to disenrollment in fee-for-service A, B and D plans were permitted to reenter the study as long as they met the inclusion criteria.

Drug Exposure:

For fracture associated drugs, the LexiComp Basic database was used to obtain the active ingredient and product information for each National Drug Code (NDC) apparent in the

© 2019 Emeny RT et al. *JAMA Network Open.*

Part D Event file. Exposure was assumed to begin on the date of prescription dispensing. We used the "days supply" variable to set exposure duration. Overlapping supplies were carried forward (e.g. a fill occurring 80 days after a 90-day fill resulted in a 10-day supply carried forward). For drugs not refilled at the end of the days-supply, discontinuation was assigned after a time elapse of 120% of most recent days-supply, assuming imperfect adherence.

Other drug exposures that were classified as non-fracture associated drug (Non-FAD) exposures represented 552 distinct systemic drug ingredients according to the First Databank (FDB, South San Francisco, CA) Hierarchical Ingredient Code Parent HIC4 Sequence Number (HIC_ROOT); FDB groups ingredients with the same base drug molecule. A 4-level, non-FAD exposure indicator variable was created which flagged 0 or 1 non-FAD exposures on a single day, and aggregated any 2 concurrent non-FAD drug exposures on a single day, and separately any 3 or more concurrent non-FAD drug exposures on a single day. Thus other drug exposures were always included in models as a categorical/numeric indicator for 0, 1, 2 and 3+ concurrent non-FAD exposures. See Technical Appendix Table 1 below for the Ultimate Parent Enhanced Therapeutic Classification Names that contributed to the top 20 most common non-FADs. For example, Cardiovascular Therapy Agents included angiotensin converting enzyme-inhibitors, statins, and angiotensin II receptor blockers, and Analgesic, Anti-inflammatory or Antipyretic drugs included non-narcotic analgesics and NSAID analgesics (COX Non-Specific).

Outcomes:

Hip fractures were selected as the outcome for this study of fracture associated drugs (FADs) because hip fractures are generally treated in the inpatient setting. Thus, the date of fracture occurrence can be reliably ascertained in claims, as can the sequence of prescription receipt and fracture. This issue of sequence of drug receipt and fracture is particularly challenging when assessing other fragility fractures (humerus, wrist, vertebra) as small differences or errors in recorded dates can affect the apparent sequence between medication receipt and fracture occurrence and result in erroneous causal inference. This is especially important for opioids which are often prescribed to treat fracture pain.

© 2019 Emeny RT et al. *JAMA Network Open.*

Analytic Approach:

The study design assured model assumptions were satisfied with regard to non-informative censoring; individuals were censored with the occurrence of death or disenrollment from fee-for-service Medicare Part A, B or D. The probability of a fracture occurring is not related to disenrollment, but may be related to unmeasured factors associated with death.

Risk Adjustment and Stratified, Hierarchical Models. Our empirical analysis addresses two key issues in the identification of fracture risks associated with multiple drug use. First, concerns about unmeasured confounding are addressed by risk-adjustment and stratification by sex. This is further explored in sex-specific age stratified models. Additionally, we perform a sensitivity analysis with a model including only non-drug variables (no drug exposure indicators) to explore the estimates associated with known risk factors when prescription exposure is not considered. Second, we ran hierarchical models using increasingly granular definitions of drug exposures. Our first models include total drug exposure: as a continuous variable and then, separately as a categorical variable representing 0, 1, 2 or 3 or more total concurrent drugs. We then grouped drugs into FAD and non-FAD categories representing 0, 1, 2 or 3-or-more concurrent drugs to determine if combinations of FAD or non-FAD exposures confer greater risk than none, or single exposures. We repeated this categorical approach using FAD and non-FAD designations in age-stratified models. Finally, we assessed approximately 200 specific single and combination exposures in a sex-specific models, applying the Benjamini-Hochberg false-discovery-rate (FDR) control procedure.[2,3] With the very large number of possible drug combinations, we are concerned about falsely rejecting the null for specific interaction effects and therefore control for multiple testing.

Our study has been designed to control for both time varying drug exposures and time varying covariates. We use the counting process survival analysis approach[7,8] to capture exposures with high sensitivity (at a day level) allowing each beneficiary to contribute time to diverse categories of exposure person days (control) in observed follow-up times. Additionally, we accounted for acquisition of health conditions over time that may impact medications received and risk of fracture. Models were stratified on sex, an anticipated effect modifier.

© 2019 Emeny RT et al. *JAMA Network Open.*

Cox Models. In the FAD count exposure analysis, the exposure predictors are binary indicators of the number of FADs taken at time *t*, where *t* ranges over the days the beneficiary meets eligibility criteria. Because patients change status with respect to these variables over follow-up and the time to fracture may be censored, we use the counting process form of the Cox model to estimate the risk of each number of FADs. The model has the form

$$h(t|\boldsymbol{X_i}(t)) = h_0(t)\exp(\beta_1 X_{1i}(t) + \beta_2 X_{2i}(t) + \beta_3 X_{(3+)i}(t) + \boldsymbol{\beta_{cov}^T}\, \boldsymbol{X_{cov,i}}(t))$$

where $X_{ki}(t) = 1$ if patient *i* received *k* FAD at time t and 0 otherwise for $k = 1, 2, \geq 3$ and the regression coefficients β_k represent the effect of a patient receiving k FAD versus 0 FAD at a given time. In addition, $X_{cov}(t)$ is a vector of covariates comprising:

COVStatic = covariates that do not change over observation time, i.e. sex, race, original eligibility

COVAnnual = covariates that are updated annually= age, LIS, Medicaid eligibility

COVAbsorptive = covariates that once in are always in for an individual, i.e. comorbidities, LTC

In the drug and drug-pair specific analysis, for each drug exposure (RX) the time-dependent exposure will equal either a single FAD (single exposure (SE), n=21 FADs), a double exposure of two distinct FADs (double exposure (DE), n=210 combined exposures), or a general three-or-more distinct FAD exposure at time t. The model has the form:

$$\begin{aligned} &h(t|\boldsymbol{X_i}(t)) \\ &= h_0(t)\exp(\beta_1 RXSEFAD_{1i}(t) + \cdots + \beta_{21} RXSEFAD_{21i}(t) + \beta_{22} RXDEFAD_{1i}(t) + \cdots \\ &+ \beta_{231} RXDEFAD_{210i}(t) + \beta_{232} FAD_{(3+)i}(t) \\ &+ \beta_{RXPro} RX_{RXPro,i}(t) + \beta_{1NonFAD} RXNonFAD_{1i}(t) + \beta_{2NonFAD} RXNonFAD_{2i}(t) + \beta_{3+NonFAD} RXNonFAD_{3+i}(t) \\ &+ \boldsymbol{\beta_{cov}^T}\, \boldsymbol{X_{cov,i}}(t)) \end{aligned}$$

where $RXSEFAD_{ki}(t) = 1$ if drug $k = 1, \ldots, 21$ is the only single FAD taken by patient *i* at time *t*, $RXDEFAD_{ki}(t) = 1$ if drug pair $k = 1, \ldots, 210$ corresponds to the only two FADs patient *i* is taking at time *t*, $RXNonFAD_{ki}(t) = 1$, 2, 3+ if drug $k = 1,2,3+$ corresponds to Non-FADs taken by patient *i* at time *t*, and $RX_{RXPro,i}(t) = 1$ if drug(s) taken by patient *i* is an osteoprotective drug.

Competing Risk Analysis. In sensitivity analyses, Cox proportional hazard models were fit in the presence of the potential competing risk of death using the Fine-Gray method which models the cumulative incidence of an event of interest (fracture) given a competing event (death). The resulting subdistribution hazard ratio (SHR) describes the probability of events occurring over time associated with the relative effect of a covariate.[9,10] Sensitivity analyses were additionally run using the cause-specific hazard models which estimate the effect of covariates on the rate of events over time.[9].

Results

Risk adjustment, drug exposure and crude rates. Hierarchical conditions category (HCC) score was calculated to facilitate comparison of our cohort to the general, Medicare population, (national median is 1.0); higher scores indicate more morbidities.[11,12] Hierarchical conditions category scores (mean, SD) were 0.97 (0.91) for women and 1.01 (0.99) for men.
Among women, 27% of person-days were exposed to zero non-FADs, 17% to one non-FAD, 18% to two non-FADs and 38% to three-or-more non-FADs. Exposure among men was similar. See Table 2 in main text.

Crude fracture rates associated with non-FADs were modestly higher than those of women. Receipt of none, or any one, two, or three or more non-FADs was associated with crude fracture rates of 4.44/1000 PY, 6.16/1000 PY, 6.80/1000 PY and 8.38/1000 PY among women, and 2.00/1000 PY, 2.73/1000 PY, 3.01/1000 PY and 3.71/1000 PY for men, respectively (Appendix Figure 2 and Technical Appendix Tables 2a and 2b, respectively for women and men). Person years of exposure to increasing FADs were similar across age strata, and crude rates increased with age and drug use, as expected.

Hierarchical Cox Models. The risk estimates for chronic conditions (but not other risk factors such as age/race) were slightly higher in models that did not include any drug exposure variable (Appendix Table 6, Model A). With increasing granularity of drug definition, these estimates were further abrogated. This is particularly notable when the categorical total drug exposure

variable (Appendix Table 6, Models C1) is separated into FAD versus non-FAD exposures Appendix Table 6 Model C2).

Among results for exclusive single and double FAD exposures (Appendix Tables 7 and Figure 2A and Appendix Table 8 and Figure 2B). For women only one FAD (muscle relaxers) and 44 combinations in women did not achieve False Discovery Rate significant fracture risk. Among men, 5 single FAD exposures (muscle relaxers, tricyclic antidepressants, central acting antihypertensives, 1st generation antihistamines and barbiturates) and 53 double FAD exposures did not achieve False Discovery Rate significance.

Competing Risk Analysis. Cox model results for FAD and non-FAD exposure were slightly higher than results from subdistribution hazard ratios and nearly identical to cause-specific hazard ratio models (Technical Appendix Table 3). Models for single and combination FAD exposures were also run using the subdistribution and cause-specific hazard ratio; results were similar to those obtained from Cox models (data provided upon request).

© 2019 Emeny RT et al. *JAMA Network Open.*

**Technical Appendix Table 1. Ultimate Parent First Databank Enhanced Therapeutic Classification Names for the most commonly received non-FAD groups in a 20% Study Cohort, 2006-2014.**
Cardiovascular Therapy Agents
Analgesic, Anti-inflammatory or Antipyretic
Endocrine
Anti-Infective Agents
Vaccines
Respiratory Therapy Agents
Gastrointestinal Therapy Agents
Electrolyte Balance-Nutritional Products
Genitourinary Therapy

© 2019 Emeny RT et al. *JAMA Network Open.*

Technical Appendix Table 2a. Person Years Exposure and Crude Fracture Rate Associated with Fracture Associated Drugs (FADs) and Non-FADs Across Age Strata in Women

		Fracture Associated Drug (FAD) Exposure				Other Drug (Non-FAD) Exposure				
		FX	PY	Crude Fracture Rate (FX/1000 PY)	% Total PY Exposure		FX	PY	Crude Fracture Rate (FX/1000 PY)	% Total PY Exposure
**Total**		47,387	7,126,267	6.65			47,387	7,126,267	6.65	
n=1,615,613	**0 Drugs**	12,299	3,680,607	3.34	51.65	**0 Drugs**	8,419	1,896,251	4.44	26.61
	**1 Drug**	11,823	1,827,305	6.47	25.64	**1 Drug**	7,553	1,227,066	6.16	17.22
	**2 Drugs**	9,471	912,855	10.38	12.81	**2 Drugs**	8,870	1,305,244	6.80	18.32
	**3+ Drugs**	13,794	705,500	19.55	9.90	**3+ Drugs**	22,545	2,697,706	8.36	37.86
**67-69**		6,422	2,719,708	2.36			6,422	2,719,708	2.36	
n=326,256	**0 Drugs**	1427	1,443,036	0.99	53.06	**0 Drugs**	1030	694846	1.48	25.55
	**1 Drug**	1391	675,794	2.06	24.85	**1 Drug**	1028	517876.8	1.99	19.04
	**2 Drugs**	1192	331,825	3.59	12.20	**2 Drugs**	1074	509339.8	2.11	18.73
	**3+ Drugs**	2412	269053.4	8.96	9.89	**3+ Drugs**	3290	997644.9	3.30	36.68
**70-74**		7,183	1,725,284	4.16			7,183	1,725,284	4.16	
n=436,790	**0 Drugs**	1831	889718.7	2.06	51.57	**0 Drugs**	1250	479871.3	2.60	27.81
	**1 Drug**	1674	450499	3.72	26.11	**1 Drug**	1063	290328	3.66	16.83
	**2 Drugs**	1390	220097.3	6.32	12.76	**2 Drugs**	1276	309557.7	4.12	17.94
	**3+ Drugs**	2288	164969.1	13.87	9.56	**3+ Drugs**	3594	645527.1	5.57	37.42
**75-79**		9,937	1,299,459	7.65			9,937	1,299,459	7.65	
n=322,991	**0 Drugs**	2536	650903.6	3.90	50.09	**0 Drugs**	1716	343245.5	5.00	26.41
	**1 Drug**	2494	348411	7.16	26.81	**1 Drug**	1528	206402.5	7.40	15.88
	**2 Drugs**	2005	172601.6	11.62	13.28	**2 Drugs**	1824	236420.9	7.72	18.19
	**3+ Drugs**	2902	127543.2	22.75	9.82	**3+ Drugs**	4869	513390.4	9.48	39.51
**80-84**		11,282	830,290	13.59			11,282	830,290	13.59	
n=243,570	**0 Drugs**	2944	410550.3	7.17	49.45	**0 Drugs**	1918	216936.8	8.84	26.13
	**1 Drug**	2953	219526.9	13.45	26.44	**1 Drug**	1781	127518.9	13.97	15.36
	**2 Drugs**	2310	114487.1	20.18	13.79	**2 Drugs**	2197	150837.7	14.57	18.17
	**3+ Drugs**	3075	85725.3	35.87	10.32	**3+ Drugs**	5386	334996.2	16.08	40.35
**85+**		12,563	551,526	22.78			12,563	551,526	22.78	
n=287,663	**0 Drugs**	3561	286397.4	12.43	51.93	**0 Drugs**	2505	161351.3	15.53	29.26
	**1 Drug**	3311	133074.3	24.88	24.13	**1 Drug**	2153	84940.01	25.35	15.40
	**2 Drugs**	2574	73844.7	34.86	13.39	**2 Drugs**	2499	99087.56	25.22	17.97
	**3+ Drugs**	3117	58209.47	53.55	10.55	**3+ Drugs**	5406	206147.1	26.22	37.38

© 2019 Emeny RT et al. *JAMA Network Open.*

Technical Appendix Table 2b. Person Years Exposure and Crude Fracture Rate Associated with Fracture Associated Drugs (FADs) and Non-FADs Across Age Strata in Men

		Fracture Associated Drug (FAD) Exposure				Other Drug (Non-FAD) Exposure				
		FX	PY	Crude Fracture Rate (FX/1000 PY)	% Total PY Exposure		FX	PY	Crude Fracture Rate (FX/1000 PY)	% Total PY Exposure
**Total**		12,317	4,160,502	2.96			12,317	4,160,502	2.96	
n=1,030,642	**0 Drugs**	3,616	2,502,076	1.45	60.14	**0 Drugs**	2,376	1,187,243	2.00	28.54
	**1 Drug**	3,150	999,979	3.15	24.04	**1 Drug**	1,676	613,447	2.73	14.74
	**2 Drugs**	2,435	409,441	5.95	9.84	**2 Drugs**	2,087	692,737	3.01	16.65
	**3+ Drugs**	3,116	249,006	12.51	5.98	**3+ Drugs**	6,178	1,667,075	3.71	40.07
**67-69**		2,531	1,878,320	1.35			2,531	1,878,320	1.35	
n=249,638	**0 Drugs**	706	1139329	1.31	60.66	**0 Drugs**	485	491861.7	0.99	26.19
	**1 Drug**	577	444999.6	3.17	23.69	**1 Drug**	354	298599	1.19	15.90
	**2 Drugs**	458	180312.6	6.64	9.60	**2 Drugs**	422	328196.7	1.29	17.47
	**3+ Drugs**	790	113679.1	18.93	6.05	**3+ Drugs**	1270	759662.8	1.67	40.44
**70-74**		2,300	1,090,187	2.11			2,300	1,090,187	2.11	
n=326,887	**0 Drugs**	715	658340.3	0.85	60.39	**0 Drugs**	463	329396.8	1.41	30.21
	**1 Drug**	561	265664.5	1.63	24.37	**1 Drug**	299	154448	1.94	14.17
	**2 Drugs**	438	105151.6	3.27	9.65	**2 Drugs**	367	176504.4	2.08	16.19
	**3+ Drugs**	586	61030.19	7.63	5.60	**3+ Drugs**	1171	429837.4	2.72	39.43
**75-79**		2,639	674,997	3.91			2,639	674,997	3.91	
n=217,042	**0 Drugs**	784	398421.6	1.15	59.03	**0 Drugs**	479	201267.3	2.38	29.82
	**1 Drug**	679	167432.7	2.08	24.80	**1 Drug**	347	91274.39	3.80	13.52
	**2 Drugs**	536	68866.2	4.13	10.20	**2 Drugs**	427	108058.4	3.95	16.01
	**3+ Drugs**	640	40276.53	9.41	5.97	**3+ Drugs**	1386	274,397	5.05	40.65
**80-84**		2,465	345,945	7.13			2,465	345,945	7.13	
n=133,943	**0 Drugs**	707	203721.5	1.97	58.89	**0 Drugs**	464	107294.4	4.32	31.01
	**1 Drug**	683	83304.37	4.17	24.08	**1 Drug**	326	46158.46	7.06	13.34
	**2 Drugs**	501	36745.19	7.33	10.62	**2 Drugs**	420	53938.43	7.79	15.59
	**3+ Drugs**	574	22173.67	14.76	6.41	**3+ Drugs**	1255	138553.5	9.06	40.05
**85+**		2,382	171,053	13.93			2,382	171,053	13.93	
n=109,148	**0 Drugs**	704	102263.9	3.12	59.78	**0 Drugs**	485	57422.4	8.45	33.57
	**1 Drug**	650	38577.73	7.44	22.55	**1 Drug**	350	22967.26	15.24	13.43
	**2 Drugs**	502	18365.61	12.63	10.74	**2 Drugs**	451	26039.19	17.32	15.22
	**3+ Drugs**	526	11845.73	44.40	6.93	**3+ Drugs**	1096	64624.15	16.96	37.78

FAD is fracture associated drug group. Non-FADs are all other prescription drug groups. PY is Person Year. 3+ FAD is a classification of person days with 3 or more concurrent FADs. Frequencies of exposure categories are derived from person-day level data. For FADs, status of receipt of 21 single drugs (1 FAD) and 210 concurrent drug pairs (2 FAD) is classified for each person, for each day of observation. The same approach was taken to identify non-FAD exposures; status of receipt of any 1 non-FAD expsoure (1 Non-FAD), 2 concurrent non-FAD exposures (2 Non-FAD), 3+ Non-FAD is a classification of person days with 3 or more concurrent Non-FADs.

© 2019 Emeny RT et al. *JAMA Network Open.*

Technical Appendix Table 3. Cox Regression Models (Figure 1A) and, for comparison, Competing Risk Models with Sub Distribution or Cause Specific Hazard Ratio Estimates Demonstrating Hip-Fracture Risk Associated with Exposure to prescription FADs and Non-FADs									
	Cox Model Fracture			Competing Risk (SHR)			Competing Risk (CSH)		
	Hazard Ratio (HR)	Lower 95% Confidence Interval	Higher 95% Confidence Interval	Sub Distribution Hazard Ratio (SHR)	Lower 95% Confidence Interval	Higher 95% Confidence Interval	Cause-Specific Hazard Ratio (CHR)	Lower 95% Confidence Interval	Higher 95% Confidence Interval
**Women (Fracture = 47,318; Death n=157,807)**									
**0 FAD**	1.00			1.00			1.00		
**1 FAD**	2.04	1.99	2.11	2.00	1.94	2.06	2.04	1.98	2.11
**2 FAD**	2.86	2.77	2.95	2.76	2.66	2.85	2.86	2.76	2.96
**3+ FAD**	4.50	4.36	4.65	4.17	4.02	4.32	4.50	4.34	4.67
**0 Non-FAD**	1.00			1.00			1.00		
**1 Non-FAD**	0.93	0.90	0.96	0.94	0.91	0.97	0.93	0.90	0.96
**2 Non-FAD**	0.84	0.81	0.87	0.85	0.83	0.88	0.84	0.81	0.87
**3+ Non-FAD**	0.74	0.72	0.77	0.76	0.74	0.78	0.74	0.72	0.77
**Men (Fracture = 12,299; Death = 98,927)**									
**0 FAD**	1.00			1.00			1.00		
**1 FAD**	2.23	2.11	2.36	2.17	2.05	2.30	2.23	2.10	2.36
**2 FAD**	3.40	3.20	3.61	3.22	3.02	3.43	3.39	3.18	3.62
**3+ FAD**	5.18	4.87	5.52	4.60	4.30	4.92	5.17	4.83	5.54
**0 Non-FAD**	1.00			1.00			1.00		
**1 Non-FAD**	0.90	0.84	0.96	0.91	0.85	0.98	0.90	0.84	0.96
**2 Non-FAD**	0.84	0.79	0.89	0.86	0.81	0.92	0.84	0.79	0.89
**3+ Non-FAD**	0.71	0.67	0.75	0.73	0.69	0.78	0.71	0.67	0.75

Total person years of observation = 7,126,266 for women, 4,160,502 for men. For FADs, status of receipt of 21 single drugs (1 FAD) and 210 concurrent drug pairs (2 FAD) is classified for each person, for each day of observation. 3+ FAD is a classification of person days with 3 or more concurrent FADs. The same approach was taken to identify 552 non-FAD exposures; status of receipt of any 1 non-FAD expsoure (1 Non-FAD), 2 concurrent non-FAD exposures (2 Non-FAD), 3+ Non-FAD is a classification of person days with 3 or more concurrent Non-FADs. All models were stratified on sex and adjusted for time varying patient factors; Medicaid eligibility and Medicare Part D low income subsidy are two poverty indicators. Long-term care indicator is defined as 50% or more annual prescription fills dispensed by a long-ter m care pharmacy. The three categories of drugs protective of fracture are: 1. osteoporosis treatments: oral and injected bisphosphonates, calcitonin, denosumab, parathyroid hormone, 2. Estrogens- systemic estrogens, selective estrogen receptor modulators, and 3. beta-blockers. The "Tobacco/Chronic Obstructive Lung Disease" co-morbidity includes patients with Chronic Obstructive Lung Disease diagnosis and/or tobacco use diagnosis.

© 2019 Emeny RT et al. *JAMA Network Open.*

eAppendix References

1. Munson JC, Bynum JP, Bell JE, et al. PAtterns of prescription drug use before and after fragility fracture. JAMA Internal Medicine 2016;176:1531-8.
2. Benjamini Y, Hochberg Y. Controlling the False Discovery Rate: A Practical and Powerful Approach to Multiple Testing. Journal of the Royal Statistical Society Series B (Methodological) 1995;57:289-300.
3. Glickman ME, Rao SR, Schultz MR. False discovery rate control is a recommended alternative to Bonferroni-type adjustments in health studies. J Clin Epidemiol 2014;67:850-7.
4. Avorn J. In defense of pharmacoepidemiology--embracing the yin and yang of drug research. N Engl J Med 2007;357:2219-21.
5. Vandenbroucke JP. When are observational studies as credible as randomised trials? Lancet 2004;363:1728-31.
6. Scott IA, Hilmer SN, Reeve E, et al. Reducing inappropriate polypharmacy: the process of deprescribing. JAMA Intern Med 2015;175:827-34.
7. Hosmer DWL, S.; May, S. Appendix 2: An Introduction to the Counting Process Approach to Survival Analysis. Applied Survival Analysis.
8. Andersen PK, Gill RD. Cox's Regression Model for Counting Processes: A Large Sample Study. Ann Statist 1982;10:1100-20.
9. Austin PC, Fine JP. Practical recommendations for reporting Fine-Gray model analyses for competing risk data. Statistics in Medicine 2017;36:4391-400.
10. Fine JP, Gray RJ. A Proportional Hazards Model for the Subdistribution of a Competing Risk. Journal of the American Statistical Association 1999;94:496-509.
11. Pope GC, Kautter J, Ellis RP, et al. Risk adjustment of Medicare capitation payments using the CMS-HCC model. Health Care Financ Rev 2004;25:119-41.
12. Risk Adjustment. 2019. at https://www.cms.gov/medicare/health-plans/medicareadvtgspecratestats/risk-adjustors.html.)

© 2019 Emeny RT et al. *JAMA Network Open.*

eFigure 1. Cohort Creation Diagram

Cohort inclusion criteria: U.S. ZIP Code, 24 months or more of fee-for-service Medicare Parts A and B enrollment prior to the start of Part D drug receipt observation period, continuous enrollment in fee for service Medicare Parts A, B, and D for at least 12 months between 2006-2014, alive at least 12 months after start of Part D drug use observation.

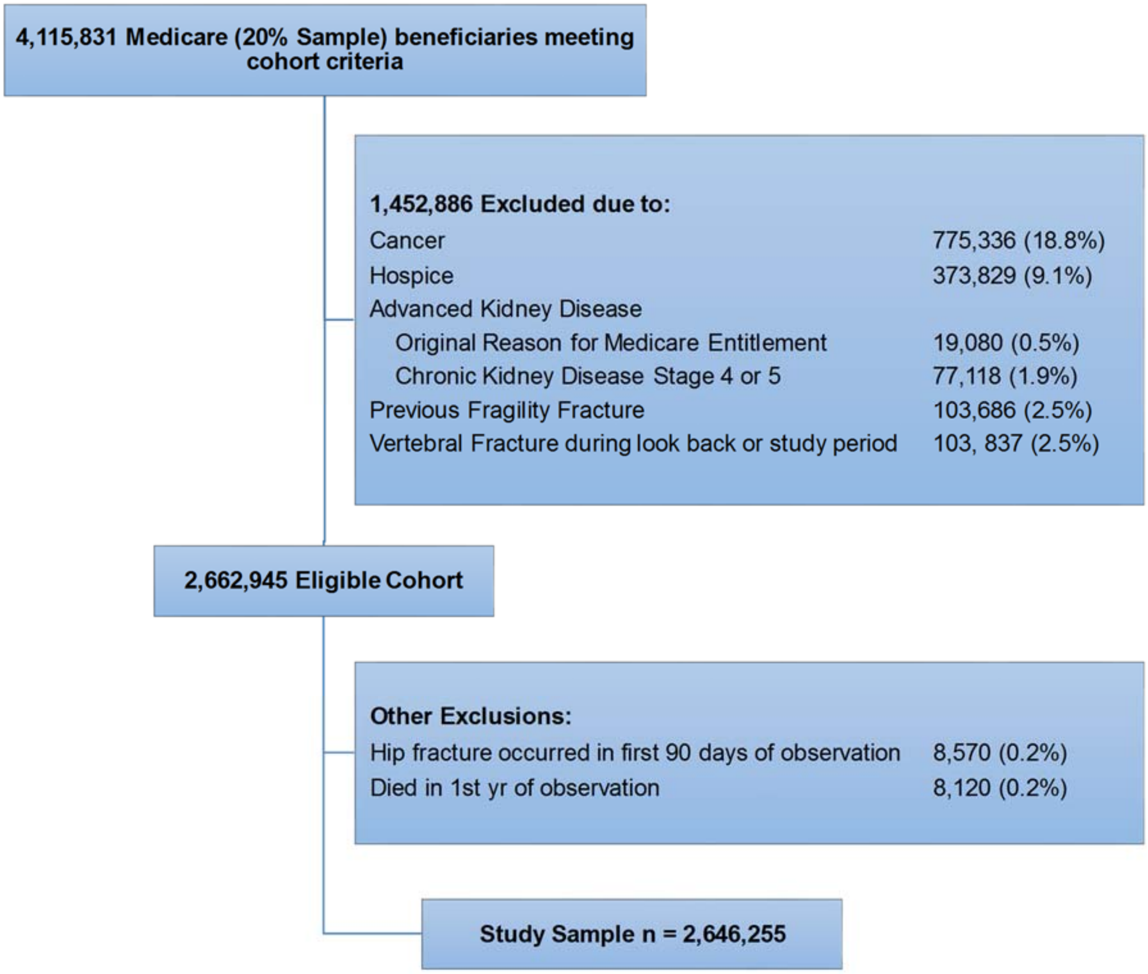

© 2019 Emeny RT et al. *JAMA Network Open.*

eFigure 2. Total and Age-Group Specific Crude Fracture Rates Among Women and Men Exposed to FADs and Non-FADs

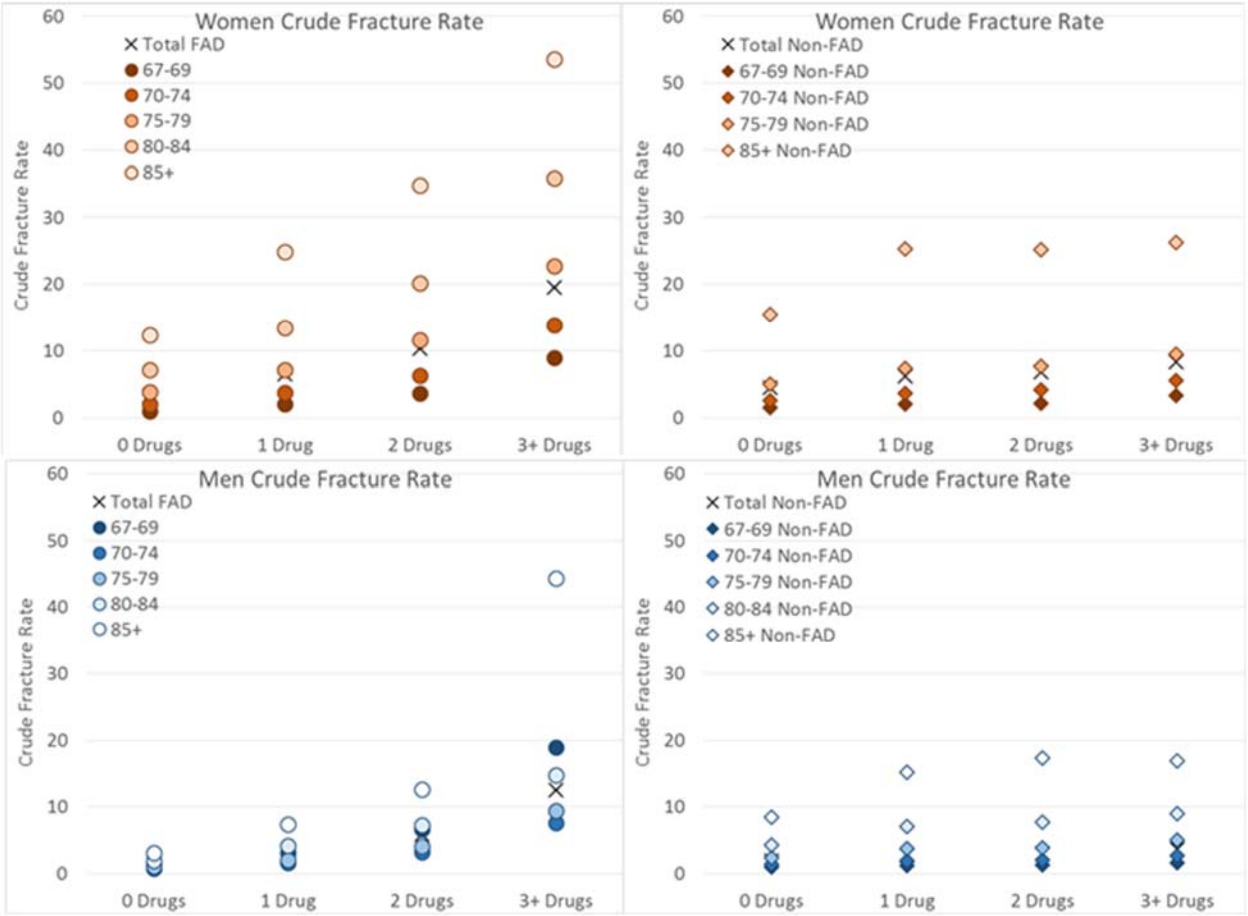

Total is crude rate for women or men exposed to FAD or non-FAD, regardless of age group.

© 2019 Emeny RT et al. *JAMA Network Open.*

eTable 1. Select Literature Summary, Mechanisms Through Which Drugs Confer Fracture Risk

Potential Mechanism of Increased Fracture Risk	Drug Class
Direct skeletal cell effects (on either osteoclasts (bone resorption) or osteoblasts and osteocytes (bone formation)) :	Anticonvulsants [1-10]
· via 25-hydroxylase enzyme inhibition and decreased vitamin D	**Antihistamines (H1 Blockers)[11,12]**
· Folic acid depletion and hyperhomocysteinemia	**Antipsychotics (1st and 2nd generation) [13-17]**
· Decrease serum calcium (increase excretion, decrease absorption, unknown mechanism)	Glucocorticoids[18-23]
· Hypogonadism (aromatase enzyme inhibition, hyperprolactinemia, suppression of hypothalamic, pituitary-gonadal axis)	**Histamine 2 Receptor Antagonists [11,12,24]**
· hypoglycemia	**Loop diuretics[25-28]**
	Nitrates[26,29,30]
	Proton pump inhibitors[24,31-36]
	**Opioids[37,38]**
	**Selective serotonin reuptake inhibitors[39-48]**
	**Thiazide Diuretics[49-51]**
	Thiazolidinediones[52,53]
	**Tricyclic antidepressants [54]**
Increase fall risk	**Antihistamines (H1 Blockers)[55]**
· Sedation/Dizziness/Anticholinergic	AntiParkinson'sAgents[56]
· Orthostatic hypotension	**Antipsychotics (1st and 2nd generation) [57-61]**
	Barbiturates[62]
	Benzodiazepines[58,61,63,64]
	Centrally-acting antihypertensives[65]
	**Histamine 2 Receptor Antagonists [24]**
	**Loop diuretics[66]**
	Muscle Relaxers[67-69]
	Non-Benzodiazepine Sedative Hypnotics[57,58,61]
	**Opioids[37,38,70]**
	**Selective serotonin reuptake inhibitors[57,58,71]**
	**Thiazide Diuretics[49-51]**
	**Tricyclic antidepressants [57,58,61,71]**
Increase fall risk<br>· Decrease muscle mass	Glucocortcoids[19]

Bold font indicates drug groups that have been associated with both risk of falls and bone weakening.

© 2019 Emeny RT et al. *JAMA Network Open.*

Literature

1. Arora E, Singh H, Gupta YK. Impact of antiepileptic drugs on bone health: Need for monitoring, treatment, and prevention strategies. Journal of Family Medicine and Primary Care 2016;5:248-53.
2. Hosseinpour F, Ellfolk M, Norlin M, Wikvall K. Phenobarbital suppresses vitamin D3 25-hydroxylase expression: a potential new mechanism for drug-induced osteomalacia. Biochem Biophys Res Commun 2007;357:603-7.
3. Teagarden DL, Meador KJ, Loring DW. Low Vitamin D Levels Are Common in Patients with Epilepsy. Epilepsy research 2014;108:1352-6.
4. Khanna S, Pillai KK, Vohora D. Insights into liaison between antiepileptic drugs and bone. Drug Discov Today 2009;14:428-35.
5. Pack AM. Treatment of epilepsy to optimize bone health. Curr Treat Options Neurol 2011;13:346-54.
6. Pack AM, Morrell MJ. Adverse effects of antiepileptic drugs on bone structure: epidemiology, mechanisms and therapeutic implications. CNS Drugs 2001;15:633-42.
7. Fan H-C, Lee H-S, Chang K-P, et al. The Impact of Anti-Epileptic Drugs on Growth and Bone Metabolism. International Journal of Molecular Sciences 2016;17:1242.
8. Feldkamp J, Becker A, Witte OW, Scharff D, Scherbaum WA. Long-term anticonvulsant therapy leads to low bone mineral density--evidence for direct drug effects of phenytoin and carbamazepine on human osteoblast-like cells. Exp Clin Endocrinol Diabetes 2000;108:37-43.
9. Jacobsen NW, Halling-Sorensen B, Birkved FK. Inhibition of human aromatase complex (CYP19) by antiepileptic drugs. Toxicol In Vitro 2008;22:146-53.
10. Weinstein RS, Bryce GF, Sappington LJ, King DW, Gallagher BB. Decreased serum ionized calcium and normal vitamin D metabolite levels with anticonvulsant drug treatment. J Clin Endocrinol Metab 1984;58:1003-9.
11. Ezzat BA, Abbass MM. The ability of H1 or H2 receptor antagonists or their combination in counteracting the glucocorticoid-induced alveolar bone loss in rats. J Oral Pathol Med 2014;43:148-56.
12. Kinjo M, Setoguchi S, Solomon DH. Anti-histamine Therapy and Bone Mineral Density: Analysis in a Population-Based U.S. Sample. The American journal of medicine 2008;121:1085-91.
13. Meaney AM, Smith S, Howes OD, O'Brien M, Murray RM, O'Keane V. Effects of long-term prolactin-raising antipsychotic medication on bone mineral density in patients with schizophrenia. Br J Psychiatry 2004;184:503-8.
14. Chen C-Y, Lane H-Y, Lin C-H. Effects of Antipsychotics on Bone Mineral Density in Patients with Schizophrenia: Gender Differences. Clinical Psychopharmacology and Neuroscience 2016;14:238-49.
15. Naidoo U, Goff DC, Klibanski A. Hyperprolactinemia and bone mineral density: the potential impact of antipsychotic agents. Psychoneuroendocrinology 2003;28 Suppl 2:97-108.
16. Tseng PT, Chen YW, Yeh PY, Tu KY, Cheng YS, Wu CK. Bone Mineral Density in Schizophrenia: An Update of Current Meta-Analysis and Literature Review Under Guideline of PRISMA. Medicine 2015;94:e1967.
17. Costa JL, Smith G, Watson M, et al. The atypical anti-psychotic clozapine decreases bone mass in rats in vivo. Schizophr Res 2011;126:291-7.
18. O'Brien CA, Jia D, Plotkin LI, et al. Glucocorticoids act directly on osteoblasts and osteocytes to induce their apoptosis and reduce bone formation and strength. Endocrinology 2004;145:1835-41.
19. Güler-Yüksel M, Hoes JN, Bultink IEM, Lems WF. Glucocorticoids, Inflammation and Bone. Calcified Tissue International 2018.
20. Yao W, Cheng Z, Busse C, Pham A, Nakamura MC, Lane NE. Glucocorticoid Excess in Mice Results in Early Activation of Osteoclastogenesis and Adipogenesis and Prolonged Suppression of Osteogenesis: A Longitudinal Study of Gene Expression in Bone Tissue From Glucocorticoid-Treated Mice. Arthritis and rheumatism 2008;58:1674-86.
21. Chee C, Sellahewa L, Pappachan JM. Inhaled Corticosteroids and Bone Health. The Open Respiratory Medicine Journal 2014;8:85-92.
22. Weinstein RS. Glucocorticoid-induced osteoporosis and osteonecrosis. Endocrinol Metab Clin North Am 2012;41:595-611.
23. Weinstein RS, Jilka RL, Parfitt AM, Manolagas SC. Inhibition of osteoblastogenesis and promotion of apoptosis of osteoblasts and osteocytes by glucocorticoids. Potential mechanisms of their deleterious effects on bone. J Clin Invest 1998;102:274-82.
24. Eom C-S, Park SM, Myung S-K, Yun JM, Ahn J-S. Use of acid-suppressive drugs and risk of fracture: a meta-analysis of observational studies. Ann Fam Med 2011;9:257-67.
25. Rejnmark L, Vestergaard P, Heickendorff L, Andreasen F, Mosekilde L. Loop diuretics increase bone turnover and decrease BMD in osteopenic postmenopausal women: results from a randomized controlled study with bumetanide. J Bone Miner Res 2006;21:163-70.
26. Ghosh M, Majumdar SR. Antihypertensive medications, bone mineral density, and fractures: a review of old cardiac drugs that provides new insights into osteoporosis. Endocrine 2014;46:397-405.
27. Lim LS, Fink HA, Blackwell T, Taylor BC, Ensrud KE. Loop diuretic use and rates of hip bone loss and risk of falls and fractures in older women. J Am Geriatr Soc 2009;57:855-62.
28. Rejnmark L, Vestergaard P, Mosekilde L. Fracture risk in patients treated with loop diuretics. J Intern Med 2006;259:117-24.
29. Jamal SA, Reid LS, Hamilton CJ. The effects of organic nitrates on osteoporosis: a systematic review. Osteoporos Int 2013;24:763-70.
30. Golchin N, Hohensee C, LaCroix A, Gray SL. Nitrate Medications, Fractures, and Change in Bone Mineral Density in Postmenopausal Women: Results from the Women's Health Initiative. Journal of Bone and Mineral Research 2016;31:1760-6.
31. O'Connell MB, Madden DM, Murray AM, Heaney RP, Kerzner LJ. Effects of proton pump inhibitors on calcium carbonate absorption in women: a randomized crossover trial. Am J Med 2005;118:778-81.
32. Eom C-S, Park SM, Myung S-K, Yun JM, Ahn J-S. Use of Acid-Suppressive Drugs and Risk of Fracture: A Meta-analysis of Observational Studies. The Annals of Family Medicine 2011;9:257-67.
33. Corley DA, Kubo A, Zhao W, Quesenberry C. Proton Pump Inhibitors and Histamine-2 Receptor Antagonists are Associated with Hip Fractures among At-Risk Patients. Gastroenterology 2010;139:93-101.
34. Chonan O, Takahashi R, Yasui H, Watanuki M. Effect of L-lactic acid on calcium absorption in rats fed omeprazole. J Nutr Sci Vitaminol (Tokyo) 1998;44:473-81.
35. Aasarød KM, Stunes AK, Mosti MP, et al. Effects of the Histamine 1 Receptor Antagonist Cetirizine on the Osteoporotic Phenotype in H+/K+ATPase Beta Subunit KO Mice. Journal of Cellular Biochemistry 2016;117:2089-96.
36. Jo Y, Park E, Ahn SB, et al. A Proton Pump Inhibitor's Effect on Bone Metabolism Mediated by Osteoclast Action in Old Age: A Prospective Randomized Study. Gut and Liver 2015;9:607-14.
37. Daniell HW. Opioid osteoporosis. Arch Intern Med 2004;164:338; author reply
38. Daniell HW. DHEAS deficiency during consumption of sustained-action prescribed opioids: evidence for opioid-induced inhibition of adrenal androgen production. J Pain 2006;7:901-7.
39. Warden SJ, Robling AG, Sanders MS, Bliziotes MM, Turner CH. Inhibition of the serotonin (5-hydroxytryptamine) transporter reduces bone accrual during growth. Endocrinology 2005;146:685-93.
40. Warden SJ, Robling AG, Haney EM, Turner CH, Bliziotes MM. The emerging role of serotonin (5-hydroxytryptamine) in the skeleton and its mediation of the skeletal effects of low-density lipoprotein receptor-related protein 5 (LRP5). Bone 2010;46:4-12.
41. Bliziotes M, Eshleman A, Burt-Pichat B, et al. Serotonin transporter and receptor expression in osteocytic MLO-Y4 cells. Bone 2006;39:1313-21.
42. Bliziotes M. Update in serotonin and bone. J Clin Endocrinol Metab 2010;95:4124-32.
43. Battaglino R, Fu J, Spate U, et al. Serotonin regulates osteoclast differentiation through its transporter. J Bone Miner Res 2004;19:1420-31.
44. Haney EM, Chan BK, Diem SJ, et al. Association of low bone mineral density with selective serotonin reuptake inhibitor use by older men. Arch Intern Med 2007;167:1246-51.
45. Haney EM, Warden SJ. Skeletal effects of serotonin (5-hydroxytryptamine) transporter inhibition: evidence from clinical studies. J Musculoskelet Neuronal Interact 2008;8:133-45.
46. Haney EM, Warden SJ, Bliziotes MM. Effects of selective serotonin reuptake inhibitors on bone health in adults: time for recommendations about screening, prevention and management? Bone 2010;46:13-7.
47. Rosen CJ. Serotonin rising--the bone, brain, bowel connection. N Engl J Med 2009;360:957-9.
48. Jacobsen NW, Hansen CH, Nellemann C, Styrishave B, Halling-Sorensen B. Effects of selective serotonin reuptake inhibitors on three sex steroids in two versions of the aromatase enzyme inhibition assay and in the H295R cell assay. Toxicol In Vitro 2015;29:1729-35.
49. Aung K, Htay T. Thiazide diuretics and the risk of hip fracture. Cochrane Db Syst Rev 2011.
50. Xiao F, Qu X, Zhai Z, et al. Association between loop diuretic use and fracture risk. Osteoporosis International 2015;26:775-84.
51. Xiao X, Xu Y, Wu Q. Thiazide diuretic usage and risk of fracture: a meta-analysis of cohort studies. Osteoporosis International 2018.
52. Grey A, Bolland M, Gamble G, et al. The peroxisome proliferator-activated receptor-gamma agonist rosiglitazone decreases bone formation and bone mineral density in healthy postmenopausal women: a randomized, controlled trial. J Clin Endocrinol Metab 2007;92:1305-10.
53. Grey A, Bolland MJ, Wattie D, Horne A, Gamble G, Reid IR. The antiresorptive effects of a single dose of zoledronate persist for two years: a randomized, placebo-controlled trial in osteopenic postmenopausal women. J Clin Endocrinol Metab 2009;94:538-44.
54. Li X, Sun W, Li J, et al. Clomipramine causes osteoporosis by promoting osteoclastogenesis via E3 ligase Itch, which is prevented by Zoledronic acid. Scientific Reports 2017;7:41358.
55. Du Buske LM. Clinical comparison of histamine H1 receptor antagonist drugs. Journal of Allergy and Clinical Immunology 1996;98:S307-S18.
56. Whitney CM. Medications for Parkinson's disease. Neurologist 2007;13:387-8.
57. Ray WA, Griffin MR, Schaffner W, Baugh DK, Melton LJ, 3rd. Psychotropic drug use and the risk of hip fracture. N Engl J Med 1987;316:363-9.
58. Cumming RG. Epidemiology of medication-related falls and fractures in the elderly. Drugs Aging 1998;12:43-53.
59. Hartikainen S, Loennroos E, Louhivuori K. Medication as a Risk Factor for Falls: Critical Systematic Review. J Gerontol a-Biol 2007;62:1172-81.
60. Nourian Z, Mow T, Muftic D, et al. Orthostatic hypotensive effect of antipsychotic drugs in Wistar rats by in vivo and in vitro studies of alpha1-adrenoceptor function. Psychopharmacology (Berl) 2008;199:15-27.
61. Woolcott JC, Richardson KJ, Wiens MO, et al. Meta-analysis of the impact of 9 medication classes on falls in elderly persons. Arch Intern Med 2009;169:1952-60.
62. Grisso JA, Kelsey JL, Strom BL, et al. Risk factors for falls as a cause of hip fracture in women. The Northeast Hip Fracture Study Group. N Engl J Med 1991;324:1326-31.
63. Donnelly K, Bracchi R, Hewitt J, Routledge PA, Carter B. Benzodiazepines, Z-drugs and the risk of hip fracture: A systematic review and meta-analysis. PLoS ONE 2017;12:e0174730.
64. Cumming RG, Le Couteur DG. Benzodiazepines and risk of hip fractures in older people: a review of the evidence. CNS Drugs 2003;17:825-37.
65. Mathias CJ. Orthostatic hypotension: causes, mechanisms, and influencing factors. Neurology 1995;45:S6-11.
66. Berry SD, Mittleman MA, Zhang Y, et al. New loop diuretic prescriptions may be an acute risk factor for falls in the nursing home. Pharmacoepidemiology and Drug Safety 2012;21:560-3.
67. Alvarez CA, Mortensen EM, Makris UE, et al. Association of skeletal muscle relaxers and antihistamines on mortality, hospitalizations, and emergency department visits in elderly patients: a nationwide retrospective cohort study. BMC Geriatrics 2015;15:2.
68. Golden AG, Ma Q, Nair V, Florez HJ, Roos BA. Risk for fractures with centrally acting muscle relaxants: an analysis of a national Medicare Advantage claims database. Ann Pharmacother 2010;44:1369-75.
69. Witenko C, Moorman-Li R, Motycka C, et al. Considerations for the Appropriate Use of Skeletal Muscle Relaxants for the Management Of Acute Low Back Pain. Pharmacy and Therapeutics 2014;39:427-35.
70. Guo ZC, Wills P, Viitanen M, Fastbom J, Winblad B. Cognitive Impairment, Drug Use, and the Risk of Hip Fracture in Persons over 75 Years Old: A Community-based Prospective Study. American Journal of Epidemiology 1998;148:887-92.
71. Thapa PB, Gideon P, Cost TW, Milam AB, Ray WA. Antidepressants and the risk of falls among nursing home residents. N Engl J Med 1998;339:875-82.

© 2019 Emeny RT et al. *JAMA Network Open.*

eTable 2. *International Classification of Diseases, Ninth Revision* (ICD-9) Diagnosis Codes Used to Identify Fractures and Morbidities

**Hip Fracture**	82000 82001 82002 82003 82009 82010 82011 82012 82013 82019 82020 82021 82022 82030 82031 82032 8208 8209 82100 82101 82110 82111
**Distal Radius/Wrist Fragility Fracture**	81340 81341 81342 81343 81344 81345 81346 81347 81350 81351 81352 81353 81354 81400 81410
**Humerus Fragility Fracture**	81200 81201 81202 81203 81209 81210 81211 81212 81213 81219
**Vertebral Fragility Fracture**	805.0x 805.2x 805.4x 805.6x 805.8x
**Pathologic Fracture**	73310 73311 73312 73313 73314 73315 73316 73319
**Chronic Kidney Disease Stages 4/5 or Dialysis Status**	5854 5855 5856 5880 40301 40311 40391 40402 40403 40412 40413 40492 40493 99656 99668 99673 V4511 V4512 V560 V561 V562 V5631 V5632 V568 5854 5855 5856 5880 40301 40311 40391 40402 40403 40412 40413 40492 40493 99656 99668 99673 V4511 V4512 V560 V561 V562 V5631 V5632 V568
**Cancer (except non-melanoma of skin)**	1400-1729 1740-20935 20970-20979 2350-2399 V1000-V1091 1960-1192
**Tobacco /Chronic Obstructive Pumonary Disease**	415.0 416.8 416.9 491.0 492.0 494.0 496.0 305.1 V15.82 989.84
**Depression**	296.2 296.21 296.22 296.3 296.23 296.25 296.26 296.30 296.31 296.32 296.33 296.35 296.36 300.4 301.10 301.11 301.12 301.13 309.1 311
**Serious Mental Illness**	295.00 295.01 295.02 295.03 295.04 295.05 295.10 295.11 295.12 295.13 295.14 295.15 295.20 295.21 295.22 295.23 295.24 295.25 295.30 295.31 295.32 295.33,295.34 295.35 295.40 295.41 295.42 295.43 295.44 295.45 295.50 295.51 295.52 295.53 295.54 295.55 295.60 295.61 295.62 295.63 295.64 295.65 295.70 295.71 295.72 295.73 295.74 295.75 295.80 295.81 295.82 295.83 295.84 295.85 295.90 295.91 295.92 295.93 295.94 295.95<br>296.00 296.01 296.02 296.03 296.04 296.05 296.06 296.10 296.11 296.12 296.13 296.14 296.15 296.16 296.24 296.34 296.40 296.41 296.42 296.43 296.44 296.45 296.46 296.50 296.51 296.52 296.53 296.54 296.55 296.56 296.60 296.61 296.62 296.63 296.64 296.65 296.66 296.7 296.80 296.81 296.82 296.89 296.90 296.99 297.0 297.1 297.2 297.3 297.8 297.9 2980 2981 2982 2983 2984 2988 2989
**Alcohol Abuse**	V113.x V791.x 291.0-291.9 303.0-303.9** Please see note below on data redaction
**Obesity Overweight**	27801 27803 V8535 V8536 V8537 V8538 V8539 V854 V8541 V8542 V8543 V8544 V8545 27800 V853 V8530 V8531 V8532 V8533 V8534 27802 V852 V8521 V8522 V8523 V8524 V8525
**Osteoporosis/osteopenia**	73300-73309
**Dementia**	046 046.11 046.19 046.2 046.3 046.71 046.72 046.79 046.8 046.9 290 290.1 290.11 290.12 290.13 290.2 290.21 290.3 290.4 290.41 290.42 290.43 290.8 290.9 294 294.1 294.11 294.2 294.21 294.8 294.9 330 330.1 330.2 330.3 330.8 330.9 331 331.7 331.11 331.19 331.2 331.6 331.81 331.82 331.89 331.9
**Diabetes**	'24900','24901','24910','24911','24920','24921','24930','24931','24940','24941','24950','24951','24960','24961','24970','24971','24980','24981','24990','24991','25000','25001','25002','25003','25010','25011','25012','25013','25020','25021','25022','25023','25030','25031','25032','25033','25040','25041','25042','25043','25050','25051','25052','25053','25060','25061','25062','25063','25070','25071','25072','25073','25080','25081','25082','25083','25090','25091','25092','25093','36201','36203','36204','36205','36206','36207','36641','V5867',
**Liver Disease**	'5550','5551','5552','5559','5560','5561','5562','5563','5564','5565','5566','5568','5569',
**Pancreatic Diseaase**	'0402','0723','2514','2515','2518','2519','5770','5771','5772','5778','5779','5790','5791','5792','5793','5794','5798','5799',
**Inflammatory Bowel Disease**	'5550','5551','5552','5559','5560','5561','5562','5563','5564','5565','5566','5568','5569',
**Rheumatologic Disease**	'0993','1361','4460','4461','44620','44621','44629','4463','4464','4465','4466','4467','6960','7100','7101','7102','7103','7104','7105','7108','7109','71110','71111','71112','71113','71114','71115','71116','71117','71118','71119','71120','71121','71122','71123','71124','71125','71126','71127','71128','71129','7140','7141','7142','71430','71431','71432','71433','7144','71481','71489','7149','7200','7201','7202','72081','72089','7209','725',
**Spinal Cord disease or injury**	'0522','05314','05474','32302','32342','32352','32363','32372','32382','3340','3341','3342','3343','3344','3348','3349','3350','33510','33511','33519','33520','33521','33522','33523','33524','33529','3358','3359','3360','3361','3362','3363','3368','3369','34120','34121','34122','3430','3432','34400','34401','34402','34403','34404','34409','3441','34460','34461','34481','7400','7401','7402','74100','74101','74102','74103','74190','74191','74192','74193','7420','7421','7422','7423','7424','74251','74253','74259','7428','7429','78072','80600','80601','80602','80603','80604','80605','80606','80607','80608','80609','80610','80611','80612','80613','80614','80615','80616','80617','80618','80619','80620','80621','80622','80623','80624','80625','80626','80627','80628','80629','80630','80631','80632','80633','80634','80635','80636','80637','80638','80639','8064','8065','80660','80661','80662','80669','80670','80671','80672','80679','8068','8069','9072','95200','95201','95202','95203','95204','95205','95206','95207','95208','95209','95210','95211','95212','95213','95214','95215','95216','95217','95218','95219','9522','9523','9524','9528','9529',
**Serious Neurologic Disease**	'33700','33701','33709','3371','33720','33721','33722','33729','3373','3379','340','3410','3411','3418','3419','3560','3561','3562','3563','3564','3568','3569','3570','3571','3572','3573','3574','3575','3576','3577','35781','35782','35789','3579','35800','35801','3581','3582','35830','35831','35839','3588','3589','3590','3591','35921','35922','35923','35924','35929','3593','3594','3595','3596','35971','35979','35981','35989','3599',
**Parkinson's and Huntington's Diseases**	3320','3321','3330','3334', '3320','3321','3330','3334',
**Seizure Disorders and Convulsions**	'34500','34501','34510','34511','3452','3453','34540','34541','34550','34551','34560','34561','34570','34571','34580','34581','34590','34591','78031','78032','78033','78039',
**Congestive heart failure**	'39891','40201','40211','40291','40401','40411','40491','4150','4160','4161','4168','4169','4170','4171','4178','4179','4250','4251','42511','42518','4252','4253','4254','4255','4257','4258','4259','4280','4281','42820','42821','42822','42823','42830','42831','42832','42833','42840','42841','42842','42843','4289','4290','4291',
**Coronary Artery Disease**	'41000','41001','41002','41010','41011','41012','41020','41021','41022','41030','41031','41032','41040','41041','41042','41050','41051','41052','41060','41061','41062','41070','41071','41072','41080','41081','41082','41090','41091','41092','4110','4111','41181','41189','412','4130','4131','4139','4295','4296',
**Cerebrovascular Disease**	'09487','34200','34201','34202','34210','34211','34212','34280','34281','34282','34290','34291','34292','3431','3434','3442','34430','34431','34432','34440','34441','34442','3445','34481','34489','3449','430','431','4320','4321','4329','43301','43311','43321','43331','43381','43391','43401','43411','43491','436','43820','43821','43822','43830','43831','43832','43840','43841','43842','43850','43851','43852','43853','99702',

© 2019 Emeny RT et al. *JAMA Network Open.*

eTable 2 continued

**Peripheral Vascular Disease**	'0400','41511','41512','41513','41519','4162','4400','4401','44020','44021','44022','44023','44024','44029','44030','44031','44032','4404','44100','44101','44102','44103','4411','4412','4413','4414','4415','4416','4417','4419','4420','4421','4422','4423','44281','44282','44283','44284','44289','4429','4431','44321','44322','44323','44324','44329','44381','44382','44389','4439','4440','44401','44409','4441','44421','44422','44481','44489','4449','44501','44502','44581','44589','4470','4471','4472','4473','4474','4475','4476','44770','44771','44772','44773','4478','4479','4480','449','45111','45119','45181','45183','4530','4532','4533','45340','45341','45342','45350','45351','45352','45372','45374','45375','45376','45377','4538','45382','45384','45385','45386','45387','4540','4542','45911','45913','45931','45933','5570','5571','5579','59381','7854',
**Traumatic Brain Injury**	'80000','80001','80002','80003','80004','80005','80006','80009','80010','80011','80012','80013','80014','80015','80016','80019','80020','80021','80022','80023','80024','80025','80026','80029','80030','80031','80032','80033','80034','80035','80036','80039','80040','80041','80042','80043','80044','80045','80046','80049','80050','80051','80052','80053','80054','80055','80056','80059','80060','80061','80062','80063','80064','80065','80066','80069','80070','80071','80072','80073','80074','80075','80076','80079','80080','80081','80082','80083','80084','80085','80086','80089','80090','80091','80092','80093','80094','80095','80096','80099','80100','80101','80102','80103','80104','80105','80106','80109','80110','80111','80112','80113','80114','80115','80116','80119','80120','80121','80122','80123','80124','80125','80126','80129','80130','80131','80132','80133','80134','80135','80136','80139','80140','80141','80142','80143','80144','80145','80146','80149','80150','80151','80152','80153','80154','80155','80156','80159','80160','80161','80162','80163','80164','80165','80166','80169','80170','80171','80172','80173','80174','80175','80176','80179','80180','80181','80182','80183','80184','80185','80186','80189','80190','80191','80192','80193','80194','80195','80196','80199','80220','80221','80222','80223','80224','80225','80226','80227','80228','80229','80230','80231','80232','80233','80234','80235','80236','80237','80238','80239','8024','8025','8026','8027','8028','8029','80300','80301','80302','80303','80304','80305','80306','80309','80310','80311','80312','80313','80314','80315','80316','80319','80320','80321','80322','80323','80324','80325','80326','80329','80330','80331','80332','80333','80334','80335','80336','80339','80340','80341','80342','80343','80344','80345','80346','80349','80350','80351','80352','80353','80354','80355','80356','80359','80360','80361','80362','80363','80364','80365','80366','80369','80370','80371','80372','80373','80374','80375','80376','80379','80380','80381','80382','80383','80384','80385','80386','80389','80390','80391','80392','80393','80394','80395','80396','80399','80400','80401','80402','80403','80404','80405','80406','80409','80410','80411','80412','80413','80414','80415','80416','80419','80420','80421','80422','80423','80424','80425','80426','80429','80430','80431','80432','80433','80434','80435','80436','80439','80440','80441','80442','80443','80444','80445','80446','80449','80450','80451','80452','80453','80454','80455','80456','80459','80460','80461','80462','80463','80464','80465','80466','80469','80470','80471','80472','80473','80474','80475','80476','80479','80480','80481','80482','80483','80484','80485','80486','80489','80490','80491','80492','80493','80494','80495','80496','80499','8502','8503','8504','85100','85101','85102','85103','85104','85105','85106','85109','85110','85111','85112','85113','85114','85115','85116','85119','85120','85121','85122','85123','85124','85125','85126','85129','85130','85131','85132','85133','85134','85135','85136','85139','85140','85141','85142','85143','85144','85145','85146','85149','85150','85151','85152','85153','85154','85155','85156','85159','85160','85161','85162','85163','85164','85165','85166','85169','85170','85171','85172','85173','85174','85175','85176','85179','85180','85181','85182','85183','85184','85185','85186','85189','85190','85191','85192','85193','85194','85195','85196','85199','85200','85201','85202','85203','85204','85205','85206','85209','85210','85211','85212','85213','85214','85215','85216','85219','85220','85221','85222','85223','85224','85225','85226','85229','85230','85231','85232','85233','85234','85235','85236','85239','85240','85241','85242','85243','85244','85245','85246','85249','85250','85251','85252','85253','85254','85255','85256','85259','85300','85301','85302','85303','85304','85305','85306','85309','85310','85311','85312','85313','85314','85315','85316','85319','85400','85401','85402','85403','85404','85405','85406','85409','85410','85411','85412','85413','85414','85415','85416','85419','9050','9070',
**Amputee**	'3536','9059','99760','99761','99762','99769','V4970','V4971','V4972','V4973','V4974','V4975','V4976','V4977','V521',

Some diagnoses of alcohol abuse will be missing due to redaction of data data associated with addiction care for the years (2013-2014). See https://www.resdac.org/resconnect/articles/203

eTable 3. Specific Drugs Included in Fracture-Associated and Fracture-Protective Drug Groups

**Fracture Associated**
**Inhaled Steroids**
beclomethasone
budesonide
dexamethasone
flunisolide
fluticasone
mometasone
triamcinolone
ciclesonide
**Oral Steroids**
betamethasone
budesonide
cortisone
dexamethasone
fludrocortisone
hydrocortisone
methylprednisolone
prednisolone
prednisone
triamcinolone
**Proton Pump Inhibitors**
dexlansoprazole
esomeprazole
lansoprazole
omeprazole
pantoprazole
rabeprazole
**H2 Antagonists/ Histamine 2 Receptor Antagonists**
cimetidine
famotidine
nizatidine
ranitidine
ranitidine bismuth citrate
**SSRI/SNRI Selective Serotonin and Selective Noradrenergic Reuptake Inhibitors**
citalopram
desvenlafaxine
escitalopram
FLUoxetine
fluvoxaMINE
PARoxetine
sertraline
venlafaxine
vortioxetine
levomilnacipran
milnacipran
DULoxetine
**Tricyclic Antidepressants**
amitriptyline
amoxapine
clomipramine
desipramine
imipramine
nortriptyline
protriptyline
trimipramine
doxepin
**Thiazolidinediones**
pioglitazone
rosiglitazone

© 2019 Emeny RT et al. *JAMA Network Open.*

eTable 3 continued

**Anticonvulsants**
felbamate
tiagabine
topiramate
vigabatrin
valproate
carbamazepine
eslicarbazepine
ethosuximide
ethotoin
ezogabine
fosphenytoin
gabapentin
lacosamide
lamotrigine
levetiracetam
mephenytoin
methsuximide
oxcarbazepine
perampanel
phensuximide
phenytoin
pregabalin
rufinamide
zonisamide
**Benzodiazepines**
alprazolam
chlordiazepoxide
clobazam
clonazepam
clorazepate
diazepam
estazolam
flurazepam
halazepam
lorazepam
midazolam
oxazepam
prazepam
quazepam
temazepam
triazolam
**Barbiturates**
butabarbital
butalbital
mephobarbital
pentobarbital
phenobarbital
secobarbital
primidone

© 2019 Emeny RT et al. *JAMA Network Open.*

eTable 3 continued

Opioids
alfentanil
butorphanol
codeine
dezocine
dihydrocodeine
fentanyl
hydrocodone
hydromorphone
levorphanol
meperidine
methadone
morphine
morphine liposomal
nalbuphine
opium
oxycodone
oxymorphone
paregoric
pentazocine
propoxyphene
remifentanil
sufentanil
tapentadol
tramadol
naloxone
buprenorphine
**Muscle relaxers**
meprobamate
methocarbamol
carisoprodol
**1st Generation Antihistamines/ H1 Receptor Antagonists**
azatadine
bromodiphenhydramine
brompheniramine
carbinoxamine
chlorcyclizine
chlorpheniramine
clemastine
cyclizine
cyproheptadine
dexbrompheniramine
dexchlorpheniramine
dimenhydrinate
diphenhydramine
doxylamine
hydroxyzine
meclizine
pheniramine
promethazine
pyrilamine
triprolidine
triethylperazine

© 2019 Emeny RT et al. *JAMA Network Open.*

eTable 3 continued

**Anti- Parkinson's Disease**
amantadine
apomorphine
bromocriptine
cabergoline
carbidopa
entacapone
levodopa
pergolide
pramipexole
rasagiline
ropinirole
rotigotine
selegiline
tolcapone
dihydroergocryptine
benztropine
biperiden
orphenadrine
procyclidine
trihexyphenidyl
profenamine
**Centrally-Acting Antihypertensives**
clonidine
guanabenz
guanfacine
methyldopa
tizanidine
**1st-Generation Antipsychotics (FGAP)**
chlorpromazine
droperidol
fluphenazine
haloperidol
loxapine
mesoridazine
methotrimeprazine
molindone
perphenazine
pimozide
prochlorperazine
thioridazine
thiothixene
trifluoperazine
triflupromazine
**2nd-Generation Antipsychotics (SGAP)**
aripiprazole
asenapine
clozapine
iloperidone
lurasidone
olanzapine
paliperidone
quetiapine
risperidone
ziprasidone
brexpiprazole
cariprazine
pimavanserin

© 2019 Emeny RT et al. *JAMA Network Open.*

eTable 3 continued

Nitrates* (do not include sublingual tablets, oral and transdermal only)
erythrityl tetranitrate
pentaerythritol
nitroglycerin
isosorbide dinitrate
isosorbide mononitrate
**Loop Diuretics**
bumetanide
furosemide
torsemide
ethacrynic acid
**Thiazide and Thiazide-like Diuretics**
bendroflumethiazide
chlorothiazide
chlorthalidone
hydrochlorothiazide
indapamide
methyclothiazide
metolazone
polythiazide
benzthiazide
**Sedative Hypnotics**
acetylcarbromal
busPIRone
chloral hydrate
chlormezanone
doxepin
doxylamine
ethchlorvynol
hydrOXYzine
melatonin
meprobamate
paraldehyde
pyrilamine
ramelteon
sodium oxybate
tasimelteon
dichloralphenazone
suvorexant
eszopiclone
zaleplon
zolpidem
zopiclone

© 2019 Emeny RT et al. *JAMA Network Open.*

eTable 3 continued

**Fracture Protective Drugs**
**Systemic Estrogens**
conjugated estrogens
esterified estrogens
estradiol
estradiol
estradiol
estradiol
estropipate
estrone
ethinyl estradiol
ethinyl estradiol
synthetic conjugated estrogens, A
synthetic conjugated estrogens, B
estriol
**Bisphosphonates**
alendronate
etidronate
ibandronate
risedronate
tiludronate
zoledronate
zoledronate
zoledronate
zoledronate
ibandronate
etidronate
pamidronate
**Calcitonin**
calcitonin
calcitonin
calcitonin, human
**Monoclonal Antibiody (RANK L)**
denosumab
**Parathyroid Hormone (PTH)**
teriparatide
**Selective Estrogen Receptor Modulators**
raloxifene
lasofoxifene
ospemifene
bazedoxifene
tamoxifen
**Beta Blockers**
acebutolol
atenolol
betaxolol
bisoprolol
carteolol
carvedilol
labetalol
metoprolol
nadolol
nebivolol
penbutolol
pindolol
propranolol
timolol
All fracture associated drugs were identified use National Drug Code variable in Medicare Part D Drug Event file and the Multum LexiComp Basic database (Lexicomp). Denver: Cerner Multum. Zoledronic Acid receipt by infusion was also ascertained from Carrier file using Healthcare Common Procedure Coding System codes: j3487, j3488, j3489, Q2051, J1740, J1436, J2430

© 2019 Emeny RT et al. *JAMA Network Open.*

eTable 4. Descriptive Distribution of Drug Use Over Study Period by Sex

Cohort total = 2,646,255 beneficiaries 1,615,613 women, 1,030,642 men, total observation years = 11,286,768; 63.14% women, 36.86% men

Table 4a: Women						
	Cohort Drug Receipt			Individual Level Drug Receipt in Person Years		
	Percent with Any Exposure	Total Time Exposed in Person Years	% of Observed Time Exposed	Mean	Standard Deviation	Median
**Fracture Associated Drugs (alone or incombination with other FAD use)**						
**Thiazide Diuretics**	40.49	1,447,094	20.32	2.21	2.15	1.28
**Proton Pump Inhibitors**	36.83	941,159	13.22	1.58	1.84	0.89
**SSRI/SNRI**	27.76	763,953	10.73	1.70	1.93	1.00
**Loop Diuretics**	22.37	522,112	7.33	1.44	1.78	0.69
**Opioids**	54.99	463,419	6.51	0.52	1.13	0.08
**Anticonvulsants**	18.32	334,594	4.70	1.13	1.60	0.39
**Sedative Hypnotics**	16.94	247,517	3.48	0.90	1.42	0.25
**H2 Receptor Antagonists**	11.20	174,796	2.46	0.97	1.40	0.30
**Nitrates**	10.73	170,486	2.39	0.98	1.65	0.19
**Inhaled Glucocorticoids**	9.58	154,467	2.17	1.00	1.39	0.39
**Oral Glucocorticoids**	33.92	146,070	2.05	0.27	0.74	0.04
**Tricyclic Antidepressants**	6.79	145,666	2.05	1.33	1.84	0.39
**Benzodiazepine**	17.58	129,405	1.82	0.46	0.63	0.20
**2nd Generation Antispychotics**	6.04	128,523	1.81	1.32	1.69	0.59
**Antiparkinson Agents**	5.32	115,705	1.63	1.35	1.80	0.49
**Central Acting Antihypertensives**	6.89	115,664	1.62	1.04	1.62	0.26
**Thiazolidinediones**	4.13	109,814	1.54	1.65	1.70	1.01
**1st Generation Antihistamines**	17.60	56,849	0.80	0.20	0.54	0.05
**Muscle Relaxers**	3.74	22,009	0.31	0.36	0.83	0.10
**1st Generation Antipsychotics**	3.12	20,411	0.29	0.41	1.09	0.03
**Barbiturates**	1.41	18,367	0.26	0.81	1.46	0.13
**Top 20 Most Common Combinations**						
**PPI & Thiazide Diuretics**	13.66	273,718	3.84	1.24	1.54	0.60
**PPI & SSRI/SNRI**	11.58	221,779	3.11	1.19	1.50	0.58
**SSRI/SNRI & Thiazide Diuretics**	9.57	198,221	2.78	1.28	1.58	0.64
PPI & Opioids	16.57	147,759	2.08	0.55	1.04	0.11
**PPI & Loop Diuretics**	8.97	145,621	2.05	1.00	1.36	0.40
**Thiazide Diuretics & Opioids**	17.57	128,398	1.80	0.45	0.93	0.09
SSRI/SNRI & Opioids	12.99	125,785	1.77	0.60	1.11	0.11
Opioids & Loop Diuretics	6.62	112,616	1.58	1.05	1.42	0.42
**PPI & Anticonvulsants**	7.21	102,539	1.44	0.88	1.26	0.30
**Anticonvulsants & SSRI/SNRI**	6.10	96,376	1.35	0.98	1.37	0.36
Opioids & Loop Diuretics	10.24	91,040	1.28	0.55	1.00	0.12
**Anticonvulsants & Thiazide Diuretics**	6.27	86,716	1.22	0.86	1.26	0.30
**Anticonvulsants & Opioids**	9.10	85,358	1.20	0.58	1.05	0.13
**SSRI/SNRI & Sedative Hypnotics**	5.40	74,537	1.05	0.85	1.27	0.30
**PPI & Sedative Hypnotics**	5.90	72,010	1.01	0.76	1.16	0.24
**Thiazide Diuretics & Loop Diuretics**	7.02	69,002	0.97	0.61	0.99	0.22
**Sedative Hypnotics & Thiazide Diuretics**	5.31	66,484	0.93	0.78	1.21	0.23
**Anticonvulsants & Loop Diuretics**	4.52	62,889	0.88	0.86	1.26	0.30
**SSRI/SNRI & SGAP**	3.33	61,350	0.86	1.14	1.50	0.50
Nitrates & Loop Diuretics	3.79	54,180	0.76	0.89	1.38	0.22
**0 FAD**	100					
**1 FAD**	84.96					
**2 FAD**	61.90					
**3+ FAD**	39.62					
**Non-Fracture Associated Drugs**						
**0 Non-FAD**	66.46					
**1 Non-FAD**	51.52					
**2 Non-FAD**	59.51					
**3+ Non-FAD**	69.16					
**Drugs protective of fracture**						
**Beta Blockers**	50.24	1,575,275	22.11	1.94	2.03	1.11
**Osteoporosis medications**	25.92	559,704	7.85	1.34	1.56	0.69
**Estrogen**	18.00	369,450	5.18	1.27	1.62	0.58

© 2019 Emeny RT et al. *JAMA Network Open.*

eTable 4 continued

Table 4b: Men						
	Cohort Drug Receipt			Individual Level Drug Receipt in Person Years		
	Percent with Any Exposure	Total Time Exposed in Person Years	% of Observed Time Exposed	Mean	Standard Deviation	Median
**Fracture Associated Drugs (not exclusive to other concurrent FAD use)**						
**Thiazide Diuretics**	31.02	617,566	14.85	1.93	1.98	1.13
**Proton Pump Inhibitors**	29.86	433,643	10.43	1.41	1.71	0.73
**Loop Diuretics**	18.59	246,196	5.92	1.28	1.62	0.59
**SSRI/SNRI**	15.73	227,489	5.47	1.40	1.71	0.75
**Opioids**	50.11	184,621	4.44	0.36	0.92	0.05
**Anticonvulsants**	14.08	152,255	3.66	1.05	1.52	0.36
**Nitrates**	13.55	110,253	2.65	0.79	1.44	0.16
**Sedative Hypnotics**	12.69	106,411	2.56	0.81	1.30	0.21
**Thiazolidinediones**	5.11	87,790	2.11	1.67	1.69	1.06
**H2 Receptor Antagonists**	8.19	82,587	1.99	0.98	1.42	0.30
**Inhaled Glucocorticoids**	8.25	81,502	1.96	0.96	1.33	0.39
**Oral Glucocorticoids**	29.30	72,475	1.74	0.24	0.66	0.04
**Antiparkinson Agents**	4.96	67,235	1.62	1.32	1.72	0.55
**2nd Generation Antispychotics**	4.43	53,465	1.29	1.17	1.58	0.49
**Benzodiazepine**	11.22	49,972	1.20	0.43	0.63	0.18
**Central Acting Antihypertensives**	4.38	42,186	1.02	0.93	1.48	0.22
**Tricyclic Antidepressants**	3.64	39,298	0.95	1.05	1.57	0.30
**1st Generation Antihistamines**	10.52	18,498	0.45	0.17	0.48	0.04
**Barbiturates**	1.11	11,530	0.28	1.01	1.52	0.30
**Muscle Relaxers**	2.83	9,003	0.22	0.31	0.74	0.10
**1st Generation Antipsychotics**	2.00	8,677	0.21	0.42	1.09	0.04
**Top 20 Most Common Combinations (not exclusive to other concurrent FAD use)**						
**PPI & Thiazide Diuretics**	8.36	95,544	2.30	1.11	1.42	0.52
PPI & SSRI/SNRI	5.67	58,322	1.40	1.00	1.33	0.42
**PPI & Loop Diuretics**	6.15	53,634	1.29	0.85	1.20	0.30
PPI & Opioids	11.44	47,514	1.14	0.40	0.87	0.07
**SSRI/SNRI & Thiazide Diuretics**	4.02	43,313	1.04	1.05	1.37	0.47
**Thiazide Diuretics & Opioids**	11.64	39,073	0.94	0.33	0.77	0.05
**PPI & Anticonvulsants**	4.58	36,900	0.89	0.78	1.15	0.30
SSRI/SNRI & Loop Diuretics	3.62	32,960	0.79	0.88	1.24	0.33
SSRI/SNRI & Opioids	6.36	30,711	0.74	0.47	0.94	0.09
**Anticonvulsants & Opioids**	6.28	30,446	0.73	0.47	0.92	0.10
**Anticonvulsants & Thiazide Diuretics**	3.69	30,350	0.73	0.80	1.19	0.30
Opioids & Loop Diuretics	7.30	29,580	0.71	0.39	0.81	0.08
**Anticonvulsants & SSRI/SNRI**	3.22	29,400	0.71	0.89	1.29	0.30
Thiazide Diuretics & Loop Diuretics	4.75	27,869	0.67	0.57	0.92	0.21
Nitrates & Loop Diuretics	3.64	27,451	0.66	0.73	1.20	0.18
**PPI & Nitrates**	4.13	27,228	0.66	0.64	1.13	0.15
**PPI & Sedative Hypnotics**	3.83	27,098	0.65	0.69	1.08	0.21
**Anticonvulsants & Loop Diuretics**	3.10	25,203	0.61	0.79	1.18	0.28
**TZD & Thiazide Diuretics**	1.75	23,445	0.56	1.30	1.44	0.84
SSRI/SNRI & SGAP	2.12	22,036	0.53	1.01	1.40	0.41
**0 FAD**	100					
**1 FAD**	80.35					
**2 FAD**	51.77					
**3+ FAD**	28.54					
**Non-Fracture Associated Drugs**						
**0 Non-FAD**	64.79					
**1 Non-FAD**	43.29					
**2 Non-FAD**	52.03					
**3+ Non-FAD**	68.62					
**Drugs protective of fracture**						
**Beta Blockers**	42.45	912,897	21.94	2.09	2.09	1.18
**Osteoporosis medications**	3.13	39,563	0.95	1.23	1.49	0.59
**Estrogen**	0.10	743	0.02	0.71	1.07	0.28

Tables present distribution of use of the 21 fracture associated drugs (FADs) of interest, Non-FADs, and three categories of drugs protective of fracture including: 1. Osteoporosis treatments: bisphosphonates, calcitonin, denosumab and parathyroid hormone 2. Estrogens: systemic estrogens and selective estrogen receptor modulators, and 3. Beta-blockers. SSRI/SNRI =combined drug group of selective serotonin reuptake inhibitors and selective norepinephrine reuptake inhibitors. Sedative Hypnotics include non-benzodiazepine drugs only. For FADs, status of receipt of 21 single drugs (1 FAD) and 210 concurrent drug pairs (2 FAD) is classified for each person, for each day of observation. 3+ FAD is a classification of person days with 3 or more concurrent FADs. The same approach was taken to identify 552 non-FAD exposures; status of receipt of any 1 non-FAD expsoure (1 Non-FAD), 2 concurrent non-FAD exposures (2 Non-FAD), 3+ Non-FAD is a classification of person days with 3 or more concurrent Non-FADs. Double exposures with population-relevant risk are italicized.

© 2019 Emeny RT et al. *JAMA Network Open.*

eTable 5. Number and Category of Fracture-Associated Drug Group Exposures Included in Drug-Specific Models

	Women	Men
Single Fracture Associated Drug Groups Possible	21	21
Single Fracture Associated Drug Groups Excluded	0	0
**Single Fracture Associated Drug Groups Included in Models**	**21**	**21**
Double Fracture Associated Drug Groups Possible	210	210
Double Fracture Associated Drug Groups Excluded (due to <100 PY exposure time)	27	45
**Double Fracture Associated Drug Groups Included in Models**	**183**	**165**
**Total Specific Fracture Associated Drug Group Exposures Estimated in Model**	**204**	**186**
Aggregated other exposure (double exposures pairs with < 100PY observation)	1	1
Indicator for Any Three-or-more Fracture Associated Drug Group Exposure Days	1	1
**Total Fracture Associated Drug Group Exposure Indicators Included in Models**	206	188
For statistical stability, within each sex-specific cohort, all exposure pairs for which we had less than 100 total person years of observation, were aggregated into an "FAD pairs with < 100 PY" indicator variable.		

© 2019 Emeny RT et al. *JAMA Network Open.*

eTable 6. Cox Regression Model Results, Associations Between Number of Current Fracture-Associated Drugs and Other Drugs with Hip Fracture

From 20% random sample of Medicare beneficiaries meeting inclusion criteria, stratified by sex. Hip fracture counts: women, 47 386; men, 12 317. Person-years of observation: women, 7 126 266; men: 4 160 502.

Women

		Model A= Sensitivity analysis excluding any drug exposure variable				Model B= Continuous exposure variable for total drug use.				Model C1=Categorized exposure indicator variables for total drug use (1,2,3+ drugs versus none).				Model C2 (Figure 1A)= Exposure indicator variables for concurrent use of fracture associated drugs (FAD 1,2,3+ versus none) as well as all other drug use (Non-FAD 1,2,3+ versus none).			
Parameter		Ratio (HR)	Confidence	Confidence	p-value	(HR)	Confidence	Confidence	p-value	(HR)	Confidence	Confidence	p-value	(HR)	Interval	Confidence Interval	p-value
Age (reference 67-69)																	
Age 70-74		1.30	1.24	1.35	<.0000001	1.29	1.24	1.35	<.0000001	1.30	1.25	1.36	<.0000001	1.24	1.19	1.29	<.0000001
Age 75-79		2.17	2.09	2.25	<.0000001	2.16	2.08	2.25	<.0000001	2.17	2.09	2.25	<.0000001	2.09	2.01	2.18	<.0000001
Age 80-84		3.34	3.22	3.46	<.0000001	3.33	3.21	3.46	<.0000001	3.33	3.20	3.45	<.0000001	3.25	3.12	3.37	<.0000001
Age 85+		5.32	5.12	5.52	<.0000001	5.35	5.16	5.56	<.0000001	5.29	5.09	5.49	<.0000001	5.11	4.92	5.31	<.0000001
Race/Ethnicity (reference white)																	
Black		0.48	0.46	0.50	<.0000001	0.50	0.48	0.53	<.0000001	0.49	0.47	0.51	<.0000001	0.51	0.49	0.53	<.0000001
Hispanic		0.75	0.71	0.78	<.0000001	0.79	0.75	0.83	<.0000001	0.77	0.73	0.80	<.0000001	0.80	0.77	0.84	<.0000001
Asian		0.67	0.63	0.72	<.0000001	0.72	0.67	0.77	<.0000001	0.70	0.65	0.74	<.0000001	0.77	0.72	0.82	<.0000001
Other		1.01	0.93	1.10	0.8291614	1.05	0.96	1.15	0.2534495	1.03	0.95	1.13	0.4756557	1.07	0.98	1.17	0.1237977
Low Income Subsidy		1.08	1.04	1.12	0.0000680	1.04	1.00	1.08	0.0542511	1.06	1.02	1.11	0.0017187	1.02	0.98	1.06	0.2956492
Original Medicare eligibility (%)																	
Disability		1.42	0.79	2.57	0.2444908	1.45	0.80	2.62	0.2171435	1.42	0.79	2.57	0.2411746	1.27	0.71	2.30	0.4236478
Aged		1.11	0.61	2.01	0.7285464	1.19	0.66	2.15	0.5693099	1.13	0.63	2.05	0.6820753	1.08	0.60	1.95	0.8085012
Long term care		1.41	1.38	1.45	<.0000001	1.40	1.37	1.44	<.0000001	1.41	1.38	1.44	<.0000001	1.34	1.30	1.37	<.0000001
Dual eligible		0.95	0.91	0.99	0.0091583	0.93	0.89	0.97	0.0003144	0.95	0.91	0.99	0.0071514	0.89	0.85	0.92	<.0000001
Chronic Conditions																	
Osteoporosis/Osteopenia		3.31	3.24	3.39	<.0000001	3.30	3.23	3.38	<.0000001	3.29	3.22	3.37	<.0000001	3.30	3.23	3.38	<.0000001
Dementia		3.58	3.49	3.67	<.0000001	3.59	3.50	3.68	<.0000001	3.56	3.47	3.65	<.0000001	3.50	3.41	3.59	<.0000001
Obesity		1.06	1.02	1.11	0.0016393	1.00	0.97	1.04	0.8705696	1.05	1.01	1.09	0.0190190	1.00	0.96	1.04	0.9774711
Depression		1.50	1.46	1.54	<.0000001	1.38	1.34	1.41	<.0000001	1.45	1.41	1.49	<.0000001	1.24	1.20	1.27	<.0000001
Serious Mental Illness		1.32	1.27	1.38	<.0000001	1.30	1.24	1.35	<.0000001	1.32	1.26	1.37	<.0000001	1.22	1.17	1.27	<.0000001
Alcohol Abuse		1.88	1.61	2.19	<.0000001	1.97	1.69	2.30	<.0000001	1.91	1.64	2.22	<.0000001	1.94	1.67	2.26	<.0000001
Tobacco/Chronic Lung Disease		2.23	2.17	2.29	<.0000001	2.15	2.10	2.21	<.0000001	2.20	2.14	2.25	<.0000001	2.19	2.14	2.25	<.0000001
Diabetes		0.96	0.94	0.97	0.0000045	0.88	0.86	0.90	<.0000001	0.92	0.90	0.94	<.0000001	0.93	0.91	0.95	<.0000001
Liver Disease		1.05	0.98	1.12	0.2003985	1.04	0.97	1.12	0.2365604	1.05	0.98	1.12	0.1971215	1.03	0.96	1.10	0.4057815
Pancreatic Disease		0.89	0.85	0.93	0.0000003	0.88	0.84	0.92	<.0000001	0.89	0.85	0.93	0.0000001	0.87	0.83	0.91	<.0000001
Inflammatory Bowel Disease		0.82	0.77	0.88	<.0000001	0.81	0.76	0.86	<.0000001	0.82	0.77	0.87	<.0000001	0.80	0.75	0.85	<.0000001
Rheumatologic Disease		1.06	1.03	1.08	0.0000262	1.02	0.99	1.04	0.1816280	1.04	1.01	1.07	0.0026841	0.98	0.96	1.00	0.1048958
Spinal Cord Disease or Injury		1.14	1.09	1.20	<.0000001	1.13	1.08	1.19	0.0000003	1.14	1.09	1.20	<.0000001	1.11	1.06	1.17	0.0000134
Serious Neurologic Disease		1.06	1.03	1.08	0.0000285	1.01	0.98	1.04	0.4878350	1.04	1.01	1.07	0.0022126	0.97	0.95	1.00	0.0179386
Parkinson's/Huntington's Disease		1.02	0.98	1.07	0.3318953	0.99	0.95	1.04	0.7726600	1.01	0.97	1.06	0.5927205	0.89	0.85	0.93	0.0000003
Seizure Disorder		0.89	0.86	0.92	<.0000001	0.88	0.85	0.92	<.0000001	0.89	0.86	0.92	<.0000001	0.84	0.81	0.87	<.0000001
Congestive Heart Failure		1.04	1.01	1.06	0.0019404	0.96	0.94	0.98	0.0002307	1.00	0.98	1.02	0.9734547	0.97	0.95	0.99	0.0076765
Coronary Artery Disease		0.96	0.94	0.99	0.0015668	0.93	0.91	0.96	<.0000001	0.95	0.93	0.97	0.0000132	0.95	0.93	0.97	0.0000019
Cerebrovascular Disease		0.90	0.88	0.93	<.0000001	0.89	0.87	0.91	<.0000001	0.89	0.87	0.92	<.0000001	0.91	0.89	0.94	<.0000001
Peripheral Vascular Disease		0.94	0.92	0.96	<.0000001	0.91	0.90	0.93	<.0000001	0.92	0.90	0.94	<.0000001	0.91	0.89	0.93	<.0000001
Traumatic Brain Injury		1.21	1.16	1.26	<.0000001	1.22	1.17	1.27	<.0000001	1.21	1.16	1.26	<.0000001	1.18	1.13	1.23	<.0000001
Amputee		1.04	0.92	1.17	0.5447029	1.03	0.91	1.15	0.6759613	1.04	0.93	1.17	0.5138780	1.02	0.91	1.15	0.7147438
Estrogen		0.92	0.87	0.96	0.0002199	0.86	0.82	0.90	<.0000001	0.88	0.84	0.93	0.0000002	0.83	0.79	0.87	<.0000001
Osteoporosis medications		1.20	1.17	1.24	<.0000001	1.14	1.11	1.17	<.0000001	1.16	1.13	1.20	<.0000001	1.13	1.10	1.16	<.0000001
Beta Blockers		1.55	1.52	1.58	<.0000001	1.42	1.39	1.45	<.0000001	1.46	1.43	1.49	<.0000001	1.34	1.31	1.37	<.0000001
Continuous Count of Total Drug Use (FAD & non-FAD)		-	-	-	-	1.10	1.09	1.10	<.0000001								
Total Drug Use	0 Drug									1.00							
	1 Drug									1.08	1.04	1.13	0.0004536				
	2 Drugs									1.17	1.12	1.22	<.0000001				
	3+ Drugs									1.56	1.51	1.61	<.0000001				
FAD	0 Drug													1.00			
	1 Drug													2.04	1.99	2.11	<.0000001
	2 Drugs													2.86	2.77	2.95	<.0000001
	3+ Drugs													4.50	4.36	4.65	<.0000001
NON-FAD	0 Drug													1.00			
	1 Drug													0.93	0.90	0.96	0.0000191
	2 Drugs													0.84	0.81	0.87	<.0000001
	3+ Drugs													0.74	0.72	0.77	<.0000001

Model C3 (Figure 1B)= Age Stratified analysis of exposure indicator variables for concurrent use of fracture associated drugs (FAD 1,2,3+ versus none) as well as all other drug use (Non-FAD 1,2,3+ versus none).

		Age 67-69 (hip fracture n=6,421)				Age 70-74 (hip fracture n=7,183)				Age 75-79 (hip fracture n=9,937)				Age 80-84 (hip fracture n=n=11,282)				Age 85+ (hip fracture n=2,563)			
Parameter		(HR)	Confidence	Confidence	p-value	Ratio (HR)	Confidence	Confidence	p-value	Ratio (HR)	Confidence	Confidence	p-value	Ratio (HR)	Confidence	Confidence	p-value	Ratio (HR)	Confidence	Confidence	p-value
Black		0.49	0.44	0.55	<.0000001	0.46	0.41	0.51	<.0000001	0.47	0.42	0.52	<.0000001	0.47	0.42	0.51	<.0000001	0.61	0.56	0.66	<.0000001
Hispanic		0.77	0.69	0.86	0.000004	0.67	0.60	0.75	<.0000001	0.81	0.74	0.89	0.000010	0.83	0.76	0.92	0.000151	0.92	0.83	1.01	0.077876
Asian		0.74	0.62	0.89	0.001500	0.70	0.60	0.82	0.000013	0.74	0.65	0.85	0.000019	0.76	0.66	0.86	0.000035	0.88	0.77	1.00	0.047553
Other		1.10	0.90	1.35	0.345813	1.09	0.89	1.34	0.400359	1.05	0.86	1.27	0.648979	0.99	0.81	1.21	0.904379	1.07	0.90	1.28	0.435667
Low Income Subsidy		1.04	0.93	1.17	0.496723	1.13	1.02	1.26	0.022109	1.08	0.99	1.18	0.070101	1.03	0.96	1.12	0.424994	0.91	0.85	0.98	0.009339
Disability		1.77	0.44	7.08	0.420632	1.82	0.58	5.64	0.302553	1.07	0.35	3.32	0.905407	0.53	0.08	3.78	0.528830	2.35	0.59	9.42	0.226990
Aged		1.63	0.41	6.52	0.491851	1.66	0.53	5.15	0.383524	0.97	0.31	3.03	0.961985	0.54	0.08	3.80	0.531608	2.55	0.64	10.26	0.186421
Long term care		1.86	1.71	2.03	<.0000001	1.75	1.62	1.88	<.0000001	1.60	1.52	1.69	<.0000001	1.30	1.24	1.36	<.0000001	1.05	1.01	1.09	0.014441
Dual eligible		1.07	0.94	1.20	0.308453	0.97	0.87	1.08	0.588197	0.86	0.79	0.94	0.001359	0.84	0.77	0.91	0.000041	0.87	0.81	0.93	0.000123
Osteoporosis/Osteopenia		4.06	3.80	4.33	<.0000001	3.97	3.74	4.22	<.0000001	3.54	3.37	3.72	<.0000001	3.13	2.99	3.28	<.0000001	2.84	2.72	2.97	<.0000001
Dementia		2.46	2.23	2.71	<.0000001	3.15	2.91	3.41	<.0000001	3.95	3.73	4.18	<.0000001	3.73	3.55	3.92	<.0000001	3.80	3.64	3.96	<.0000001
Obesity		1.08	1.00	1.17	0.046427	1.05	0.97	1.14	0.270863	0.91	0.84	0.99	0.029551	0.86	0.78	0.94	0.001207	0.72	0.63	0.83	0.000002
Depression		1.48	1.38	1.58	<.0000001	1.31	1.22	1.40	<.0000001	1.17	1.10	1.24	0.000000	1.19	1.13	1.26	<.0000001	1.09	1.03	1.15	0.001787
Serious Mental Illness		0.93	0.84	1.04	0.228675	1.01	0.90	1.13	0.871746	1.06	0.97	1.17	0.215288	1.27	1.17	1.39	0.000000	1.29	1.18	1.41	<.0000001
Alcohol Abuse		3.06	2.42	3.85	<.0000001	1.59	1.14	2.22	0.006958	1.52	1.06	2.20	0.024287	1.08	0.70	1.66	0.727326	0.84	0.42	1.68	0.618927
Tobacco/Chronic Lung Disease		2.59	2.43	2.76	<.0000001	2.31	2.17	2.45	<.0000001	2.14	2.03	2.26	<.0000001	1.85	1.75	1.95	<.0000001	1.94	1.83	2.05	<.0000001
Diabetes		0.99	0.94	1.05	0.824373	0.94	0.89	0.99	0.022182	0.94	0.90	0.98	0.004289	0.92	0.89	0.96	0.000128	0.87	0.84	0.91	<.0000001
Liver Disease		1.03	0.89	1.19	0.716669	1.25	1.08	1.44	0.002139	0.94	0.81	1.10	0.463581	0.93	0.79	1.10	0.418279	0.84	0.70	1.02	0.070647
Pancreatic Disease		0.89	0.80	1.00	0.042732	0.92	0.82	1.02	0.107798	0.87	0.79	0.96	0.003725	0.83	0.76	0.91	0.000063	0.84	0.77	0.92	0.000265
Inflammatory Bowel Disease		0.74	0.64	0.87	0.000187	0.81	0.70	0.94	0.004228	0.89	0.78	1.01	0.066161	0.78	0.68	0.89	0.000198	0.75	0.64	0.88	0.000262
Rheumatologic Disease		1.06	1.00	1.13	0.066325	1.06	1.00	1.12	0.072162	0.95	0.91	1.01	0.083395	0.96	0.91	1.01	0.083694	0.92	0.87	0.98	0.004427
Spinal Cord Disease or Injury		1.13	1.00	1.27	0.044723	1.18	1.05	1.32	0.006272	1.07	0.97	1.19	0.197687	0.99	0.90	1.10	0.905101	1.13	1.02	1.24	0.021885
Serious Neurologic Disease		1.04	0.98	1.11	0.197622	0.97	0.91	1.03	0.293126	0.94	0.89	1.00	0.036934	0.96	0.91	1.01	0.112406	0.90	0.85	0.95	0.000169
Parkinson's/Huntington's Disease		1.11	0.98	1.25	0.105596	1.03	0.93	1.15	0.560801	0.95	0.87	1.04	0.267755	0.82	0.75	0.89	0.000004	0.74	0.67	0.81	<.0000001
Seizure Disorder		0.91	0.83	1.00	0.038213	0.89	0.82	0.98	0.015022	0.81	0.74	0.87	<.0000001	0.76	0.70	0.82	<.0000001	0.71	0.66	0.77	<.0000001
Congestive Heart Failure		0.99	0.93	1.06	0.799222	1.03	0.98	1.10	0.264155	0.96	0.91	1.01	0.079761	1.00	0.96	1.05	0.907463	0.93	0.89	0.96	0.000146
Coronary Artery Disease		0.81	0.76	0.87	<.0000001	0.91	0.86	0.97	0.002749	0.93	0.89	0.98	0.005149	1.00	0.96	1.05	0.880317	0.99	0.95	1.04	0.736151
Cerebrovascular Disease		1.19	1.11	1.27	0.000002	1.00	0.94	1.07	0.951918	0.93	0.88	0.98	0.006702	0.86	0.82	0.91	<.0000001	0.81	0.77	0.84	<.0000001
Peripheral Vascular Disease		1.10	1.04	1.17	0.001143	0.98	0.93	1.04	0.484029	0.93	0.89	0.98	0.002111	0.87	0.83	0.90	<.0000001	0.82	0.79	0.85	<.0000001
Traumatic Brain Injury		1.23	1.08	1.40	0.001629	1.29	1.15	1.45	0.000022	1.23	1.12	1.35	0.000013	1.20	1.11	1.31	0.000010	1.11	1.03	1.20	0.007612
Amputee		1.40	1.10	1.78	0.005819	1.41	1.09	1.82	0.008362	0.84	0.64	1.11	0.215144	0.80	0.61	1.06	0.115415	0.77	0.59	1.00	0.047735
Estrogen		0.77	0.69	0.86	0.000002	0.76	0.68	0.85	0.000001	0.93	0.85	1.03	0.144166	0.85	0.77	0.94	0.002169	0.91	0.81	1.01	0.085415
Osteoporosis medications		1.39	1.29	1.50	<.0000001	1.27	1.19	1.37	<.0000001	1.10	1.04	1.17	0.001439	1.03	0.97	1.09	0.341592	1.02	0.96	1.09	0.465379
Beta Blockers		1.31	1.24	1.39	<.0000001	1.30	1.23	1.37	<.0000001	1.32	1.26	1.38	<.0000001	1.35	1.29	1.40	<.0000001	1.42	1.36	1.48	<.0000001
FAD	1 Drug	2.15	1.98	2.33	<.0000001	1.85	1.72	2.00	<.0000001	1.93	1.81	2.06	<.0000001	1.98	1.87	2.11	<.0000001	2.41	2.28	2.55	<.0000001
	2 Drugs	3.28	3.00	3.58	<.0000001	2.74	2.52	2.97	<.0000001	2.79	2.61	2.99	<.0000001	2.72	2.55	2.90	<.0000001	3.28	3.08	3.49	<.0000001
	3+ Drugs	5.68	5.21	6.19	<.0000001	4.44	4.09	4.82	<.0000001	4.40	4.10	4.73	<.0000001	4.16	3.89	4.45	<.0000001	4.78	4.49	5.09	<.0000001
NON-FAD	1 Drug	0.88	0.80	0.96	0.005234	0.92	0.84	1.00	0.061216	0.93	0.86	1.00	0.041794	0.98	0.92	1.05	0.634905	0.95	0.90	1.02	0.135165
	2 Drugs	0.73	0.67	0.80	<.0000001	0.86	0.79	0.94	0.000528	0.83	0.77	0.89	0.000001	0.90	0.84	0.96	0.001704	0.86	0.81	0.92	0.000002
	3+ Drugs	0.66	0.61	0.72	<.0000001	0.77	0.71	0.83	<.0000001	0.74	0.69	0.79	<.0000001	0.77	0.73	0.82	<.0000001	0.77	0.72	0.81	<.0000001

Men

		Model A= Sensitivity analysis excluding any drug exposure variable				Model B= Continuous exposure variable for total drug use.				Model C1=Categorized exposure indicator variables for total drug use (1,2,3+ drugs versus none).				Model C2 (Figure 1A)= Exposure indicator variables for concurrent use of fracture associated drugs (FAD 1,2,3+ versus none) as well as all other drug use (Non-FAD 1,2,3+ versus none).			
Parameter		Hazard Ratio (HR)	Lower 95% Confidence Interval	Higher 95% Confidence Interval	p-value	Hazard Ratio (HR)	Lower 95% Confidence Interval	Higher 95% Confidence Interval	p-value	Hazard Ratio (HR)	Lower 95% Confidence Interval	Higher 95% Confidence Interval	p-value	Hazard Ratio (HR)	Lower 95% Confidence Interval	Higher 95% Confidence Interval	p-value
Age (reference 67-69)																	
Age 70-74		1.24	1.17	1.33	<.0000001	1.24	1.17	1.33	<.0000001	1.25	1.18	1.34	<.0000001	1.19	1.12	1.27	0.0000001
Age 75-79		1.89	1.78	2.02	<.0000001	1.90	1.79	2.02	<.0000001	1.91	1.79	2.03	<.0000001	1.83	1.72	1.95	<.0000001
Age 80-84		3.09	2.91	3.29	<.0000001	3.11	2.92	3.31	<.0000001	3.12	2.93	3.32	<.0000001	2.98	2.80	3.18	<.0000001
Age 85+		5.63	5.29	5.99	<.0000001	5.69	5.34	6.06	<.0000001	5.66	5.32	6.03	<.0000001	5.33	5.00	5.68	<.0000001
Race/Ethnicity (reference white)																	
Black		0.52	0.48	0.57	<.0000001	0.55	0.50	0.60	<.0000001	0.54	0.49	0.58	<.0000001	0.55	0.51	0.60	<.0000001
Hispanic		0.65	0.60	0.70	<.0000001	0.68	0.63	0.74	<.0000001	0.67	0.61	0.72	<.0000001	0.70	0.64	0.76	<.0000001
Asian		0.45	0.40	0.52	<.0000001	0.46	0.41	0.53	<.0000001	0.46	0.40	0.52	<.0000001	0.50	0.44	0.57	<.0000001
Other		0.88	0.74	1.04	0.1292927	0.89	0.76	1.06	0.1878222	0.89	0.75	1.05	0.1726889	0.91	0.77	1.08	0.2836978
Low Income Subsidy		1.23	1.13	1.34	0.0000010	1.22	1.12	1.32	0.0000040	1.24	1.14	1.35	0.0000004	1.19	1.09	1.29	0.0000627
Original Medicare eligibility (%)																	
Disability		1.19	0.38	3.68	0.7687277	1.17	0.38	3.64	0.7833651	1.19	0.38	3.69	0.7639888	1.06	0.34	3.28	0.9228518
Aged		0.90	0.29	2.79	0.8549026	0.92	0.30	2.86	0.8861022	0.92	0.30	2.84	0.8780788	0.88	0.29	2.74	0.8298957
Long term care		1.55	1.48	1.63	<.0000001	1.53	1.46	1.61	<.0000001	1.54	1.46	1.62	<.0000001	1.45	1.37	1.52	<.0000001
Dual eligible		0.93	0.86	1.02	0.1227305	0.90	0.82	0.98	0.0154687	0.92	0.85	1.01	0.0664971	0.85	0.78	0.93	0.0002646
Chronic Conditions																	
Osteoporosis/Osteopenia		4.53	4.21	4.89	<.0000001	4.51	4.18	4.86	<.0000001	4.51	4.18	4.85	<.0000001	4.45	4.13	4.79	<.0000001
Dementia		4.03	3.82	4.24	<.0000001	3.99	3.79	4.20	<.0000001	3.98	3.78	4.19	<.0000001	3.79	3.60	3.99	<.0000001
Obesity		1.02	0.94	1.10	0.6749073	0.96	0.89	1.04	0.3335763	1.00	0.93	1.08	0.9211443	0.96	0.88	1.03	0.2404810
Depression		1.53	1.44	1.62	<.0000001	1.43	1.35	1.51	<.0000001	1.49	1.41	1.58	<.0000001	1.27	1.20	1.34	<.0000001
Serious Mental Illness		1.43	1.32	1.54	<.0000001	1.41	1.30	1.52	<.0000001	1.42	1.31	1.54	<.0000001	1.31	1.21	1.42	<.0000001
Alcohol Abuse		1.67	1.46	1.91	<.0000001	1.76	1.53	2.01	<.0000001	1.71	1.50	1.96	<.0000001	1.70	1.48	1.94	<.0000001
Tobacco/Chronic Lung Disease		2.60	2.49	2.71	<.0000001	2.52	2.41	2.63	<.0000001	2.55	2.45	2.67	<.0000001	2.54	2.44	2.66	<.0000001
Diabetes		0.90	0.87	0.94	0.0000001	0.83	0.80	0.86	<.0000001	0.86	0.83	0.90	<.0000001	0.87	0.84	0.90	<.0000001
Liver Disease		1.11	1.00	1.24	0.0569526	1.10	0.99	1.23	0.0748928	1.11	1.00	1.24	0.0508873	1.07	0.96	1.19	0.2202379
Pancreatic Disease		0.91	0.83	1.00	0.0379488	0.90	0.83	0.99	0.0244078	0.91	0.83	0.99	0.0328794	0.88	0.80	0.96	0.0038325
Inflammatory Bowel Disease		1.00	0.89	1.14	0.9626643	0.99	0.87	1.12	0.8228687	1.00	0.88	1.13	0.9495637	0.98	0.87	1.11	0.7982742
Rheumatologic Disease		1.07	1.01	1.13	0.0193104	1.04	0.98	1.10	0.1772737	1.06	1.00	1.12	0.0553019	1.00	0.95	1.06	0.9445934
Spinal Cord Disease or Injury		1.11	1.02	1.21	0.0145727	1.10	1.01	1.20	0.0270094	1.11	1.02	1.21	0.0127472	1.10	1.01	1.19	0.0355464
Serious Neurologic Disease		1.13	1.08	1.18	0.0000005	1.08	1.03	1.13	0.0014721	1.11	1.06	1.17	0.0000105	1.03	0.98	1.08	0.2921994
Parkinson's/Huntington's Disease		1.42	1.33	1.51	<.0000001	1.38	1.29	1.46	<.0000001	1.40	1.31	1.49	<.0000001	1.20	1.12	1.27	<.0000001
Seizure Disorder		0.99	0.93	1.06	0.8343915	0.98	0.92	1.05	0.5768890	0.99	0.93	1.05	0.6936826	0.90	0.84	0.96	0.0009620
Congestive Heart Failure		1.17	1.12	1.22	<.0000001	1.09	1.04	1.14	0.0000668	1.13	1.09	1.18	<.0000001	1.10	1.05	1.14	0.0000201
Coronary Artery Disease		0.90	0.86	0.94	0.0000004	0.88	0.84	0.91	<.0000001	0.89	0.85	0.92	<.0000001	0.90	0.86	0.94	0.0000007
Cerebrovascular Disease		1.03	0.98	1.07	0.2883028	1.01	0.97	1.06	0.6587586	1.02	0.97	1.06	0.5004384	1.03	0.99	1.08	0.1697974
Peripheral Vascular Disease		1.06	1.01	1.10	0.0091996	1.02	0.98	1.06	0.3292395	1.03	0.99	1.07	0.1562596	1.02	0.98	1.06	0.4227959
Traumatic Brain Injury		1.31	1.21	1.41	<.0000001	1.33	1.23	1.44	<.0000001	1.32	1.22	1.43	<.0000001	1.30	1.20	1.40	<.0000001
Amputee		1.38	1.21	1.57	0.0000024	1.37	1.20	1.57	0.0000031	1.38	1.21	1.57	0.0000020	1.37	1.20	1.56	0.0000033
Estrogen		0.68	0.28	1.64	0.3926226	0.62	0.26	1.49	0.2814806	0.65	0.27	1.56	0.3361772	0.52	0.22	1.25	0.1442681
Osteoporosis medications		0.99	0.87	1.12	0.8115713	0.90	0.79	1.02	0.0960561	0.94	0.83	1.07	0.3588648	0.86	0.75	0.97	0.0180487
Beta Blockers		1.52	1.46	1.59	<.0000001	1.36	1.30	1.41	<.0000001	1.41	1.35	1.47	<.0000001	1.26	1.21	1.32	<.0000001
Continuous Count of Total Drug Use (FAD & non-FAD)		-	-	-	-	1.10	1.09	1.11	<.0000001								
Total Drug Use	0 Drug									1.00							
	1 Drug									1.07	0.99	1.17	0.0956744				
	2 Drugs									1.13	1.05	1.22	0.0018700				
	3+ Drugs									1.55	1.46	1.65	<.0000001				
FAD	0 Drug													1.00			
	1 Drug													2.23	2.11	2.36	<.0000001
	2 Drugs													3.40	3.20	3.61	<.0000001
	3+ Drugs													5.18	4.87	5.52	<.0000001
NON-FAD	0 Drug													1.00			
	1 Drug													0.90	0.84	0.96	0.0011528
	2 Drugs													0.84	0.79	0.89	<.0000001
	3+ Drugs													0.71	0.67	0.75	<.0000001

Model C3 (Figure 1C)= Age Stratified analysis of exposure indicator variables for concurrent use of fracture associated drugs (FAD 1,2,3+ versus none) as well as all other drug use (Non-FAD 1,2,3+ versus none).

		Age 67-69 (hip fracture n=2,531)				Age 70-74 (hip fracture n=2,300)				Age 75-79 (hip fracture n=2,639)				Age 80-84 (hip fracture n=2,465)				Age 85+ (hip fracture n=2,382)			
Parameter		Hazard Ratio (HR)	Lower 95% Confidence Interval	Higher 95% Confidence Interval	p-value	Hazard Ratio (HR)	Lower 95% Confidence Interval	Higher 95% Confidence Interval	p-value	Hazard Ratio (HR)	Lower 95% Confidence Interval	Higher 95% Confidence Interval	p-value	Hazard Ratio (HR)	Lower 95% Confidence Interval	Higher 95% Confidence Interval	p-value	Hazard Ratio (HR)	Lower 95% Confidence Interval	Higher 95% Confidence Interval	p-value
Black		0.57	0.48	0.68	<.0000001	0.61	0.51	0.74	0.000000	0.43	0.35	0.53	<.0000001	0.62	0.51	0.76	0.000002	0.43	0.33	0.55	<.0000001
Hispanic		0.75	0.63	0.89	0.001000	0.65	0.54	0.79	0.000010	0.61	0.50	0.73	0.000000	0.87	0.73	1.05	0.143907	0.71	0.58	0.88	0.001755
Asian		0.49	0.35	0.69	0.000038	0.55	0.41	0.74	0.000073	0.44	0.33	0.58	<.0000001	0.56	0.43	0.74	0.000037	0.52	0.39	0.69	0.000004
Other		0.89	0.66	1.22	0.480472	0.98	0.70	1.38	0.918198	0.72	0.48	1.08	0.114190	0.96	0.62	1.49	0.849820	1.10	0.71	1.69	0.678910
Low Income Subsidy		1.11	0.91	1.35	0.327521	1.23	1.01	1.51	0.043557	1.25	1.04	1.50	0.019812	1.18	0.99	1.42	0.072028	1.26	1.06	1.49	0.008008
Disability		0.00	0.00	0.00	0.918105	0.00	0.00	0.00	0.928170	0.85	0.12	6.05	0.871904	17.07	4.25	68.52	0.000063	0.00	0.00	0.00	0.949397
Aged		0.00	0.00	0.00	0.915780	0.00	0.00	0.00	0.927191	0.86	0.12	6.13	0.880002	15.60	3.86	63.00	0.000115	0.00	0.00	0.00	0.950931
Long term care		1.91	1.67	2.17	<.0000001	1.90	1.66	2.18	<.0000001	1.59	1.42	1.78	<.0000001	1.23	1.11	1.37	0.000104	1.07	0.97	1.17	0.200700
Dual eligible		1.20	0.98	1.47	0.076721	0.96	0.78	1.18	0.682322	0.78	0.65	0.95	0.014331	0.72	0.60	0.88	0.000904	0.68	0.57	0.81	0.000024
Osteoporosis/Osteopenia		5.21	4.38	6.19	<.0000001	4.08	3.37	4.95	<.0000001	4.74	4.03	5.56	<.0000001	4.47	3.82	5.24	<.0000001	4.38	3.75	5.11	<.0000001
Dementia		2.65	2.30	3.04	<.0000001	3.11	2.71	3.56	<.0000001	4.47	4.00	4.99	<.0000001	4.74	4.27	5.25	<.0000001	4.12	3.74	4.54	<.0000001
Obesity		0.97	0.85	1.11	0.662054	0.93	0.79	1.09	0.366819	0.82	0.69	0.97	0.024035	1.05	0.86	1.29	0.621565	0.89	0.66	1.20	0.447464
Depression		1.44	1.28	1.63	<.0000001	1.34	1.17	1.53	0.000025	1.27	1.12	1.44	0.000164	1.17	1.03	1.33	0.020062	1.06	0.92	1.22	0.392228
Serious Mental Illness		0.99	0.85	1.16	0.906020	1.17	0.98	1.40	0.084260	1.17	0.98	1.40	0.093130	1.66	1.39	1.98	<.0000001	1.28	1.04	1.59	0.020416
Alcohol Abuse		1.64	1.33	2.02	0.000004	1.29	0.97	1.72	0.082544	1.52	1.11	2.06	0.008206	1.45	0.97	2.16	0.069517	0.66	0.27	1.59	0.353600
Tobacco/Chronic Lung Disease		3.00	2.73	3.30	<.0000001	2.87	2.60	3.17	<.0000001	2.47	2.25	2.70	<.0000001	2.23	2.02	2.45	<.0000001	2.09	1.88	2.32	<.0000001
Diabetes		0.93	0.85	1.02	0.112535	0.85	0.78	0.93	0.000497	0.90	0.83	0.98	0.015899	0.82	0.76	0.90	0.000008	0.85	0.78	0.93	0.000327
Liver Disease		1.01	0.83	1.22	0.925833	1.16	0.94	1.45	0.173561	0.97	0.76	1.23	0.790006	0.99	0.74	1.32	0.924961	0.96	0.67	1.38	0.818423
Pancreatic Disease		0.97	0.81	1.16	0.721830	0.82	0.67	1.02	0.071216	0.84	0.69	1.02	0.075144	0.83	0.68	1.02	0.080619	0.95	0.78	1.17	0.650708
Inflammatory Bowel Disease		1.09	0.85	1.40	0.492856	1.11	0.86	1.44	0.423851	1.08	0.84	1.40	0.552178	0.90	0.66	1.22	0.487844	0.67	0.47	0.96	0.027782
Rheumatologic Disease		1.14	1.00	1.28	0.045005	0.99	0.87	1.13	0.872952	0.93	0.82	1.05	0.221220	0.94	0.82	1.07	0.343030	1.12	0.98	1.28	0.101937
Spinal Cord Disease or Injury		1.22	1.04	1.43	0.016632	1.09	0.90	1.32	0.382023	0.88	0.73	1.07	0.203580	1.00	0.82	1.22	0.990004	1.19	0.97	1.47	0.103250
Serious Neurologic Disease		1.06	0.96	1.18	0.233899	1.14	1.03	1.27	0.014088	1.06	0.96	1.17	0.289245	0.89	0.80	0.99	0.037071	0.92	0.82	1.03	0.156311
Parkinson's/Huntington's Disease		1.45	1.26	1.69	0.000001	1.43	1.23	1.65	0.000002	1.24	1.09	1.41	0.001058	1.11	0.97	1.27	0.122113	0.91	0.78	1.06	0.209984
Seizure Disorder		0.95	0.84	1.09	0.479772	0.81	0.70	0.94	0.006074	0.95	0.84	1.09	0.492711	0.80	0.69	0.93	0.003134	0.71	0.60	0.84	0.000092
Congestive Heart Failure		1.03	0.94	1.14	0.506936	1.08	0.98	1.19	0.128634	1.18	1.08	1.29	0.000282	1.16	1.06	1.27	0.000940	0.98	0.90	1.08	0.706801
Coronary Artery Disease		0.78	0.71	0.86	0.000001	0.90	0.81	0.99	0.026457	0.90	0.82	0.99	0.021268	0.93	0.85	1.01	0.092862	1.02	0.93	1.12	0.708320
Cerebrovascular Disease		1.32	1.19	1.47	0.000000	1.23	1.11	1.37	0.000119	1.03	0.93	1.13	0.610479	0.93	0.84	1.02	0.125750	0.76	0.69	0.85	0.000000
Peripheral Vascular Disease		1.05	0.96	1.15	0.293747	1.01	0.92	1.11	0.819072	1.06	0.97	1.15	0.205913	0.97	0.89	1.06	0.439279	0.95	0.87	1.04	0.237426
Traumatic Brain Injury		1.35	1.13	1.60	0.000744	1.68	1.42	2.00	<.0000001	1.15	0.96	1.37	0.122027	1.24	1.04	1.48	0.017558	1.17	0.98	1.39	0.081451
Amputee		1.91	1.54	2.37	<.0000001	1.43	1.08	1.89	0.013722	1.02	0.74	1.42	0.895252	1.03	0.71	1.48	0.883465	0.74	0.47	1.18	0.208920
Estrogen		0.89	0.22	3.56	0.864695	0.75	0.11	5.32	0.770501	0.00	0.00	0.00	0.920327	0.00	0.00	0.00	0.912962	0.66	0.16	2.65	0.556981
Osteoporosis medications		0.66	0.48	0.91	0.011169	1.25	0.94	1.65	0.124524	0.90	0.69	1.17	0.416641	0.71	0.52	0.96	0.024852	0.80	0.61	1.06	0.115273
Beta Blockers		1.20	1.09	1.31	0.000120	1.23	1.11	1.35	0.000031	1.35	1.23	1.47	<.0000001	1.30	1.19	1.42	<.0000001	1.29	1.17	1.41	0.000000
FAD	1 Drug	2.26	2.00	2.55	<.0000001	2.03	1.79	2.30	<.0000001	2.00	1.78	2.25	<.0000001	2.41	2.14	2.72	<.0000001	2.69	2.37	3.05	<.0000001
	2 Drugs	3.55	3.10	4.06	<.0000001	3.25	2.82	3.73	<.0000001	3.16	2.77	3.59	<.0000001	3.44	3.01	3.94	<.0000001	4.13	3.60	4.75	<.0000001
	3+ Drugs	6.09	5.32	6.97	<.0000001	4.85	4.18	5.61	<.0000001	4.57	3.99	5.24	<.0000001	5.16	4.48	5.94	<.0000001	6.02	5.20	6.96	<.0000001
NON-FAD	1 Drug	0.82	0.71	0.95	0.006783	0.89	0.76	1.04	0.126793	0.98	0.85	1.14	0.797947	0.94	0.81	1.09	0.411367	0.94	0.81	1.09	0.424149
	2 Drugs	0.75	0.65	0.86	0.000056	0.81	0.70	0.95	0.007596	0.89	0.77	1.03	0.103180	0.90	0.78	1.04	0.154285	0.93	0.81	1.08	0.346405
	3+ Drugs	0.58	0.51	0.66	<.0000001	0.71	0.62	0.81	0.000000	0.80	0.70	0.91	0.000487	0.79	0.70	0.91	0.000515	0.75	0.66	0.85	0.000011

Estimates are from Cox regression models. Total person years of observation = 7,126,266 for women, 4,160,502 for men. Total Drug Use = fracture associated drugs (FADs) + non-FADS. For FADs, status of receipt of 21 single drug (1 FAD) and 210 concurrent drug pairs (2 FAD) is classified for each person, for each day of observation. 3+ FAD is a classification of person days with 3 or more concurrent FADs. The same approach was taken to identify 552 non-FAD exposures: 1 non-FAD expsoure (1 Non-FAD), 2 concurrent non-FAD exposures (2 Non-FAD), 3+ Non-FAD is a classification of person days with 3 or more concurrent Non-FADs. Model A= Sensitivity analysis excluding any drug exposure variable. Model B= Continuous exposure variable for total drug use. Model C1=Categorized exposure indicator variables for total drug use (1,2,3+ drugs versus none). Model C2=Exposure indicator variables for concurrent use of fracture associated drugs (FAD 1,2,3+ versus none) as well as all other drug use (Non-FAD 1,2,3+ versus none). Model C3= Model C2 stratified on age category (5 year increments except for the first and the last). All models were stratified on sex and adjusted for time varying patient factors; Medicaid eligibility and Medicare Part D low income subsidy are two poverty indicators. Long-term care indicator is defined as 50% or more annual prescription fills dispensed by a long-term care pharmacy. The three categories of drugs protective of fracture are: 1. osteoporosis treatments: oral and injected bisphosphonates, calcitonin, denosumab, parathyroid hormone, 2. Estrogens- systemic estrogens, selective estrogen receptor modulators, and 3. beta-blockers. The "Tobacco/Chronic Obstructive Lung Disease" co-morbidity includes patients with Chronic Obstructive Lung Disease diagnosis and/or tobacco use diagnosis.

© 2019 Emeny RT et al. *JAMA Network Open.*

eTable 7. Cox Proportional Hazard Model Output: Hip Fracture Risk Associated With Fracture-Associated Drugs Among Women

In 20% Medicare sample, person-years of exposure and crude fracture rate per exposure group, adjusted for sociodemographic factors, comorbidities, non-fracture-associated drug use (person-years, 7 126 266).

			Cox Regression Model (Hip Fracture n = 47,386)				
Parameter	Person Years	Fracture Rate (# Fracture per 1000 PY)	Hazard Ratio (HR)	Lower 95% Confidence Interval	Higher 95% Confidence Interval	p-value	False Discovery Rate p-value
**Age (reference 67-69)**							
**Age 70-74**	-	-	**1.24**	**1.19**	**1.29**	**2.70E-24**	**1.30E-23**
**Age 75-79**	-	-	**2.10**	**2.02**	**2.18**	**1.00E-300**	**4.00E-299**
**Age 80-84**	-	-	**3.24**	**3.12**	**3.37**	**0.00E+00**	**0.00E+00**
**Age 85+**	-	-	**5.09**	**4.90**	**5.28**	**0.00E+00**	**0.00E+00**
**Race/Ethnicity (reference white)**							
**Black**	-	-	**0.51**	**0.49**	**0.54**	**3.00E-192**	**7.00E-191**
**Hispanic**	-	-	**0.81**	**0.77**	**0.84**	**7.50E-21**	**3.00E-20**
**Asian**	-	-	**0.78**	**0.73**	**0.83**	**1.20E-13**	**3.10E-13**
Other	-	-	1.07	0.98	1.17	1.20E-01	1.40E-01
Low Income Subsidy	-	-	1.02	0.98	1.06	3.40E-01	3.80E-01
**Original Medicare eligibility (%)**							
Disability	-	-	1.28	0.71	2.30	4.20E-01	4.60E-01
Aged	-	-	1.09	0.60	1.97	7.70E-01	8.00E-01
**Long term care**	-	-	**1.33**	**1.29**	**1.36**	**7.00E-114**	**1.00E-112**
**Dual eligible**	-	-	**0.89**	**0.85**	**0.92**	**3.90E-09**	**8.00E-09**
**Chronic Conditions**							
**Osteoporosis/Osteopenia**	-	-	**3.28**	**3.21**	**3.36**	**0.00E+00**	**0.00E+00**
**Dementia**	-	-	**3.46**	**3.38**	**3.55**	**0.00E+00**	**0.00E+00**
Obesity	-	-	1.00	0.96	1.04	8.60E-01	8.80E-01
**Depression**	-	-	**1.22**	**1.19**	**1.26**	**7.40E-49**	**5.50E-48**
**Serious Mental Illness**	-	-	**1.22**	**1.16**	**1.27**	**9.90E-19**	**3.50E-18**
**Alcohol Abuse**	-	-	**1.90**	**1.63**	**2.21**	**1.70E-16**	**5.30E-16**
**Tobacco/Chronic Lung Disease**	-	-	**2.17**	**2.12**	**2.23**	**0.00E+00**	**0.00E+00**
**Diabetes**	-	-	**0.93**	**0.91**	**0.95**	**9.90E-14**	**2.60E-13**
Liver Disease	-	-	1.03	0.96	1.11	3.80E-01	4.20E-01
**Pancreatic Disease**	-	-	**0.87**	**0.83**	**0.91**	**3.10E-10**	**6.60E-10**
**Inflammatory Bowel Disease**	-	-	**0.80**	**0.75**	**0.86**	**1.40E-11**	**3.30E-11**
Rheumatologic Disease	-	-	**0.97**	**0.94**	**0.99**	**7.50E-03**	**1.00E-02**
**Spinal Cord Disease or Injury**	-	-	**1.11**	**1.06**	**1.16**	**3.10E-05**	**5.20E-05**
Serious Neurologic Disease	-	-	**0.96**	**0.94**	**0.99**	**4.50E-03**	**6.20E-03**
**Parkinson's/Huntington's Disease**	-	-	**0.86**	**0.82**	**0.90**	**4.20E-11**	**9.30E-11**
**Seizure Disorder**	-	-	**0.84**	**0.81**	**0.87**	**5.60E-20**	**2.10E-19**
**Congestive Heart Failure**	-	-	**0.96**	**0.94**	**0.98**	**1.20E-04**	**1.80E-04**
**Coronary Artery Disease**	-	-	**0.95**	**0.93**	**0.98**	**5.60E-05**	**9.00E-05**
**Cerebrovascular Disease**	-	-	**0.92**	**0.89**	**0.94**	**1.80E-12**	**4.40E-12**
**Peripheral Vascular Disease**	-	-	**0.91**	**0.89**	**0.93**	**6.20E-19**	**2.20E-18**
**Traumatic Brain Injury**	-	-	**1.18**	**1.13**	**1.23**	**3.60E-14**	**9.60E-14**
Amputee	-	-	1.02	0.90	1.14	7.90E-01	8.20E-01
**Estrogen**	-	-	**0.83**	**0.79**	**0.87**	**8.70E-15**	**2.40E-14**
**Osteoporosis medications**	-	-	**1.14**	**1.10**	**1.17**	**3.30E-18**	**1.10E-17**
**Beta Blockers**	-	-	**1.35**	**1.32**	**1.38**	**3.00E-177**	**6.00E-176**
**Single FAD Exposures**							
**Thiazide Diuretics**	**733,478**	**4.35**	**1.74**	**1.67**	**1.82**	**5.00E-147**	**9.00E-146**
**Proton Pump Inhibitors (PPI)**	**252,621**	**5.30**	**1.71**	**1.62**	**1.82**	**1.20E-73**	**1.10E-72**
**SSRI/SNRI**	**185,004**	**8.98**	**2.32**	**2.20**	**2.44**	**9.00E-207**	**2.00E-205**
**Loop Diuretics**	**146,816**	**10.97**	**2.45**	**2.32**	**2.59**	**3.00E-222**	**8.00E-221**
**Opioids**	**81,726**	**11.45**	**3.26**	**3.04**	**3.49**	**2.00E-253**	**6.00E-252**
**Anticonvulsants**	**60,055**	**7.51**	**2.24**	**2.04**	**2.47**	**2.60E-61**	**2.10E-60**
**Sedative Hypnotics**	**53,415**	**4.79**	**1.59**	**1.41**	**1.81**	**2.10E-13**	**5.30E-13**
**Inhaled Glucocorticoids**	**40,444**	**5.29**	**1.79**	**1.57**	**2.06**	**4.10E-17**	**1.30E-16**
**H2 Receptor Antagonists (H2RA)**	**37,878**	**5.97**	**1.59**	**1.39**	**1.81**	**7.50E-12**	**1.80E-11**
**Oral Glucocorticoids**	**37,674**	**7.59**	**2.29**	**2.03**	**2.58**	**9.40E-43**	**6.40E-42**
**Nitrates**	**32,143**	**7.44**	**1.62**	**1.43**	**1.85**	**2.20E-13**	**5.50E-13**
**Tricyclic Antidepressants (TCA)**	**30,279**	**4.56**	**1.60**	**1.35**	**1.89**	**4.30E-08**	**8.30E-08**
**Thiazolidinediones (TZD)**	**29,469**	**6.11**	**2.62**	**2.26**	**3.04**	**4.10E-37**	**2.70E-36**
**Benzodiazepine**	**25,085**	**7.10**	**2.38**	**2.05**	**2.77**	**3.10E-30**	**1.90E-29**
**Central Acting Antihypertensives (CAAH)**	**20,473**	**5.47**	**1.67**	**1.39**	**2.01**	**7.60E-08**	**1.50E-07**
**2nd Generation Antipsychotics (SGAP)**	**19,480**	**20.89**	**2.50**	**2.26**	**2.76**	**5.00E-71**	**4.60E-70**
**Anti Parkinson's Agents**	**19,373**	**12.18**	**3.29**	**2.89**	**3.76**	**2.50E-70**	**2.20E-69**
**1st Generation Antihistamines (H1 Blockers)**	**12,368**	**7.60**	**1.99**	**1.62**	**2.44**	**3.50E-11**	**7.90E-11**
**Barbiturates**	**3,308**	**10.58**	**2.74**	**1.97**	**3.82**	**2.60E-09**	**5.40E-09**
**1st Generation Antipsychotics (FGAP)**	**3,230**	**8.36**	**1.54**	**1.05**	**2.24**	**2.60E-02**	**3.30E-02**
Muscle Relaxers	2,986	<11	1.10	0.55	2.19	8.00E-01	8.20E-01

© 2019 Emeny RT et al. *JAMA Network Open.*

eTable 7 continued

**Concurrent Exposure to 2 FADs**							
**PPI & Thiazide Diuretics**	**116,220**	**4.68**	**1.93**	**1.76**	**2.10**	**1.20E-48**	**8.70E-48**
**SSRI/SNRI & Thiazide Diuretics**	**70,540**	**7.57**	**2.59**	**2.37**	**2.83**	**1.90E-98**	**2.30E-97**
**PPI & SSRI/SNRI**	**49,199**	**10.51**	**2.59**	**2.36**	**2.83**	**2.60E-95**	**3.10E-94**
**PPI & Loop Diuretics**	**34,016**	**12.85**	**2.67**	**2.42**	**2.94**	**2.80E-86**	**3.10E-85**
**Thiazide Diuretics & Opioids**	**32,651**	**12.07**	**4.45**	**4.02**	**4.92**	**6.00E-181**	**1.00E-179**
**Anticonvulsants & Thiazide Diuretics**	**22,172**	**5.59**	**2.11**	**1.77**	**2.52**	**2.00E-16**	**6.20E-16**
PPI & Opioids	***20,891***	***14.31***	***4.00***	***3.56***	***4.49***	***8.00E-121***	***1.00E-119***
**Thiazide Diuretics & Loop Diuretics**	**20,555**	**11.48**	**3.42**	**3.00**	**3.90**	**2.30E-76**	**2.30E-75**
SSRI/SNRI & Loop Diuretics	***19,946***	***20.46***	***3.05***	***2.75***	***3.37***	***2.00E-103***	***3.00E-102***
**Sedative Hypnotics & Thiazide Diuretics**	**18,496**	**6.33**	**2.44**	**2.03**	**2.92**	**1.40E-21**	**5.80E-21**
**TZD & Thiazide Diuretics**	**17,475**	**5.32**	**2.81**	**2.29**	**3.45**	**6.30E-23**	**2.90E-22**
**H2RA & Thiazide Diuretics**	**17,259**	**5.33**	**2.00**	**1.63**	**2.46**	**3.70E-11**	**8.30E-11**
**CAAH & Thiazide Diuretics**	**17,147**	**5.89**	**2.35**	**1.93**	**2.86**	**1.70E-17**	**5.60E-17**
SSRI/SNRI & Opioids	***14,980***	***17.29***	***3.91***	***3.46***	***4.43***	***3.00E-102***	***4.00E-101***
**Inhaled Glucocorticoids & Thiazide Diuretics**	**14,174**	**5.43**	**2.18**	**1.74**	**2.73**	**1.00E-11**	**2.40E-11**
**PPI & Anticonvulsants**	**13,998**	**8.14**	**2.39**	**1.99**	**2.88**	**3.20E-20**	**1.20E-19**
**SSRI/SNRI & Sedative Hypnotics**	**13,415**	**10.21**	**3.15**	**2.66**	**3.73**	**2.50E-40**	**1.70E-39**
Opioids & Loop Diuretics	***13,206***	***20.14***	***4.48***	***3.96***	***5.07***	***9.00E-126***	***1.00E-124***
**Anticonvulsants & SSRI/SNRI**	**12,976**	**9.71**	**2.41**	**2.02**	**2.88**	**1.60E-22**	**7.20E-22**
**TCA & Thiazide Diuretics**	**12,787**	**6.02**	**2.46**	**1.96**	**3.08**	**4.60E-15**	**1.30E-14**
Nitrates & Loop Diuretics	***12,579***	***17.57***	***3.25***	***2.84***	***3.72***	***1.90E-65***	***1.60E-64***
**Thiazide Diuretics & Nitrates**	**12,383**	**8.96**	**2.51**	**2.08**	**3.02**	**1.00E-21**	**4.30E-21**
**SSRI/SNRI & SGAP**	**12,179**	**25.54**	**2.96**	**2.64**	**3.32**	**1.00E-76**	**1.00E-75**
**PPI & Sedative Hypnotics**	**11,389**	**8.43**	**2.71**	**2.22**	**3.32**	**3.00E-22**	**1.30E-21**
**Oral Glucocorticoids & Thiazide Diuretics**	**11,212**	**7.31**	**2.59**	**2.08**	**3.22**	**1.20E-17**	**4.00E-17**
**Anticonvulsants & Opioids**	**9,695**	**12.58**	**3.79**	**3.17**	**4.54**	**4.40E-48**	**3.10E-47**
**PPI & Nitrates**	**8,589**	**9.90**	**2.02**	**1.63**	**2.50**	**1.50E-10**	**3.30E-10**
**Anticonvulsants & Loop Diuretics**	**8,566**	**13.42**	**2.88**	**2.39**	**3.47**	**4.00E-29**	**2.30E-28**
**Inhaled Glucocorticoids & PPI**	**8,525**	**6.22**	**2.10**	**1.60**	**2.75**	**8.10E-08**	**1.50E-07**
**Benzodiazepine & Thiazide Diuretics**	**8,214**	**7.79**	**3.03**	**2.37**	**3.88**	**1.20E-18**	**4.20E-18**
**Oral Glucocorticoids & PPI**	**7,847**	**10.20**	**2.73**	**2.19**	**3.41**	**4.30E-19**	**1.60E-18**
SSRI/SNRI & Benzodiazepine	***7,752***	***15.87***	***4.50***	***3.76***	***5.38***	***6.40E-61***	***5.10E-60***
**PPI & TCA**	**7,680**	**5.08**	**1.84**	**1.35**	**2.53**	**1.40E-04**	**2.10E-04**
**H2RA & Loop Diuretics**	**6,880**	**13.81**	**2.73**	**2.23**	**3.34**	**3.10E-22**	**1.30E-21**
**H2RA & SSRI/SNRI**	**6,279**	**12.10**	**2.19**	**1.74**	**2.74**	**1.20E-11**	**2.80E-11**
**Antiparkinson Agents & Thiazide Diuretics**	**6,278**	**8.28**	**2.78**	**2.11**	**3.65**	**2.40E-13**	**5.90E-13**
**CAAH & Loop Diuretics**	**6,147**	**6.34**	**1.82**	**1.33**	**2.49**	**2.10E-04**	**3.20E-04**
**PPI & H2RA**	**6,039**	**6.79**	**2.23**	**1.64**	**3.03**	**3.00E-07**	**5.60E-07**
Sedative Hypnotics & Opioids	***5,905***	***15.41***	***4.90***	***3.98***	***6.02***	***3.50E-51***	***2.70E-50***
**PPI & Benzodiazepine**	**5,691**	**9.14**	**2.97**	**2.26**	**3.91**	**5.40E-15**	**1.50E-14**
**SSRI/SNRI & Antiparkinson Agents**	**5,266**	**16.90**	**3.57**	**2.89**	**4.41**	**3.10E-32**	**2.00E-31**
**Inhaled Glucocorticoids & Loop Diuretics**	**5,204**	**11.72**	**2.47**	**1.92**	**3.18**	**2.30E-12**	**5.60E-12**
**Inhaled Glucocorticoids & SSRI/SNRI**	**4,871**	**13.34**	**3.29**	**2.58**	**4.21**	**1.30E-21**	**5.40E-21**
**SSRI/SNRI & TCA**	**4,519**	**9.74**	**3.16**	**2.35**	**4.25**	**2.70E-14**	**7.20E-14**
**Oral Glucocorticoids & SSRI/SNRI**	**4,482**	**16.29**	**3.67**	**2.92**	**4.63**	**2.50E-28**	**1.40E-27**
**TZD & Loop Diuretics**	**4,463**	**9.63**	**3.48**	**2.58**	**4.70**	**4.00E-16**	**1.20E-15**
**Oral Glucocorticoids & Loop Diuretics**	**4,323**	**12.49**	**2.45**	**1.87**	**3.20**	**5.80E-11**	**1.30E-10**
**PPI & TZD**	**4,212**	**9.26**	**3.91**	**2.86**	**5.36**	**2.20E-17**	**7.20E-17**
**Sedative Hypnotics & Loop Diuretics**	**4,177**	**16.04**	**3.36**	**2.64**	**4.28**	**6.30E-23**	**2.90E-22**
**H1 Blockers & Thiazide Diuretics**	**4,138**	**7.49**	**2.49**	**1.75**	**3.54**	**4.20E-07**	**7.70E-07**
**SSRI/SNRI & Nitrates**	**4,029**	**16.38**	**2.50**	**1.96**	**3.19**	**1.50E-13**	**3.80E-13**
**PPI & Antiparkinson Agents**	**3,797**	**14.49**	**3.44**	**2.63**	**4.48**	**1.10E-19**	**4.10E-19**
**PPI & CAAH**	**3,721**	**6.45**	**1.88**	**1.26**	**2.81**	**2.00E-03**	**2.80E-03**
**Oral Glucocorticoids & Opioids**	**3,581**	**16.20**	**4.46**	**3.44**	**5.77**	**1.10E-29**	**6.60E-29**
**PPI & SGAP**	**3,504**	**29.97**	**2.94**	**2.42**	**3.57**	**9.80E-28**	**5.20E-27**
**H2RA & Opioids**	**3,404**	**14.98**	**3.69**	**2.80**	**4.86**	**1.70E-20**	**6.70E-20**
**Thiazide Diuretics & SGAP**	**3,352**	**22.97**	**3.65**	**2.92**	**4.58**	**1.60E-29**	**9.40E-29**
**TCA & Loop Diuretics**	**3,224**	**8.69**	**2.29**	**1.58**	**3.32**	**1.20E-05**	**2.10E-05**
**Benzodiazepine & Opioids**	**3,139**	**15.61**	**4.50**	**3.40**	**5.96**	**1.10E-25**	**5.70E-25**
**TCA & Opioids**	**3,118**	**14.75**	**5.05**	**3.78**	**6.75**	**6.90E-28**	**3.70E-27**
**SGAP & Loop Diuretics**	**2,999**	**24.01**	**2.22**	**1.76**	**2.81**	**1.80E-11**	**4.20E-11**
**TZD & SSRI/SNRI**	**2,934**	**11.93**	**3.84**	**2.75**	**5.35**	**2.30E-15**	**6.60E-15**
**Anticonvulsants & Sedative Hypnotics**	**2,831**	**9.19**	**2.98**	**2.03**	**4.39**	**2.60E-08**	**5.10E-08**
**PPI & H1 Blockers**	**2,655**	**13.18**	**3.53**	**2.53**	**4.92**	**9.80E-14**	**2.60E-13**
**Anticonvulsants & TCA**	**2,548**	**5.50**	**1.84**	**1.09**	**3.11**	**2.20E-02**	**2.80E-02**
**SSRI/SNRI & CAAH**	**2,469**	**10.13**	**2.33**	**1.57**	**3.45**	**2.50E-05**	**4.30E-05**
**Nitrates & Opioids**	**2,383**	**18.04**	**4.02**	**2.98**	**5.43**	**1.10E-19**	**4.10E-19**
**Antiparkinson Agents & Loop Diuretics**	**2,369**	**16.04**	**2.96**	**2.15**	**4.08**	**2.80E-11**	**6.40E-11**
**H2RA & Anticonvulsants**	**2,279**	**8.78**	**1.93**	**1.25**	**3.00**	**3.30E-03**	**4.60E-03**
**Benzodiazepine & Loop Diuretics**	**2,172**	**21.18**	**4.61**	**3.45**	**6.17**	**6.00E-25**	**3.00E-24**
**Anticonvulsants & SGAP**	**2,134**	**16.40**	**2.27**	**1.62**	**3.16**	**1.50E-06**	**2.60E-06**
**Inhaled Glucocorticoids & Oral Glucocorticoic**	**2,091**	**11.48**	**3.33**	**2.23**	**4.97**	**4.10E-09**	**8.30E-09**

© 2019 Emeny RT et al. *JAMA Network Open.*

eTable 7 continued

**H2RA & Nitrates**	**1,950**	**11.79**	**2.30**	**1.52**	**3.46**	**7.10E-05**	**1.10E-04**
**Antiparkinson Agents & Opioids**	**1,949**	**18.47**	**4.90**	**3.53**	**6.80**	**2.10E-21**	**8.50E-21**
**Antiparkinson Agents & SGAP**	**1,909**	**17.81**	**2.97**	**2.12**	**4.17**	**2.90E-10**	**6.20E-10**
**Inhaled Glucocorticoids & Opioids**	**1,894**	**10.03**	**3.02**	**1.92**	**4.74**	**1.50E-06**	**2.60E-06**
**Anticonvulsants & Antiparkinson Agents**	**1,874**	**16.55**	**4.04**	**2.84**	**5.75**	**1.00E-14**	**2.70E-14**
**CAAH & Opioids**	**1,865**	**11.26**	**3.67**	**2.39**	**5.64**	**2.60E-09**	**5.40E-09**
TCA & Sedative Hypnotics	1,767	<11	1.74	0.87	3.49	1.20E-01	1.40E-01
**Oral Glucocorticoids & Anticonvulsants**	**1,599**	**20.01**	**5.75**	**4.06**	**8.14**	**6.20E-23**	**2.90E-22**
**Benzodiazepine & Sedative Hypnotics**	**1,532**	**8.49**	**3.14**	**1.82**	**5.42**	**3.70E-05**	**6.10E-05**
**Oral Glucocorticoids & Sedative Hypnotics**	**1,515**	**<11**	**2.09**	**1.13**	**3.89**	**2.00E-02**	**2.60E-02**
**Anticonvulsants & Nitrates**	**1,480**	**9.46**	**1.93**	**1.14**	**3.27**	**1.40E-02**	**1.80E-02**
**H2RA & Sedative Hypnotics**	**1,458**	**<11**	**1.98**	**1.07**	**3.69**	**3.00E-02**	**3.80E-02**
**Anticonvulsants & Benzodiazepine**	**1,407**	**10.66**	**3.09**	**1.86**	**5.13**	**1.30E-05**	**2.20E-05**
**TZD & Opioids**	**1,395**	**15.05**	**6.23**	**4.06**	**9.56**	**6.10E-17**	**1.90E-16**
**H1 Blockers & Loop Diuretics**	**1,327**	**17.33**	**3.59**	**2.38**	**5.41**	**9.50E-10**	**2.00E-09**
**TZD & Anticonvulsants**	**1,282**	**8.58**	**4.21**	**2.33**	**7.60**	**1.90E-06**	**3.30E-06**
**Muscle Relaxers & Opioids**	**1,271**	**14.16**	**6.50**	**4.09**	**10.32**	**2.20E-15**	**6.40E-15**
**Oral Glucocorticoids & H2RA**	**1,243**	**11.26**	**2.90**	**1.72**	**4.90**	**7.00E-05**	**1.10E-04**
Inhaled Glucocorticoids & Sedative Hypnotics	1,242	<11	1.28	0.53	3.08	5.80E-01	6.20E-01
**SSRI/SNRI & H1 Blockers**	**1,241**	**22.56**	**4.64**	**3.20**	**6.73**	**5.40E-16**	**1.60E-15**
Inhaled Glucocorticoids & Anticonvulsants	1,220	<11	1.32	0.59	2.94	5.00E-01	5.40E-01
**Opioids & SGAP**	**1,219**	**32.00**	**3.60**	**2.63**	**4.94**	**1.60E-15**	**4.70E-15**
H2RA & TCA	1,210	<11	1.93	0.92	4.05	8.30E-02	1.00E-01
**Inhaled Glucocorticoids & H2RA**	**1,184**	**<11**	**2.81**	**1.51**	**5.22**	**1.10E-03**	**1.60E-03**
Anticonvulsants & CAAH	1,183	<11	2.12	1.06	4.24	3.40E-02	4.30E-02
**H2RA & SGAP**	**1,140**	**23.69**	**2.21**	**1.52**	**3.23**	**3.90E-05**	**6.40E-05**
**H1 Blockers & Opioids**	**1,139**	**21.07**	**5.76**	**3.86**	**8.61**	**1.10E-17**	**3.70E-17**
**Sedative Hypnotics & SGAP**	**1,030**	**28.15**	**4.17**	**2.89**	**6.00**	**1.80E-14**	**4.90E-14**
**TCA & Benzodiazepines**	**1,006**	**<11**	**3.36**	**1.75**	**6.46**	**2.80E-04**	**4.20E-04**
**Sedative Hypnotics & Nitrates**	**1,002**	**11.98**	**2.55**	**1.45**	**4.49**	**1.20E-03**	**1.70E-03**
**Barbiturates & Thiazide Diuretics**	**967**	**<11**	**2.65**	**1.33**	**5.31**	**5.80E-03**	**8.00E-03**
**Sedative Hypnotics & Antiparkinson Agents**	**844**	**<11**	**3.60**	**1.93**	**6.69**	**5.30E-05**	**8.60E-05**
Muscle Relaxers & Thiazide Diuretics	844	<11	2.22	0.83	5.91	1.10E-01	1.30E-01
Oral Glucocorticoids & TCA	808	<11	1.93	0.80	4.63	1.40E-01	1.60E-01
SSRI/SNRI & Barbiturates	799	<11	1.57	0.71	3.51	2.70E-01	3.10E-01
**Thiazide Diuretics & FGAP**	**797**	**<11**	**2.98**	**1.49**	**5.97**	**2.00E-03**	**2.80E-03**
CAAH & Nitrates	778	<11	1.97	0.94	4.13	7.40E-02	9.00E-02
Inhaled Glucocorticoids & Nitrates	775	<11	1.68	0.76	3.75	2.00E-01	2.30E-01
**Antiparkinson Agents & FGAP**	**768**	**19.53**	**5.44**	**3.27**	**9.03**	**6.00E-11**	**1.30E-10**
**TZD & Nitrates**	**766**	**<11**	**4.04**	**2.10**	**7.77**	**2.80E-05**	**4.70E-05**
**H2RA & Benzodiazepine**	**762**	**<11**	**3.24**	**1.68**	**6.22**	**4.30E-04**	**6.40E-04**
H2RA & TZD	755	<11	1.87	0.60	5.81	2.80E-01	3.10E-01
H2RA & CAAH	748	<11	1.40	0.53	3.73	5.00E-01	5.40E-01
**Sedative Hypnotics & CAAH**	**696**	**<11**	**2.65**	**1.19**	**5.91**	**1.70E-02**	**2.20E-02**
**Anticonvulsants & Barbiturates**	**692**	**23.13**	**7.38**	**4.52**	**12.07**	**1.50E-15**	**4.50E-15**
**Oral Glucocorticoids & Benzodiazepine**	**681**	**19.09**	**5.43**	**3.15**	**9.35**	**1.10E-09**	**2.30E-09**
**TZD & Sedative Hypnotics**	**675**	**<11**	**4.55**	**2.17**	**9.54**	**6.20E-05**	**9.80E-05**
**SSRI/SNRI & FGAP**	**675**	**22.22**	**3.49**	**2.10**	**5.80**	**1.30E-06**	**2.30E-06**
Inhaled Glucocorticoids & TCA	668	<11	2.50	1.04	6.01	4.10E-02	5.20E-02
**Inhaled Glucocorticoids & Benzodiazepine**	**665**	**<11**	**3.91**	**1.96**	**7.83**	**1.20E-04**	**1.80E-04**
**Benzodiazepine & SGAP**	**661**	**49.95**	**6.91**	**4.91**	**9.74**	**2.00E-28**	**1.10E-27**
TZD & TCA	655	<11	1.45	0.36	5.82	6.00E-01	6.40E-01
Oral Glucocorticoids & Nitrates	653	<11	2.10	1.00	4.40	5.00E-02	6.30E-02
**TCA & Antiparkinson Agents**	**618**	**<11**	**2.99**	**1.34**	**6.66**	**7.40E-03**	**1.00E-02**
TCA & Nitrates	615	<11	1.83	0.76	4.39	1.80E-01	2.10E-01
**PPI & Barbiturates**	**602**	**<11**	**2.85**	**1.28**	**6.35**	**1.00E-02**	**1.30E-02**
PPI & Muscle Relaxers	591	<11	2.02	0.65	6.25	2.20E-01	2.50E-01
H2RA & Antiparkinson Agents	581	<11	1.93	0.87	4.29	1.10E-01	1.30E-01
**H2RA & H1 Blockers**	**551**	**<11**	**2.59**	**1.17**	**5.78**	**2.00E-02**	**2.60E-02**
PPI & FGAP	542	<11	1.82	0.87	3.82	1.10E-01	1.30E-01
Sedative Hypnotics & H1 Blockers	528	<11	1.45	0.47	4.51	5.20E-01	5.60E-01
**Anticonvulsants & H1 Blockers**	**524**	**<11**	**4.37**	**2.18**	**8.74**	**3.10E-05**	**5.20E-05**
TCA & SGAP	518	<11	1.03	0.33	3.19	9.60E-01	9.60E-01
**Benzodiazepine & Antiparkinson Agents**	**496**	**<11**	**5.23**	**2.72**	**10.06**	**7.20E-07**	**1.30E-06**
Nitrates & SGAP	492	<11	1.69	0.88	3.25	1.20E-01	1.40E-01
**Oral Glucocorticoids & Antiparkinson Agents**	**486**	**<11**	**3.41**	**1.62**	**7.15**	**1.20E-03**	**1.70E-03**

© 2019 Emeny RT et al. *JAMA Network Open.*

**TCA & CAAH**	**480**	**<11**	**3.23**	**1.35**	**7.77**	**8.70E-03**	**1.20E-02**
Benzodiazepine & Nitrates	467	<11	2.14	0.89	5.15	8.90E-02	1.10E-01
**TZD & CAAH**	**462**	**<11**	**3.83**	**1.44**	**10.20**	**7.30E-03**	**1.00E-02**
Oral Glucocorticoids & CAAH	441	<11	1.10	0.28	4.39	8.90E-01	9.00E-01
**FGAP & SGAP**	**440**	**24.97**	**4.80**	**2.65**	**8.67**	**2.10E-07**	**3.90E-07**
**Benzodiazepine & CAAH**	**426**	**<11**	**3.92**	**1.76**	**8.72**	**8.40E-04**	**1.20E-03**
**Inhaled Glucocorticoids & TZD**	**424**	**<11**	**4.40**	**1.65**	**11.72**	**3.10E-03**	**4.40E-03**
Antiparkinson Agents & Nitrates	406	<11	0.92	0.23	3.67	9.00E-01	9.00E-01
**Inhaled Glucocorticoids & Antiparkinson Age**	**384**	**<11**	**4.47**	**2.01**	**9.96**	**2.50E-04**	**3.80E-04**
**Barbiturates & Loop Diuretics**	**381**	**<11**	**4.86**	**2.61**	**9.03**	**6.00E-07**	**1.10E-06**
SSRISNRI & Muscle Relaxers	366	<11	2.89	0.93	8.95	6.60E-02	8.10E-02
TZD & SGAP	366	<11	1.81	0.58	5.62	3.00E-01	3.30E-01
H1 Blockers & Nitrates	360	<11	2.11	0.79	5.62	1.40E-01	1.60E-01
**FGAP & Loop Diuretics**	**357**	**33.61**	**5.27**	**2.99**	**9.29**	**8.80E-09**	**1.80E-08**
Inhaled Glucocorticoids & SGAP	354	<11	1.36	0.51	3.62	5.40E-01	5.80E-01
Inhaled Glucocorticoids & CAAH	339	<11	0.00	0.00	0.00	7.50E-01	7.80E-01
**TCA & FGAP**	**322**	**<11**	**6.14**	**2.76**	**13.68**	**8.80E-06**	**1.50E-05**
CAAH & SGAP	287	<11	0.39	0.06	2.79	3.50E-01	3.90E-01
**Oral Glucocorticoids & H1 Blockers**	**277**	**<11**	**7.94**	**3.97**	**15.87**	**4.80E-09**	**9.60E-09**
**Antiparkinson Agents & CAAH**	**273**	**<11**	**5.13**	**2.31**	**11.43**	**6.20E-05**	**9.80E-05**
**Oral Glucocorticoids & SGAP**	**267**	**<11**	**2.77**	**1.32**	**5.82**	**7.00E-03**	**9.60E-03**
TCA & H1 Blockers	267	<11	2.10	0.52	8.39	3.00E-01	3.30E-01
Oral Glucocorticoids & TZD	260	<11	1.48	0.21	10.51	7.00E-01	7.40E-01
**Inhaled Glucocorticoids & H1 Blockers**	**252**	**<11**	**8.34**	**3.98**	**17.51**	**2.00E-08**	**4.00E-08**
**Benzodiazepine & H1 Blockers**	**249**	**<11**	**4.25**	**1.60**	**11.34**	**3.80E-03**	**5.30E-03**
TZD & Antiparkinson Agents	249	<11	1.45	0.20	10.28	7.10E-01	7.40E-01
**Anticonvulsants & FGAP**	**227**	**<11**	**4.40**	**1.83**	**10.57**	**9.40E-04**	**1.40E-03**
Muscle Relaxers & Loop Diuretics	214	<11	1.53	0.22	10.89	6.70E-01	7.10E-01
Anticonvulsants & Muscle Relaxers	212	<11	3.80	0.95	15.18	5.90E-02	7.30E-02
Sedative Hypnotics& Muscle Relaxers	207	<11	0.00	0.00	0.00	8.50E-01	8.70E-01
**Opioids & FGAP**	**202**	**<11**	**6.18**	**2.94**	**12.97**	**1.50E-06**	**2.60E-06**
**Barbiturates & Opioids**	**198**	**<11**	**8.16**	**3.89**	**17.12**	**2.90E-08**	**5.60E-08**
TZD & H1 Blockers	197	<11	3.77	0.94	15.06	6.10E-02	7.50E-02
H1 Blockers & CAAH	196	<11	2.89	0.72	11.54	1.30E-01	1.50E-01
H2RA & FGAP	172	<11	1.73	0.43	6.90	4.40E-01	4.80E-01
H1 Blockers & SGAP	160	<11	2.31	0.74	7.16	1.50E-01	1.70E-01
**Barbiturates & Antiparkinson Agents**	**157**	**<11**	**4.08**	**1.31**	**12.65**	**1.50E-02**	**2.00E-02**
**Sedative Hypnotics& FGAP**	**147**	**<11**	**5.01**	**1.88**	**13.36**	**1.30E-03**	**1.90E-03**
**H1 Blockers & Antiparkinson Agents**	**143**	**<11**	**4.68**	**1.51**	**14.52**	**7.50E-03**	**1.00E-02**
TCA & Muscle Relaxers	131	<11	0.00	0.00	0.00	8.80E-01	8.90E-01
**TZD & Benzodiazepine**	**129**	**<11**	**13.43**	**5.04**	**35.80**	**2.10E-07**	**3.90E-07**
Barbiturates & Sedative Hypnotics	127	<11	3.92	0.98	15.67	5.30E-02	6.60E-02
Benzodiazepine & Barbiturates	126	<11	2.96	0.42	21.05	2.80E-01	3.10E-01
**TCA & Barbiturates**	**107**	**<11**	**10.53**	**3.40**	**32.66**	**4.60E-05**	**7.50E-05**
Inhaled Glucocorticoids & Barbiturates	105	<11	0.00	0.00	0.00	8.70E-01	8.80E-01
**FAD pairs with <100PY**	-	-	**2.67**	**1.66**	**4.29**	**5.40E-05**	**8.70E-05**
**3+FAD**	-	-	**4.58**	**4.43**	**4.73**	**0.00E+00**	**0.00E+00**
**0 Non-FAD**	-	-					
**1 Non-FAD**	-	-	**0.94**	**0.91**	**0.97**	**1.20E-04**	**1.80E-04**
**2 Non-FAD**	-	-	**0.84**	**0.82**	**0.87**	**1.20E-23**	**5.80E-23**
**3+ Non-FAD**	-	-	**0.75**	**0.72**	**0.77**	**4.80E-80**	**5.20E-79**

Exposure indicator variables for fracture associated drug (FAD) use reflect person days with any of 21 individual FADs and 210 possible two-way combination of these drugs (included as a specific exposure in most cases, but aggregated to "FAD pairs with <100 PY" if cohort specific observation < 100 person years). "3+FAD" is an indicator variable for person days with three-or-more FADs. The reference exposure was zero FADs. Non-FADs are 552 drug ingredients that are not FADs aggregated into status of receipt of any 1 non-FAD expsoure (1 Non-FAD), 2 concurrent non-FAD exposures (2 Non-FAD) or 3+ Non-FAD exposure classified for each person, for each day of observation. Medicaid eligibility and Medicare Part D low income subsidy are two poverty indicators. Long-term care is defined as 50% or more of annual prescriptions dispensed by a long-term care pharmacy. The three categories of drugs protective of fracture are: 1. osteoporosis treatments: oral and injected bisphosphonates, calcitonin, denosumab, parathyroid hormone, 2. Estrogens- systemic estrogens, selective estrogen receptor modulators, and 3. beta-blockers. SSRI/SNRI = combined drug group of selective serotonin reuptake inhibitors and selective norepinephrine reuptake inhibitors. PPI= proton pump inhibitors, FGAP = first generation antipsychotic. SGAP = second generation antipsychotic. TCA= tricyclic antidepressants. H2RA is Histamine-2 Receptor Antagonists. TZD = Thiazolidinediones. H1 Blockers = 1st Generation Antihistamines. CAAH = Central Acting Antihypertensives. Sedative Hypnotics include only non-benzodiazepine drugs. The Tobacco/Chronic Obstructive Lung Disease is an aggregate indicator of these diagnoses. Bold if False Discovery Rate corrected P value < 0.05. Italicized if population-level impact criteria were met.

© 2019 Emeny RT et al. *JAMA Network Open.*

eTable 8. Cox Proportional Hazard Model Output: Hip Fracture Risk Associated With Fracture-Associated Drugs Among Men

In 20% Medicare sample, person-years of exposure and crude fracture rate per exposure group, adjusted for sociodemographic factors, comorbidities, non-fracture-associated drug use (person-years, 4 160 502).

			Cox Regression Model (Hip Fracture n = 12,317)				
**Parameter**	**Person Years**	**Fracture Rate (# Fracture per 1000 PY)**	**Hazard Ratio (HR)**	**Lower 95% Confidence Interval**	**Higher 95% Confidence Interval**	**p-value**	**False Discovery Rate p-value**
**Age (reference 67-69)**							
**Age 70-74**	-	-	**1.19**	**1.12**	**1.27**	**9.60E-08**	**3.00E-07**
**Age 75-79**	-	-	**1.84**	**1.72**	**1.96**	**1.10E-77**	**2.50E-76**
**Age 80-84**	-	-	**2.98**	**2.80**	**3.18**	**1.00E-248**	**4.00E-247**
**Age 85+**	-	-	**5.29**	**4.96**	**5.64**	**0.00E+00**	**0.00E+00**
**Race/Ethnicity (reference white)**							
**Black**	-	-	**0.56**	**0.51**	**0.61**	**6.20E-38**	**7.90E-37**
**Hispanic**	-	-	**0.71**	**0.65**	**0.77**	**5.40E-16**	**3.30E-15**
**Asian**	-	-	**0.52**	**0.45**	**0.59**	**1.80E-23**	**1.20E-22**
Other	-	-	0.92	0.77	1.08	3.00E-01	3.70E-01
**Low Income Subsidy**	-	-	**1.18**	**1.08**	**1.28**	**1.20E-04**	**2.60E-04**
**Original Medicare eligibility (%)**							
Disability	-	-	1.03	0.33	3.18	9.70E-01	9.70E-01
Aged	-	-	0.87	0.28	2.71	8.10E-01	9.00E-01
**Long term care**	-	-	**1.43**	**1.36**	**1.51**	**9.90E-43**	**1.50E-41**
**Dual eligible**	-	-	**0.85**	**0.78**	**0.93**	**2.70E-04**	**5.70E-04**
Chronic Conditions							
**Osteoporosis/Osteopenia**	-	-	**4.44**	**4.12**	**4.78**	**0.00E+00**	**0.00E+00**
**Dementia**	-	-	**3.74**	**3.55**	**3.94**	**0.00E+00**	**0.00E+00**
Obesity	-	-	0.94	0.87	1.02	1.20E-01	1.60E-01
**Depression**	-	-	**1.23**	**1.16**	**1.31**	**3.60E-12**	**1.60E-11**
**Serious Mental Illness**	-	-	**1.34**	**1.23**	**1.45**	**8.50E-13**	**4.10E-12**
**Alcohol Abuse**	-	-	**1.69**	**1.48**	**1.93**	**2.40E-14**	**1.30E-13**
**Tobacco/Chronic Lung Disease**	-	-	**2.52**	**2.41**	**2.63**	**0.00E+00**	**0.00E+00**
**Diabetes**	-	-	**0.87**	**0.83**	**0.90**	**6.60E-13**	**3.30E-12**
Liver Disease	-	-	1.07	0.96	1.19	2.10E-01	2.70E-01
**Pancreatic Disease**	-	-	**0.88**	**0.80**	**0.96**	**4.80E-03**	**8.10E-03**
Inflammatory Bowel Disease	-	-	0.99	0.87	1.12	8.50E-01	9.30E-01
Rheumatologic Disease	-	-	0.98	0.93	1.04	5.70E-01	6.50E-01
Spinal Cord Disease or Injury	-	-	1.09	1.00	1.19	4.40E-02	6.10E-02
Serious Neurologic Disease	-	-	1.01	0.97	1.06	6.20E-01	6.90E-01
**Parkinson's/Huntington's Disease**	-	-	**1.09**	**1.01**	**1.16**	**1.80E-02**	**2.70E-02**
**Seizure Disorder**	-	-	**0.90**	**0.85**	**0.96**	**1.70E-03**	**3.10E-03**
Congestive Heart Failure	-	-	**1.06**	**1.02**	**1.11**	**4.60E-03**	**7.80E-03**
**Coronary Artery Disease**	-	-	**0.91**	**0.87**	**0.95**	**4.40E-06**	**1.10E-05**
Cerebrovascular Disease	-	-	1.03	0.99	1.08	1.80E-01	2.30E-01
Peripheral Vascular Disease	-	-	1.01	0.97	1.05	5.90E-01	6.70E-01
**Traumatic Brain Injury**	-	-	**1.29**	**1.19**	**1.39**	**1.10E-10**	**4.30E-10**
**Amputee**	-	-	**1.36**	**1.19**	**1.55**	**6.40E-06**	**1.60E-05**
Estrogen	-	-	0.52	0.22	1.25	1.40E-01	1.80E-01
**Osteoporosis medications**	-	-	**0.86**	**0.75**	**0.97**	**1.60E-02**	**2.50E-02**
**Beta Blockers**	-	-	**1.28**	**1.23**	**1.33**	**2.80E-31**	**2.70E-30**
**Single FAD Exposures**							
**Thiazide Diuretics**	**356,819**	**1.74**	**1.65**	**1.51**	**1.81**	**5.80E-28**	**4.70E-27**
**Proton Pump Inhibitors (PPI)**	**165,092**	**2.51**	**1.89**	**1.70**	**2.09**	**1.90E-32**	**2.20E-31**
**Loop Diuretics**	**87,781**	**5.72**	**2.77**	**2.51**	**3.06**	**1.80E-88**	**5.20E-87**
**SSRI/SNRI**	**61,141**	**5.74**	**2.96**	**2.64**	**3.32**	**2.30E-78**	**5.90E-77**
**Opioids**	**47,153**	**5.41**	**3.83**	**3.36**	**4.36**	**3.90E-91**	**1.30E-89**
**Anticonvulsants**	**38,090**	**4.17**	**2.66**	**2.26**	**3.13**	**5.30E-32**	**5.80E-31**
**Thiazolidinediones (TZD)**	**35,171**	**2.33**	**2.48**	**1.99**	**3.10**	**1.10E-15**	**6.50E-15**
**Nitrates**	**32,805**	**3.17**	**1.80**	**1.48**	**2.19**	**6.30E-09**	**2.10E-08**
**Sedative Hypnotics**	**29,598**	**2.03**	**1.51**	**1.17**	**1.95**	**1.70E-03**	**3.10E-03**
**Inhaled Glucocorticoids**	**28,431**	**2.64**	**1.92**	**1.52**	**2.41**	**3.20E-08**	**1.00E-07**
**Oral Glucocorticoids**	**24,530**	**3.71**	**2.50**	**2.03**	**3.09**	**1.10E-17**	**6.80E-17**
**H2 Receptor Antagonists (H2RA)**	**24,328**	**2.84**	**1.84**	**1.44**	**2.33**	**6.80E-07**	**1.90E-06**
**Anti Parkinson's Agents**	**18,174**	**8.58**	**4.23**	**3.57**	**5.01**	**1.30E-62**	**2.70E-61**
**Benzodiazepine**	**11,556**	**2.25**	**1.62**	**1.10**	**2.38**	**1.50E-02**	**2.30E-02**
Tricyclic Antidepressants (TCA)	9,848	2.03	1.55	1.00	2.40	5.20E-02	7.20E-02
Central Acting Antihypertensives (CAAH)	9,276	2.16	1.59	1.03	2.48	3.80E-02	5.40E-02
**2nd Generation Antispychotics (SGAP)**	**9,002**	**11.55**	**2.53**	**2.07**	**3.08**	**6.80E-20**	**4.30E-19**
1st Generation Antihistamines (H1 Blockers)	5,108	3.13	1.70	1.04	2.77	3.50E-02	5.00E-02
Barbiturates	2,841	<11	1.78	0.89	3.56	1.10E-01	1.50E-01
**1st Generation Antipsychotics (FGAP)**	**1,644**	**7.91**	**2.73**	**1.58**	**4.71**	**3.10E-04**	**6.40E-04**
Muscle Relaxers	1,591	<11	1.65	0.53	5.13	3.80E-01	4.50E-01

© 2019 Emeny RT et al. *JAMA Network Open.*

Appendix Table 8 Continued

Concurrent Exposure to 2 FADs							
PPI & Thiazide Diuretics	50,082	1.88	1.77	1.44	2.18	6.20E-08	2.00E-07
PPI & Loop Diuretics	16,398	7.56	3.15	2.62	3.78	1.40E-34	1.70E-33
SSRI/SNRI & Thiazide Diuretics	15,502	3.48	2.51	1.92	3.29	2.60E-11	1.10E-10
PPI & SSRI/SNRI	14,657	8.73	3.94	3.29	4.72	2.00E-50	3.50E-49
TZD & Thiazide Diuretics	13,126	2.44	3.00	2.11	4.25	8.50E-10	3.00E-09
Thiazide Diuretics & Opioids	12,391	5.57	4.98	3.92	6.33	4.20E-39	5.70E-38
Thiazide Diuretics & Loop Diuretics	10,358	6.47	4.18	3.28	5.34	1.80E-30	1.60E-29
PPI & Opioids	9,930	7.25	4.97	3.92	6.29	1.60E-40	2.30E-39
Anticonvulsants & Thiazide Diuretics	9,928	3.32	2.81	1.99	3.97	4.20E-09	1.40E-08
Nitrates & Loop Diuretics	8,944	7.94	3.45	2.71	4.37	3.30E-24	2.40E-23
Thiazide Diuretics & Nitrates	7,784	3.08	2.11	1.41	3.16	2.90E-04	6.10E-04
H2RA & Thiazide Diuretics	7,677	2.47	2.18	1.39	3.42	7.40E-04	1.50E-03
PPI & Anticonvulsants	7,433	5.38	2.94	2.15	4.02	1.70E-11	7.10E-11
Sedative Hypnotics & Thiazide Diuretics	7,333	2.05	1.83	1.10	3.04	2.00E-02	3.00E-02
PPI & Nitrates	7,229	3.60	1.87	1.27	2.76	1.50E-03	2.90E-03
CAAH & Thiazide Diuretics	7,042	2.13	2.07	1.25	3.44	5.00E-03	8.30E-03
SSRI/SNRI & Loop Diuretics	6,777	12.54	3.72	2.98	4.63	8.90E-32	9.20E-31
Inhaled Glucocorticoids & Thiazide Diuretics	6,751	2.67	2.35	1.48	3.75	3.00E-04	6.20E-04
PPI & Sedative Hypnotics	6,112	3.76	2.84	1.88	4.28	6.70E-07	1.90E-06
Opioids & Loop Diuretics	5,652	13.80	6.93	5.52	8.70	2.70E-62	5.20E-61
Inhaled Glucocorticoids & PPI	5,557	3.96	2.72	1.79	4.14	3.10E-06	7.90E-06
Anticonvulsants & Opioids	5,262	8.17	5.43	4.01	7.35	6.80E-28	5.40E-27
Oral Glucocorticoids & Thiazide Diuretics	5,060	2.37	1.92	1.09	3.39	2.40E-02	3.60E-02
Anticonvulsants & Loop Diuretics	5,013	7.58	3.16	2.29	4.36	3.00E-12	1.30E-11
Anticonvulsants & SSRI/SNRI	4,717	7.00	3.00	2.12	4.23	4.90E-10	1.80E-09
Oral Glucocorticoids & PPI	4,543	6.38	3.81	2.64	5.50	9.50E-13	4.40E-12
SSRI/SNRI & SGAP	4,445	15.52	3.57	2.80	4.55	6.30E-25	4.70E-24
SSRI/SNRI & Opioids	4,443	13.28	6.26	4.83	8.12	1.90E-43	3.10E-42
SSRI/SNRI & Sedative Hypnotics	4,192	8.35	5.12	3.67	7.16	1.00E-21	6.70E-21
PPI & TZD	4,077	2.94	3.04	1.72	5.36	1.30E-04	2.80E-04
Antiparkinson Agents & Thiazide Diuretics	3,634	6.88	4.63	3.11	6.88	3.80E-14	2.00E-13
TZD & Loop Diuretics	3,589	6.41	5.11	3.38	7.71	8.80E-15	5.00E-14
PPI & H2RA	3,446	<11	1.86	0.93	3.71	8.10E-02	1.10E-01
Sedative Hypnotics & Opioids	3,347	5.97	4.39	2.82	6.81	4.80E-11	1.90E-10
Inhaled Glucocorticoids & Loop Diuretics	3,311	7.25	3.22	2.15	4.81	1.40E-08	4.60E-08
H2RA & Loop Diuretics	3,268	6.43	2.86	1.86	4.40	1.70E-06	4.50E-06
PPI & Antiparkinson Agents	2,964	8.43	3.48	2.34	5.18	7.40E-10	2.60E-09
SSRI/SNRI & Antiparkinson Agents	2,963	15.86	5.81	4.33	7.80	9.20E-32	9.20E-31
Oral Glucocorticoids & Loop Diuretics	2,900	12.07	5.08	3.64	7.11	2.00E-21	1.30E-20
TCA & Thiazide Diuretics	2,882	<11	1.52	0.63	3.66	3.50E-01	4.20E-01
Benzodiazepine & Thiazide Diuretics	2,875	4.52	3.88	2.25	6.69	1.10E-06	2.90E-06
PPI & Benzodiazepine	2,634	4.56	3.17	1.80	5.59	6.90E-05	1.50E-04
CAAH & Loop Diuretics	2,560	5.86	3.90	2.35	6.49	1.50E-07	4.50E-07
SSRI/SNRI & Benzodiazepine	2,462	11.78	7.12	4.93	10.28	1.20E-25	9.20E-25
Sedative Hypnotics & Loop Diuretics	2,346	10.66	4.56	3.07	6.77	5.20E-14	2.70E-13
H2RA & SSRI/SNRI	2,263	10.17	3.70	2.45	5.59	4.80E-10	1.80E-09
PPI & TCA	2,143	<11	1.38	0.52	3.69	5.20E-01	6.00E-01
SSRI/SNRI & Nitrates	2,045	10.76	3.89	2.56	5.93	2.50E-10	9.50E-10
Oral Glucocorticoids & Opioids	1,877	6.93	4.43	2.57	7.65	9.20E-08	2.90E-07
Antiparkinson Agents & Loop Diuretics	1,798	21.14	6.67	4.82	9.23	2.70E-30	2.40E-29
H2RA & Opioids	1,797	10.02	6.21	3.90	9.88	1.30E-14	7.30E-14
PPI & SGAP	1,715	13.99	2.51	1.68	3.76	8.20E-06	2.00E-05
Benzodiazepine & Opioids	1,699	8.83	6.40	3.85	10.64	8.60E-13	4.10E-12
TZD & SSRI/SNRI	1,683	8.91	6.21	3.73	10.32	2.00E-12	9.20E-12
Nitrates & Opioids	1,679	8.93	5.00	3.01	8.32	5.50E-10	2.00E-09
H2RA & Nitrates	1,627	<11	2.90	1.51	5.59	1.40E-03	2.70E-03
Inhaled Glucocorticoids & Oral Glucocorticoids	1,615	<11	1.42	0.53	3.78	4.90E-01	5.70E-01
Anticonvulsants & Sedative Hypnotics	1,524	<11	1.36	0.44	4.21	6.00E-01	6.80E-01
Inhaled Glucocorticoids & SSRI/SNRI	1,514	7.27	3.16	1.75	5.73	1.40E-04	3.00E-04
Oral Glucocorticoids & SSRI/SNRI	1,408	9.94	3.91	2.31	6.62	3.80E-07	1.10E-06
H2RA & Anticonvulsants	1,399	<11	2.82	1.46	5.44	1.90E-03	3.40E-03
Antiparkinson Agents & SGAP	1,367	15.36	3.43	2.23	5.29	2.40E-08	7.90E-08
Anticonvulsants & SGAP	1,343	11.17	2.54	1.52	4.23	3.50E-04	7.20E-04
PPI & CAAH	1,341	<11	2.98	1.34	6.65	7.50E-03	1.20E-02
Anticonvulsants & Antiparkinson Agents	1,292	10.06	4.05	2.34	7.00	5.60E-07	1.60E-06
TZD & Anticonvulsants	1,231	<11	4.11	1.71	9.88	1.60E-03	3.00E-03
H1 Blockers & Thiazide Diuretics	1,228	<11	1.76	0.57	5.45	3.30E-01	4.00E-01
TZD & Opioids	1,202	<11	4.21	1.75	10.14	1.30E-03	2.50E-03
Anticonvulsants & Nitrates	1,198	<11	1.61	0.60	4.30	3.40E-01	4.10E-01
Thiazide Diuretics & SGAP	1,170	11.11	3.74	2.17	6.46	2.20E-06	5.70E-06
Inhaled Glucocorticoids & Opioids	1,154	<11	4.53	2.26	9.08	2.00E-05	4.80E-05
SGAP & Loop Diuretics	1,139	23.70	3.56	2.43	5.21	7.20E-11	2.80E-10
Antiparkinson Agents & Opioids	1,085	11.06	5.52	3.12	9.74	4.00E-09	1.40E-08
Muscle Relaxers & Opioids	1,058	<11	5.42	2.43	12.08	3.60E-05	8.30E-05
TCA & Opioids	1,057	<11	5.97	2.98	11.96	4.60E-07	1.30E-06
TZD & Nitrates	1,057	<11	3.84	1.60	9.24	2.70E-03	4.70E-03
PPI & H1 Blockers	1,053	<11	4.46	2.23	8.93	2.40E-05	5.70E-05
Anticonvulsants & TCA	1,022	<11	2.07	0.67	6.42	2.10E-01	2.70E-01
SSRI/SNRI & TCA	992	<11	1.83	0.59	5.69	2.90E-01	3.60E-01
Benzodiazepine & Loop Diuretics	985	<11	2.27	0.94	5.47	6.70E-02	9.10E-02
Oral Glucocorticoids & Anticonvulsants	908	<11	3.62	1.62	8.07	1.70E-03	3.10E-03

© 2019 Emeny RT et al. *JAMA Network Open.*

Appendix Table 8 Continued

[illegible]	[illegible]	[illegible]	[illegible]	[illegible]	[illegible]	[illegible]	[illegible]
Sedative Hypnotics & Nitrates	874	<11	1.76	0.57	5.46	3.30E-01	4.00E-01
Benzodiazepine & Sedative Hypnotics	850	<11	2.70	0.87	8.38	8.60E-02	1.20E-01
**TCA & Loop Diuretics**	**844**	**<11**	**3.31**	**1.37**	**7.95**	**7.60E-03**	**1.20E-02**
Inhaled Glucocorticoids & H2RA	824	<11	2.07	0.67	6.43	2.10E-01	2.70E-01
**Oral Glucocorticoids & Sedative Hypnotics**	**805**	**<11**	**3.94**	**1.64**	**9.48**	**2.20E-03**	**3.80E-03**
H2RA & Sedative Hypnotics	784	<11	2.69	1.01	7.17	4.80E-02	6.70E-02
CAAH & Opioids	772	<11	0.95	0.13	6.78	9.60E-01	9.70E-01
Inhaled Glucocorticoids & Anticonvulsants	769	<11	**2.96**	**1.11**	**7.90**	**3.00E-02**	**4.40E-02**
**Inhaled Glucocorticoids & Sedative Hypnotics**	**743**	**<11**	**4.53**	**1.88**	**10.89**	**7.50E-04**	**1.50E-03**
**Barbiturates & Thiazide Diuretics**	**736**	**<11**	**4.04**	**1.68**	**9.71**	**1.80E-03**	**3.20E-03**
**Anticonvulsants & Barbiturates**	**701**	**<11**	**10.03**	**5.38**	**18.69**	**3.90E-13**	**2.00E-12**
**Anticonvulsants & Benzodiazepine**	**697**	**<11**	**3.78**	**1.42**	**10.08**	**8.00E-03**	**1.30E-02**
Inhaled Glucocorticoids & Nitrates	696	<11	**2.95**	**1.10**	**7.86**	**3.10E-02**	**4.50E-02**
**H2RA & TZD**	**691**	**<11**	**3.65**	**1.18**	**11.34**	**2.50E-02**	**3.70E-02**
Oral Glucocorticoids & H2RA	684	<11	**2.99**	**1.12**	**7.97**	**2.90E-02**	**4.30E-02**
**TZD & Sedative Hypnotics**	**653**	**<11**	**5.96**	**2.23**	**15.91**	**3.60E-04**	**7.30E-04**
TCA & Sedative Hypnotics	653	<11	**3.42**	**1.10**	**10.62**	**3.30E-02**	**4.80E-02**
**SSRI/SNRI & CAAH**	**623**	**<11**	**3.81**	**1.58**	**9.16**	**2.90E-03**	**5.00E-03**
**Sedative Hypnotics & Antiparkinson Agents**	**596**	**<11**	**5.51**	**2.47**	**12.28**	**3.10E-05**	**7.20E-05**
**Oral Glucocorticoids & Nitrates**	**576**	**<11**	**4.07**	**1.69**	**9.79**	**1.70E-03**	**3.10E-03**
**Oral Glucocorticoids & Antiparkinson Agents**	**534**	**<11**	**8.65**	**4.64**	**16.13**	**1.20E-11**	**5.10E-11**
**H2RA & SGAP**	**525**	**22.87**	**4.59**	**2.60**	**8.11**	**1.50E-07**	**4.50E-07**
**Antiparkinson Agents & FGAP**	**516**	**<11**	**4.01**	**1.50**	**10.70**	**5.60E-03**	**9.20E-03**
PPI & Barbiturates	507	<11	2.76	0.89	8.55	7.90E-02	1.10E-01
Anticonvulsants & CAAH	507	<11	1.23	0.17	8.73	8.40E-01	9.20E-01
**H1 Blockers & Loop Diuretics**	**489**	**<11**	**6.41**	**3.20**	**12.84**	**1.60E-07**	**4.80E-07**
**Antiparkinson Agents & Nitrates**	**475**	**<11**	**5.37**	**2.55**	**11.30**	**9.40E-06**	**2.30E-05**
**Sedative Hypnotics & SGAP**	**473**	**<11**	**3.90**	**1.94**	**7.81**	**1.30E-04**	**2.80E-04**
H2RA & Antiparkinson Agents	466	<11	2.20	0.71	6.83	1.70E-01	2.20E-01
**Benzodiazepine & Antiparkinson Agents**	**462**	**<11**	**3.99**	**1.28**	**12.40**	**1.70E-02**	**2.60E-02**
**Opioids & SGAP**	**439**	**<11**	**4.83**	**2.59**	**8.99**	**7.10E-07**	**1.90E-06**
**H1 Blockers & Opioids**	**433**	**<11**	**4.85**	**1.82**	**12.92**	**1.60E-03**	**3.00E-03**
Inhaled Glucocorticoids & TZD	408	<11	2.50	0.35	17.75	3.60E-01	4.30E-01
CAAH & Nitrates	**401**	**<11**	0.00	0.00	0.00	9.30E-01	9.70E-01
H2RA & TCA	379	<11	1.74	0.25	12.38	5.80E-01	6.60E-01
TZD & TCA	373	<11	2.69	0.38	19.09	3.20E-01	3.90E-01
Benzodiazepine & Nitrates	368	<11	1.26	0.18	8.98	8.10E-01	9.00E-01
H2RA & Benzodiazepine	365	<11	1.64	0.23	11.68	6.20E-01	6.90E-01
**SSRI/SNRI & Barbiturates**	**344**	**<11**	**5.34**	**2.00**	**14.26**	**8.20E-04**	**1.60E-03**
**Oral Glucocorticoids & Benzodiazepine**	**338**	**<11**	**9.70**	**4.03**	**23.35**	**3.90E-07**	**1.10E-06**
Muscle Relaxers & Thiazide Diuretics	331	<11	2.98	0.42	21.16	2.80E-01	3.50E-01
**Benzodiazepine & SGAP**	**323**	**<11**	**4.73**	**1.96**	**11.38**	**5.30E-04**	**1.10E-03**
**Barbiturates & Loop Diuretics**	**314**	**<11**	**10.07**	**5.23**	**19.40**	**5.00E-12**	**2.20E-11**
Inhaled Glucocorticoids & Benzodiazepine	311	<11	1.95	0.27	13.82	5.10E-01	5.90E-01
TCA & Nitrates	**303**	**<11**	0.00	0.00	0.00	9.30E-01	9.70E-01
Oral Glucocorticoids & TZD	**297**	**<11**	0.00	0.00	0.00	9.40E-01	9.70E-01
H2RA & CAAH	**295**	**<11**	0.00	0.00	0.00	9.30E-01	9.70E-01
**SSRI/SNRI & H1 Blockers**	**290**	**<11**	**6.19**	**2.57**	**14.90**	**4.70E-05**	**1.10E-04**
**TCA & Benzodiazepines**	**287**	**<11**	**11.87**	**4.45**	**31.67**	**7.70E-07**	**2.10E-06**
**TZD & CAAH**	**287**	**<11**	**7.04**	**1.76**	**28.17**	**5.80E-03**	**9.50E-03**
**SSRI/SNRI & FGAP**	**285**	**<11**	**3.88**	**1.46**	**10.36**	**6.70E-03**	**1.10E-02**
TCA & Antiparkinson Agents	283	<11	4.28	1.07	17.15	4.00E-02	5.70E-02
Inhaled Glucocorticoids & Antiparkinson Agents	**282**	**<11**	0.00	0.00	0.00	9.30E-01	9.70E-01
**Nitrates & SGAP**	**279**	**<11**	**3.07**	**1.28**	**7.39**	**1.20E-02**	**1.90E-02**
PPI & Muscle Relaxers	**275**	**<11**	0.00	0.00	0.00	9.50E-01	9.70E-01
**TZD & Antiparkinson Agents**	**264**	**<11**	**9.58**	**3.08**	**29.75**	**9.30E-05**	**2.10E-04**
Thiazide Diuretics & FGAP	**263**	**<11**	0.00	0.00	0.00	9.30E-01	9.70E-01
Sedative Hypnotics & CAAH	260	<11	2.79	0.39	19.84	3.00E-01	3.70E-01
Oral Glucocorticoids & TCA	**237**	**<11**	0.00	0.00	0.00	9.40E-01	9.70E-01
H2RA & H1 Blockers	236	<11	2.04	0.29	14.48	4.80E-01	5.60E-01
**FGAP & SGAP**	235	<11	**3.74**	**1.21**	**11.63**	**2.20E-02**	**3.30E-02**
**PPI & FGAP**	**212**	**<11**	**7.42**	**3.09**	**17.87**	**7.70E-06**	**1.90E-05**
H1 Blockers & Nitrates	207	<11	2.15	0.30	15.24	4.50E-01	5.30E-01
**TZD & SGAP**	**203**	**<11**	**5.07**	**1.63**	**15.75**	**5.00E-03**	**8.30E-03**
Inhaled Glucocorticoids & TCA	**201**	**<11**	0.00	0.00	0.00	9.50E-01	9.70E-01
**Anticonvulsants & H1 Blockers**	**198**	**<11**	**5.32**	**1.33**	**21.29**	**1.80E-02**	**2.70E-02**
TCA & SGAP	**189**	**<11**	0.00	0.00	0.00	9.30E-01	9.70E-01
**Barbiturates & Antiparkinson Agents**	**184**	**<11**	**6.22**	**1.55**	**24.93**	**9.80E-03**	**1.50E-02**
Oral Glucocorticoids & CAAH	**183**	**<11**	0.00	0.00	0.00	9.50E-01	9.70E-01
Inhaled Glucocorticoids & SGAP	183	<11	2.26	0.56	9.05	2.50E-01	3.10E-01
Sedative Hypnotics & H1 Blockers	**182**	**<11**	0.00	0.00	0.00	9.50E-01	9.70E-01
**Inhaled Glucocorticoids & CAAH**	**176**	**<11**	**8.71**	**2.18**	**34.86**	**2.20E-03**	**3.80E-03**
Anticonvulsants & FGAP	156	<11	4.22	1.05	16.93	4.20E-02	5.90E-02
Oral Glucocorticoids & H1 Blockers	144	<11	4.11	0.58	29.21	1.60E-01	2.10E-01
Barbiturates & Opioids	**143**	**<11**	0.00	0.00	0.00	9.50E-01	9.70E-01
TCA & CAAH	**140**	**<11**	0.00	0.00	0.00	9.60E-01	9.70E-01
Benzodiazepine & CAAH	**137**	**<11**	0.00	0.00	0.00	9.60E-01	9.70E-01
Antiparkinson Agents & CAAH	130	<11	3.19	0.45	22.68	2.50E-01	3.10E-01
Inhaled Glucocorticoids & H1 Blockers	128	<11	4.48	0.63	31.85	1.30E-01	1.70E-01
**TZD & Benzodiazepine**	**126**	**<11**	**17.98**	**4.49**	**71.98**	**4.40E-05**	**1.00E-04**
TZD & H1 Blockers	122	<11	6.41	0.90	45.55	6.30E-02	8.60E-02
FGAP & Loop Diuretics	**117**	**<11**	0.00	0.00	0.00	9.30E-01	9.70E-01
Oral Glucocorticoids & SGAP	**113**	**<11**	0.00	0.00	0.00	9.30E-01	9.70E-01
Anticonvulsants & Muscle Relaxers	**111**	**<11**	0.00	0.00	0.00	9.70E-01	9.70E-01

© 2019 Emeny RT et al. *JAMA Network Open.*

Appendix Table 8 Continued

**FAD pairs with <100PY**	-	-	**2.43**	**1.38**	**4.30**	**2.10E-03**	**3.70E-03**
**3+FAD**	-	-	**5.41**	**5.08**	**5.76**	**0.00E+00**	**0.00E+00**
**0 Non-FAD**	-	-	1.00	-	-	-	-
**1 Non-FAD**	-	-	**0.91**	**0.85**	**0.97**	**3.20E-03**	**5.50E-03**
**2 Non-FAD**	-	-	**0.86**	**0.80**	**0.91**	**2.20E-06**	**5.70E-06**
**3+ Non-FAD**	-	-	**0.72**	**0.68**	**0.76**	**4.90E-29**	**4.20E-28**

Exposure indicator variables for fracture associated drug (FAD) use reflect person days with any of 21 individual FADs and 210 possible two-way combination of these drugs (included as a specific exposure in most cases, but aggregated to "FAD pairs with <100 PY" if cohort specific observation < 100 person years). "3+FAD" is an indicator variable for person days with three-or-more FADs. The reference exposure was zero FADs. Non-FADs are 552 drug ingredients that are not FADs aggregated into status of receipt of any 1 non-FAD expsoure (1 Non-FAD), 2 concurrent non-FAD exposures (2 Non-FAD) or 3+ Non-FAD exposure classified for each person, for each day of observation. Medicaid eligibility and Medicare Part D low income subsidy are two poverty indicators. Long-term care is defined as 50% or more of annual prescriptions dispensed by a long-term care pharmacy. The three categories of drugs protective of fracture are: 1. osteoporosis treatments: oral and injected bisphosphonates, calcitonin, denosumab, parathyroid hormone, 2. Estrogens-systemic estrogens, selective estrogen receptor modulators, and 3. beta-blockers. SSRI/SNRI = combined drug group of selective serotonin reuptake inhibitors and selective norepinephrine reuptake inhibitors. PPI= proton pump inhibitors, FGAP = first generation antipsychotic. SGAP = second generation antipsychotic. TCA= tricyclic antidepressants. H2RA is Histamine-2 Receptor Antagonists. TZD = Thiazolidinediones. H1 Blockers = 1st Generation Antihistamines. CAAH = Central Acting Antihypertensives. Sedative Hypnotics include only non-benzodiazepine drugs. The Tobacco/Chronic Obstructive Lung Disease is an aggregate indicator of these diagnoses. Bold if False Discovery Rate corrected P value < 0.05. Italicized if population-level impact criteria were met.

© 2019 Emeny RT et al. *JAMA Network Open.*
